# The effects of transcranial magnetic stimulation in motor symptoms of Parkinson’s disease: an overview of systematic reviews with meta-analysis

**DOI:** 10.1007/s10072-025-08189-5

**Published:** 2025-04-16

**Authors:** Daniele Conte, Anna Roman, Yvonne Beorchia, Chiara Pinzini, Luigi Castriotta, Mariarosaria Valente

**Affiliations:** 1 Department of Medicine (DMED), University of Udine, Via Colugna 50, Udine, 33100 Italy; 2 School of Physiotherapy, Department of Medicine (DMED), University of Udine, Udine, Italy; 3 Institute of Hygiene and Evaluative Epidemiology, Friuli Centrale University Health Authority, Udine, Italy; 4 Institute of Physical Medicine and Rehabilitation “Gervasutta”, Friuli Centrale University Health Authority, Udine, Italy; 5 Central Directorate for Health, Social Policies and Disability, Friuli Venezia Giulia Region, Trieste, Italy; 6 Clinical Neurology Unit, Friuli Centrale University Health Authority, Udine, Italy

**Keywords:** Parkinson’s disease, Neurodegenerative diseases, Transcranial magnetic stimulation, Motor skills disorders; gait disorders, Overview

## Abstract

**Background:**

Parkinson’s disease is a progressive neurodegenerative disorder that causes significant motor function limitations, substantially impacting the quality of life of affected individuals and their caregivers. While the currently available pharmacological therapy with levodopa can alleviate symptoms, identifying a treatment that achieves similar results with fewer adverse effects would be highly beneficial. Transcranial Magnetic Stimulation is a non-invasive stimulation of brain tissue that generates a magnetic field to modulate cortical excitability. To date, it has primarily been validated for the treatment of psychiatric conditions, but it is increasingly being used in the management of movement disorders.

**Objective:**

Although several systematic reviews with meta-analysis have been conducted on this topic, discrepancies remain in their findings. To address these inconsistencies, we conducted this overview of systematic reviews with meta-analyses to synthesise the available evidence and provide a comprehensive summary that can guide clinicians in their practice.

**Results:**

Evidence from 21 systematic reviews with meta-analyses, including 107 unique primary studies, suggests, with low to moderate certainty, that high-frequency stimulation of the primary and supplementary motor cortex significantly improves general motor impairment, gait, functional mobility, and balance in patients with Parkinson’s disease, with minimal side effects. Other stimulation parameters, such as a higher number of sessions, a greater number of pulses per session, and the use of the F8 coil type, appear to enhance these effects. However, further research is needed to strengthen these findings. Currently, definitive conclusions cannot be drawn regarding the influence of patient characteristics on treatment outcomes.

**Supplementary Information:**

The online version contains supplementary material available at 10.1007/s10072-025-08189-5.

## Introduction

Parkinson’s disease (PD) is a progressive neurodegenerative disorder characterised by the loss of dopaminergic neuronal cells in the substantia nigra pars compacta (SNpc) and an abnormal accumulation of misfolded α-synuclein aggregates, called Lewy bodies (LBs), in surviving affected neurons [1]. The aetiology of PD in most patients is unknown, but many cases likely have a multifactorial onset resulting from the combined effect of genetic, behavioural, and environmental factors [2].

The main clinical manifestations can be grouped under the acronym TRAP: Tremor at rest, Rigidity, Akinesia (or bradykinesia), and Postural instability, although other features like flexed posture, freezing (motor blocks) and non-motor symptoms are usually present [3].

The estimated prevalence of PD in industrialised countries is 0.3% in the general population, 1.0% in people older than 60 years and 3.0% in people older than 80 years [4]. From a global perspective, it is projected that by 2050, 12 million people will be affected by PD, nearly double the current number, making PD one of the fastest-growing neurological disorder [5]. The physical, social, and emotional impact of PD is often significant, leading to compromised functional status, reduced quality of life, social isolation and increased burden on care partners [6].

Disease-modifying drugs are not yet available in clinical practice, therefore, current treatment for PD focuses on symptomatic relief through dopamine replacement therapy, with levodopa as the gold standard medication [7]. Despite the benefits it provides, treatment with levodopa causes several side effects, including nausea, dyskinesia, motor fluctuations, excessive daytime sleepiness, postural hypotension and hallucinations [8].

Several non-invasive brain stimulation (NIBS) techniques have been developed in recent decades to provide alternatives to pharmacological treatments. These techniques induce neuroplastic changes in the brain by modulating cortical activity, either enhancing or inhibiting it, with minimal side effects [9].

Transcranial Magnetic Stimulation (TMS) is one of the most widely used NIBS techniques. It induces changes in cerebral cortex excitability by generating a magnetic field through a wire coil. TMS can be administered as either single-pulse TMS (including paired-pulse TMS) or repetitive TMS (rTMS). Single-pulse and paired-pulse TMS are generally used to study brain functions, while rTMS is used to produce lasting changes in brain activity beyond the stimulation period [10]. In recent years, studies have begun exploring a new rTMS administration modality. This is known as Theta Burst Stimulation (TBS) and is characterized by the delivery of single bursts of rTMS at 50 Hz. The stimulation pattern mimics endogenous theta rhythms inducing long-term potentiation or depression of neuronal synaptic connections [11]. In preclinical studies, with a shorter application period than rTMS, TBS was shown to induce longer-lasting effects on synaptic plasticity [12].

Research has shown that rTMS offers therapeutic benefits for various neurological and psychiatric disorders. Specifically, high-frequency (HF) rTMS targeting the primary motor cortex (M1) on the side opposite to the pain site has an analgesic effect. Similarly, HF-rTMS applied to the dorsolateral prefrontal cortex (DLPFC) has been shown to alleviate depression, while low-frequency (LF) rTMS delivered to the contralesional M1 promotes motor recovery during the post-acute stage of stroke [13].

Although an increasing number of positive findings support the effectiveness of rTMS in PD, a definitive consensus has not yet been reached, as the results of existing studies are not entirely consistent. These discrepancies stem from variations in stimulation parameters and patient characteristics, which can significantly influence treatment outcomes. To assist clinicians and guide future research, we conducted this overview of systematic reviews with meta-analyses to consolidate current evidence on the effectiveness of TMS in treating motor symptoms of PD. Our aim was to map existing knowledge, identify remaining gaps, and address inconsistencies in reviews findings using a conceptual framework based on the methodology of a recent overview [14].

## Methods

### Study design

The present study was conducted in accordance with the Cochrane guidelines for overview of reviews [15] and adhered to the Preferred Reporting Items for Overviews of Reviews (PRIOR) guidelines of healthcare interventions [16, 17]. The review protocol was prospectively registered in the International Prospective Register of Systematic Reviews (PROSPERO) database (CRD42024539284, 5 May 2024).

### Eligibility criteria

Systematic reviews with meta-analyses (SRMAs) of randomized controlled trials (RCTs) were eligible for inclusion if they involved patients diagnosed with PD who presented motor impairments and underwent TMS interventions, including low-frequency, high-frequency, or theta-burst stimulation, studied either independently or comparatively. Additionally, inclusion required the presence of a control group in which patients received either a sham TMS treatment or a standard rehabilitation protocol.

To classify a publication as a ‘systematic review,’ all of the following methodological steps must have been conducted:

- a comprehensive and explicit search strategy;
- an assessment of the risk of bias in the primary studies;
- explicit methods for data extraction and quantitative synthesis of study findings (meta-analysis).

The primary outcome was general motor impairment and disability, as assessed using the Unified Parkinson’s Disease Rating Scale (UPDRS) part III. Secondary outcomes included: (1) freezing of gait (assessed with the Freezing of Gait Questionnaire [FOG-Q]); (2) functional mobility and balance (assessed with the Timed Up and Go Test [TUG]); and (3) walking time (WT) (assessed with the Six-Minute Walk Test [6MWT] and the 10-Metre Walk Test [10MWT]).

### Search strategy

A comprehensive search for records was carried out from inception to 7 May 2024 in the following electronic databases: MEDLINE, Cochrane Database of Systematic Reviews (CDSR), Epistemonikos, SCOPUS and Web of Science. The search was restricted to English-language publications. The search strategies for each database are detailed in Appendix A.1.

### Study screening and selection

Records retrieved were processed through The Deduplicator [18], a tool within the Systematic Review Accelerator suite [19] to remove duplicates before being uploaded to the Rayyan [20] platform for selection. Two independent researchers subsequently screened the records by reading the titles and abstracts and removed obviously irrelevant ones. The full texts of the remaining potentially eligible records were retrieved to determine final inclusion based on the inclusion and exclusion criteria. When necessary, a third author was consulted to resolve disagreements between reviewers at each phase of the selection process.

### Data extraction

Two independent researchers extracted data as reported in the original SRs using a predefined data extraction form. The extracted data included: characteristics of the SRs (first author; year of review publication, search time frame, conflict of interest, and sources of funding for the authors) and characteristics of the primary studies included in each review (number of included studies, total number participants, description of interventions and outcomes). For each meta-analysis (MA) examining outcomes relevant to this overview, we extracted the effect size (mean difference [MD] or standardized mean difference [SMD]), the associated confidence interval (CI), and the I² statistic as a measure of heterogeneity.

In cases of missing information, the corresponding authors of the SRs were contacted. Disagreements in the data collection process were resolved either through a consensus process or by consulting a third author.

### Review and trial quality assessment

Two review authors independently assessed the methodological quality of the included SRs using the AMSTAR 2 tool (A Measurement Tool to Assess Reviews) [21]. Information on the risk of bias (RoB) assessment of the primary studies was collected and presented as reported in the SRs.

### Overlap between primary studies

The primary studies included in each SR were compiled and cross-referenced in an evidence matrix table to assess the degree of overlap between reviews, using the ccaR package [22]. For each outcome, we examined the matrix and calculated the Corrected Covered Area (CCA) to classify the degree of overlap as follows: “slight” (CCA 0–5%), “moderate” (CCA 6–10%), “high” (CCA 11–15%), or “very high” (CCA > 15%) [23].

### Strategies for data synthesis and statistical analysis

As recommended by the Cochrane guidelines, data were extracted as reported in the included SRs, then reformatted and presented in text, tables, and figures without being reanalysed. To facilitate the comparison between the MAs of the different SRs, we converted the extracted results into a common effect size (MD for UPDRS-III, TUG, FOG-Q and SMD for WT) using the standard deviation of the control group from the most representative trial (the one with the highest number of participants) for each MA.

### Concordance or discordance of effect size

A conceptual framework was used to assess the concordance or discordance of effect sizes across MAs, specifically regarding the direction of the effects (favouring intervention, control, or no difference). Concordance was defined as the alignment of 80% or more of the MAs in the same direction. In instances of discordance between MAs (less than 80% of MAs indicating the same direction of effect), potential sources of heterogeneity were investigated. A sensitivity analysis was subsequently performed, excluding the following: (1) reviews rated as critically low quality according to the AMSTAR 2 tool; (2) reviews published before 2020; and (3) meta-analyses with a participant sample size of fewer than 200. The robustness of the evidence, in terms of the consistency of results across various sources of heterogeneity, was discussed in relation to the identified causes of discordance [24].

### Certainty of evidence

Two review authors independently assessed the Certainty of Evidence (CoE) using the GRADE approach, employing the algorithm from Pollock [25], which was specifically developed for Cochrane overviews of reviews. According to this algorithm, each review begins with a rating of high certainty, which may be downgraded by one level for any of four serious methodological concerns: (1) imprecision (i.e., a sample size of the meta-analysis < 200 participants); (2) risk of bias, at the trial level, due to the randomisation process in > 75% of the primary studies included in the meta-analysis; (3) inconsistency (high heterogeneity, defined as I² >75%); and (4) methodological quality, at the review level, in relation to selected critical items in AMSTAR 2.

## Results

### Systematic review and primary study selection

**Fig. 1:**
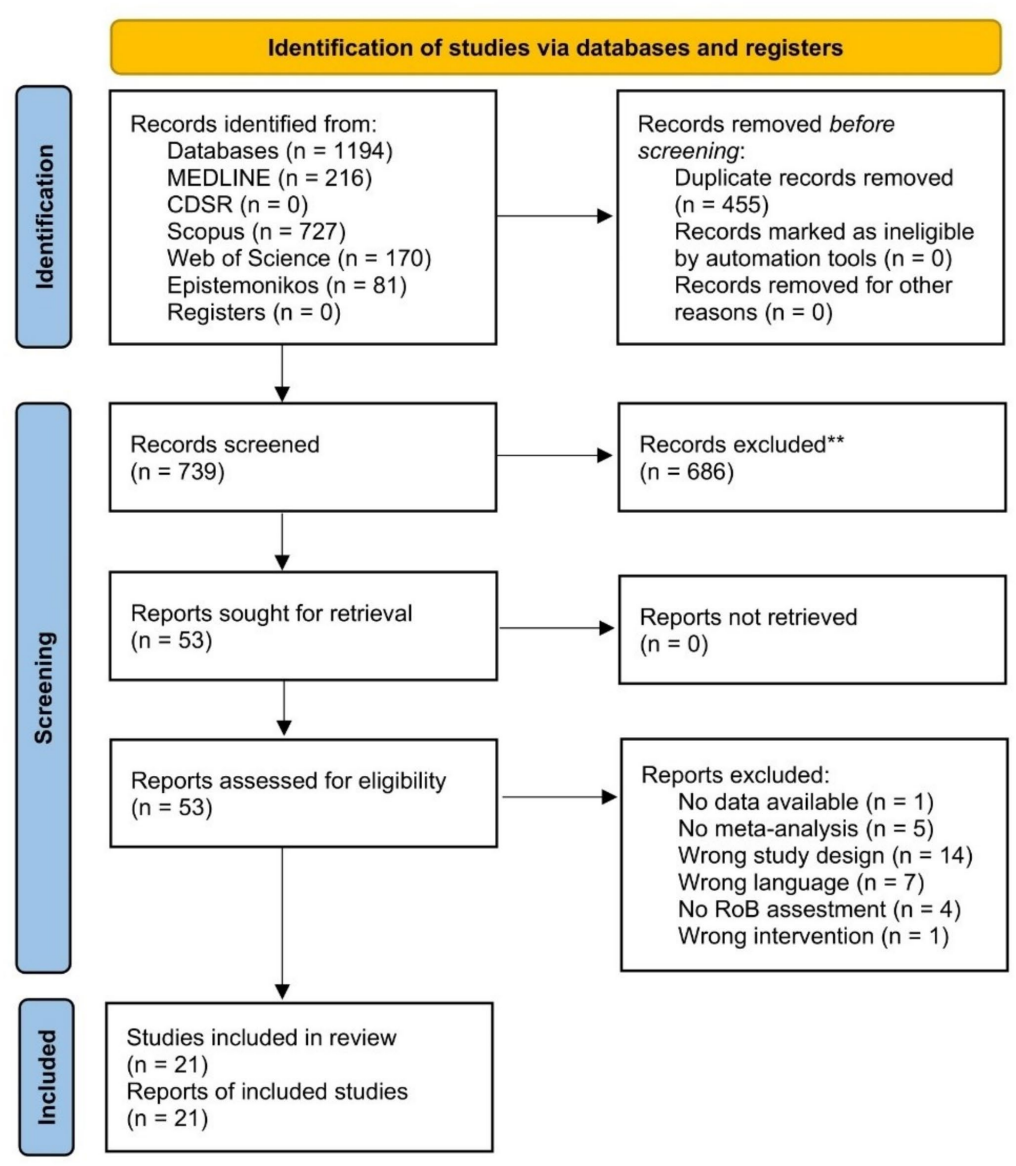
PRISMA flow diagram describing the study selection process

The search identified 1194 publications from databases. After removing duplicates, 739 records were screened, 686 of which were excluded after reviewing the title and abstract. The full texts of 53 reviews were retrieved for further detailed assessment, and ultimately, 21 reviews were included. The study flow chart is illustrated in Fig. 1, while the references of the reviews with the reason for inclusion or exclusion are presented in Appendix A.2.

### Description of reviews

A total of 21 reviews published between 2015 and 2024 with 107 unique primary studies were included. The number of studies per review ranged from 7 to 36, with a median of 16 per review, while the number of participants varied from 199 to 1122, with a median of 470 patients. More than half of the reviews (61.9%) were published in the past 5 years. Overall, none of the reviews declared a conflict of interest, and 13 (61.8%) reported a source of funding. The majority (71%) included both high- and low-frequency interventions, while 24% focused solely on high-frequency interventions, and only one review (5%) investigated low-frequency interventions. The primary outcome, the UPDRS-III, was investigated in 80% of the reviews. Secondary outcomes included the FOG-Q, examined in 28% of the reviews; the TUG test, investigated in 33% of the reviews; and the WT, studied in 23% of the reviews. The general characteristics of the reviews are summarised in Appendix A.3.

### Overlap of primary studies

A high overlap was found across all reviews (CCA: 14.9%). A very high overlap was observed for both primary and secondary outcomes: CCA UPDRS-III: 16.1%, CCA FOG-Q: 26.4%, CCA TUG: 19.6% and CCA WT: 20.6%. Appendix A.4 presents the results of the overlap analysis and the corresponding heatmap figures.

### Methodological quality of reviews

**Fig. 2:**
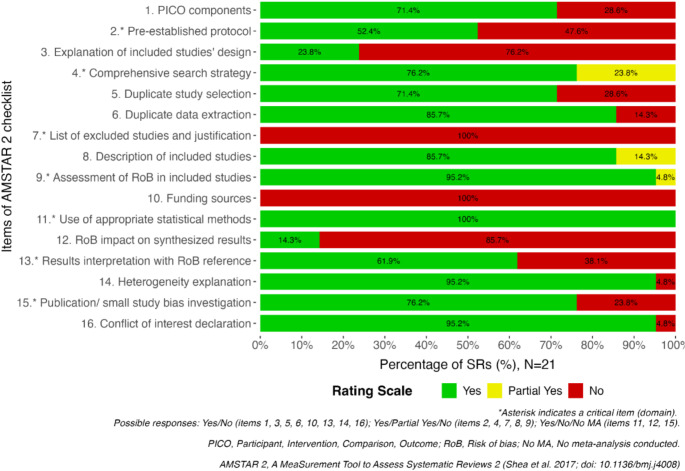
Summary plot reporting the AMSTAR 2 assessment for each domain across all systematic reviews

Of the 21 reviews, 4 (19%) were rated as low quality, and 17 (81%) were judged as critically low quality. Over half of the reviews had a pre-established protocol (52.4%) and 76.2% conducted a systematic literature search. However, 23.8% did not explain their selection of study designs, and 100% did not report a list of excluded studies. Nonetheless, the authors applied an adequate technique for assessing the RoB in almost all the reviews (95.2%). The results were interpreted while considering the RoB in 61.9% of the SRs, and 76.2% used appropriate methods for investigating publication bias. The summary plot of the AMSTAR 2 assessment is presented in Fig. 2, and the assessment of each review by each AMSTAR 2 domain is provided in Appendix A.5.

### Risk of bias assessment in primary studies

Six SRs (28%) employed the PEDro Scale to evaluate the RoB, while 16 SRs (76%) utilised the Cochrane Risk of Bias Tool. Some authors applied both instruments to assess the RoB. One SR was evaluated using an adapted, but not validated, version of the Concord Checklist. Assessment of RoB in primary studies is shown in Appendix A.6.

### Summary of results

In the following paragraph, the results will be presented divided by outcomes and subgroups, with the data converted to a common effect size as described in paragraph 2.8. A table reporting both the original effect sizes extracted from the reviews and their converted values is provided in Appendix A.7 and a table detailing the process to resolve discordances between reviews is provided in Appendix A.8.

#### Unified Parkinson’s disease rating scale– part III

**Fig. 3:**
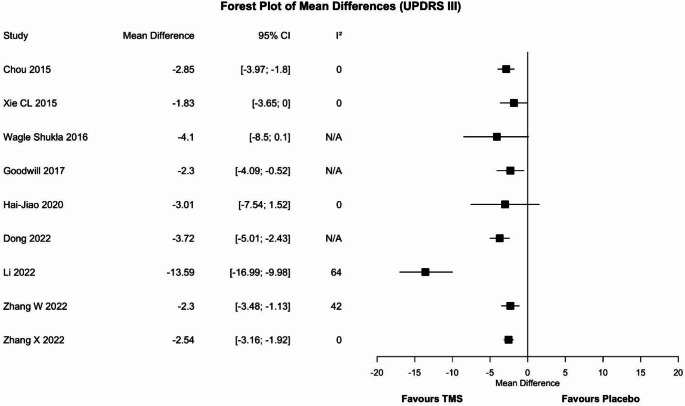
Forest plot of the mean difference for the UPDRS-III outcome. Values to the left of the non-difference line are favourable to TMS, those to the right are favourable to placebo. CI = confidence interval, I^2^ = heterogeneity

Nine meta-analyses [26–34] assessed the efficacy of rTMS without considering different modalities of administration (Fig. 3). Discordant results emerged: six meta-analyses (67%) reported the superiority of rTMS over placebo, whereas three found no difference between groups. According to a planned sensitivity analysis, the most updated and higher quality reviews (100%, *n* = 2) agreed on the superiority of rTMS. However, when considering only the meta-analysis involving the largest number of participants (75%, *n* = 8) discordant results were observed once again.

*UPDRS– III short-term* Complete agreement emerged: all five meta-analyses [26, 28, 35–37] (100%) reported the superiority of rTMS over placebo. Sensitivity analysis confirmed the direction of effects (Appendix A.9, Figure A.9.1).

*UPDRS– III long-term* Discordant results emerged: three meta-analyses [26, 35, 36] (60%) reported the superiority of rTMS over placebo, whereas two [28, 37] found no difference between groups. Furthermore, due to the lack of more recent and higher-quality reviews, the direction of the effect remains uncertain. (Appendix A.9, Figure A.9.2).

*UPDRS– III low-frequency* Discordant results emerged: six meta-analyses [31, 33, 35, 36, 38, 39] (67%) found no difference between groups, whereas three [28, 32, 39] reported the superiority of rTMS. Furthermore, with just one more updated and higher quality review, no significant differences can be identified between groups (Appendix A.9, Figure A.9.3).

*UPDRS– III high-frequency* Discordant results emerged: seven meta-analyses [31–33, 36, 39, 40] (78%) reported the superiority of rTMS over placebo, whereas two [28, 35] found no difference between groups. There is no significant consensus on the intervention; however, the more recent and higher-quality review reported the superiority of rTMS. (Appendix A.9, Figure A.9.4).

*UPDRS– III M1* Complete agreement emerged: all three meta-analyses [32, 35, 36] (100%) reported the superiority of rTMS over placebo. Sensitivity analysis confirmed the direction of the effect (Appendix A.9, Figure A.9.5).

*UPDRS– III DLPFC* Discordant results emerged: two meta-analyses [35, 36] (67%) found no difference between groups, whereas one [32] reported the superiority of rTMS over placebo. Furthermore, due to a lack of more updated and higher quality reviews, the direction of the effect remains uncertain. (Appendix A.9, Figure A.9.6).

*UPDRS– III SMA* Complete agreement emerged: all three meta-analyses [32, 35, 36] (100%) reported the superiority of rTMS over placebo. Sensitivity analysis confirmed the direction of the effect (Appendix A.9, Figure A.9.7).

*UPDRS– III M1 + DLPFC* The analysis is limited to a single meta-analysis [36] reporting no differences between interventions (Appendix A.9, Figure A.9.8).

*UPDRS– III LF-M1* Agreement among results emerged: four meta-analyses [26, 31, 39] (80%) reported no difference between groups. Sensitivity analysis confirmed the direction of the effect (Appendix A.9, Figure A.9.9).

*UPDRS– III LF-DLPFC* Discordant results emerged: one meta-analysis (50%) reported the superiority of rTMS over placebo, whereas one found no difference between groups. Furthermore, due to lack of more updated and higher quality reviews, the direction of the effect remains uncertain. (Appendix A.9, Figure A.9.10).

*UPDRS– III LF-SMA* Complete agreement emerged: two meta-analyses [31, 32] (100%) reported no difference between groups. Sensitivity analysis confirmed the direction of the effect (Appendix A.9, Figure A.9.11).

*UPDRS– III LF-OFR* The analysis is limited to a single meta-analysis [26] reporting the superiority of rTMS over placebo (Appendix A.9, Figure A.9.12).

*UPDRS– III HF-M1* Agreement among results emerged: five meta-analyses [26, 31, 32, 36, 39] (83%) reported the superiority of rTMS over placebo, whereas one [39] found no difference between groups. Sensitivity analysis confirmed the direction of the effect (Appendix A.9, Figure A.9.13).

*UPDRS– III HF-DLPFC* Discordant results emerged: three meta-analyses [31, 36, 39] (60%) found no difference between groups, while one [32] reported the superiority of rTMS over placebo and one [39] reported the superiority of placebo over rTMS. There is a more recent and higher quality review reporting no difference between interventions. (Appendix A.9, Figure A.9.14).

*UPDRS– III HF-SMA* Discordant results emerged: three meta-analyses [31, 32, 39] (60%) reported the superiority of rTMS over placebo, whereas two [36, 39] found no difference between groups. There is only one review that is more recent and of higher quality reporting the superiority of rTMS (Appendix A.9, Figure A.9.15).

*UPDRS– III HF-M1 + DLPFC* Discordant results emerged: two meta-analyses [31, 39] (50%) reported the superiority of rTMS over placebo, whereas two [36, 39] found no difference between groups. There is only one review that is more recent and of higher quality reporting the superiority of rTMS (Appendix A.9, Figure A.9.16).

*UPDRS– III HF-OFR* The analysis is limited to a single meta-analysis [26] which found no difference between groups. (Appendix A.9, Figure A.9.17).

*UPDRS– III HF-PMD* The analysis is limited to a single meta-analysis [36] reporting no difference between groups (Appendix A.9, Figure A.9.18).

*UPDRS– III ON state* Discordant results emerged: seven meta-analyses [26, 32, 35, 36, 41] (70%) reported the superiority of rTMS over placebo, whereas three [41, 42] found no difference between groups. Sensitivity analysis did not confirm the consistency of the results due to the absence of more recent and higher quality reviews and discordance of meta-analyses with populations > 200 individuals. The direction of the effect remains uncertain. (Appendix A.9, Figure A.9.19).

*UPDRS– III OFF state* Discordant results emerged: six meta-analyses [26, 33, 35, 36, 41, 42] (60%) reported the superiority of rTMS over placebo, whereas four [32, 41] found no difference between groups. Sensitivity analysis did not confirm the consistency of the results due to the absence of more recent and higher quality reviews and discordance of meta-analyses with populations > 200 individuals, it also showed a picture of uncertainty regarding the intervention (Appendix A.9, Figure A.9.20).

*UPDRS– III single session* Complete agreement emerged: all three meta-analyses [32, 35, 36] (100%) reported no difference between groups. Sensitivity analysis confirmed the direction of the effect (Appendix A.9, Figure A.9.21).

*UPDRS– III multiple session* Complete agreement emerged: all three meta-analyses [32, 35, 36] (100%) reported the superiority of rTMS over placebo. Sensitivity analysis confirmed the direction of the effect (Appendix A.9, Figure A.9.22).

#### Freezing of gait– questionnaire

**Fig. 4:**
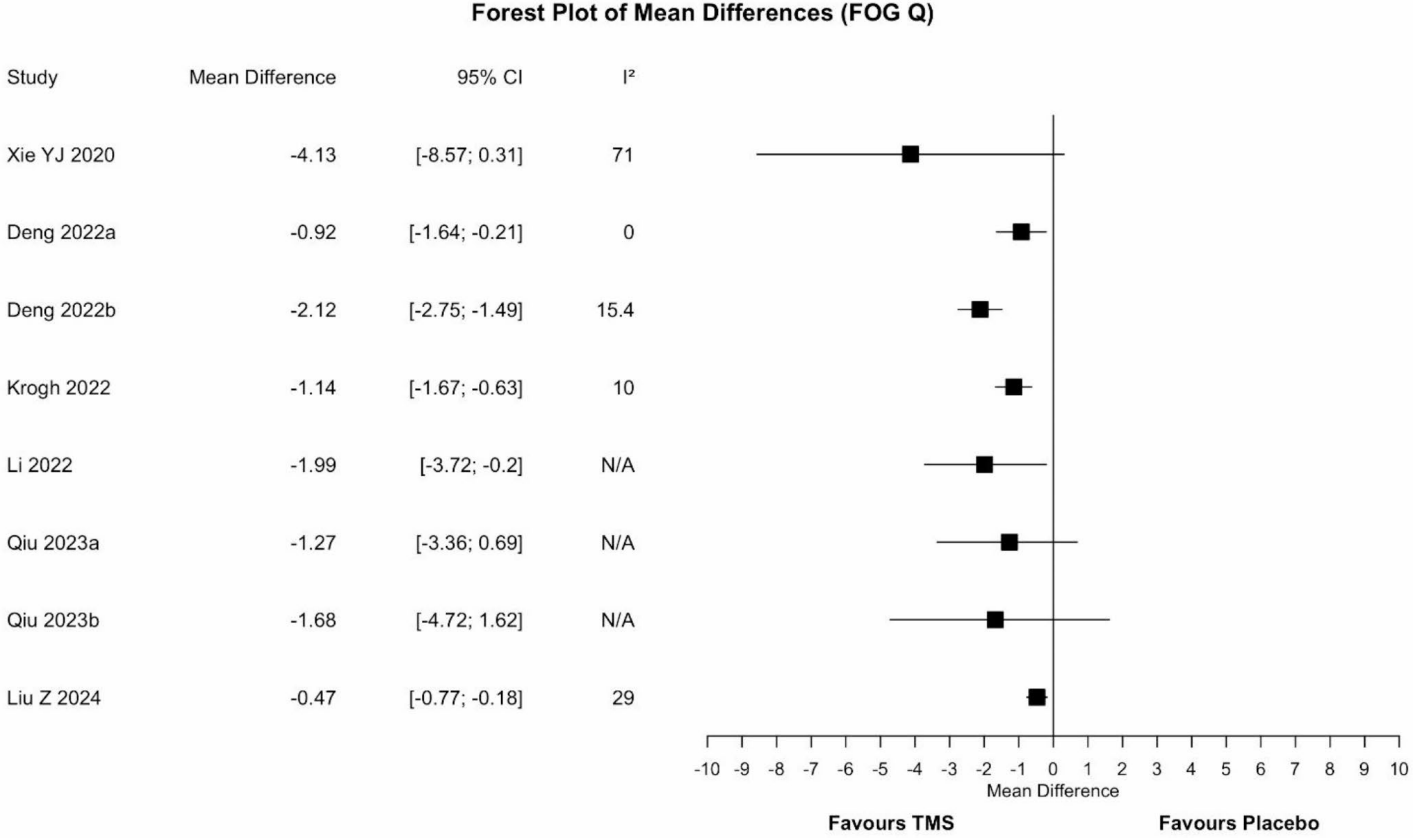
Forest plot of the mean difference for the freezing Of gait Questionnaire outcome. Values to the left of the non-difference line are favourable to TMS, those to the right are favourable to placebo. CI = confidence interval, I^2^ = heterogeneity

Eight meta-analyses assessed the efficacy of rTMS (Fig. 4). Discordant results emerged: five meta-analyses [32, 43–45] (63%) reported the superiority of rTMS over placebo, whereas three [39, 46] found no difference between groups. According to a planned sensitivity analysis, the most updated and higher quality reviews (100%, *n* = 2) agreed on the superiority of rTMS. However, when considering only the meta-analysis involving the largest number of participants (63%, *n* = 8) discordant results were observed once again.

#### Time up and go

**Fig. 5:**
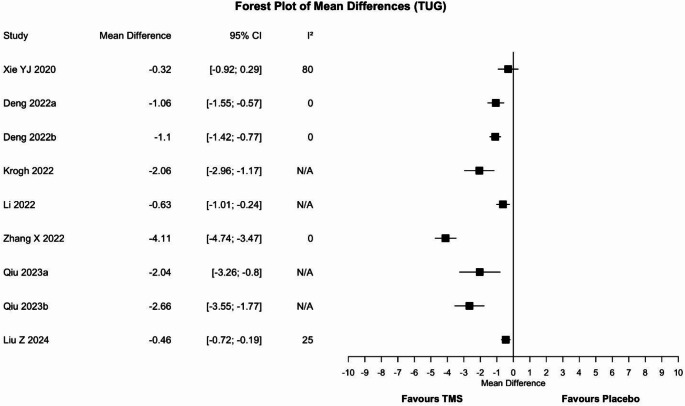
Forest plot of the mean difference for the timed up and go test. Values to the left of the non-difference line are favourable to TMS, those to the right are favourable to placebo. CI = confidence interval, I^2^ = heterogeneity

Nine meta-analyses assessed the efficacy of rTMS (Fig. 5). Agreement among results emerged: eight meta-analyses [32, 34, 39, 43–45] (89%) reported the superiority of rTMS over placebo, whereas one [46] found no difference between groups. Sensitivity analysis confirmed the superiority of rTMS.

#### Walking time

**Fig. 6:**
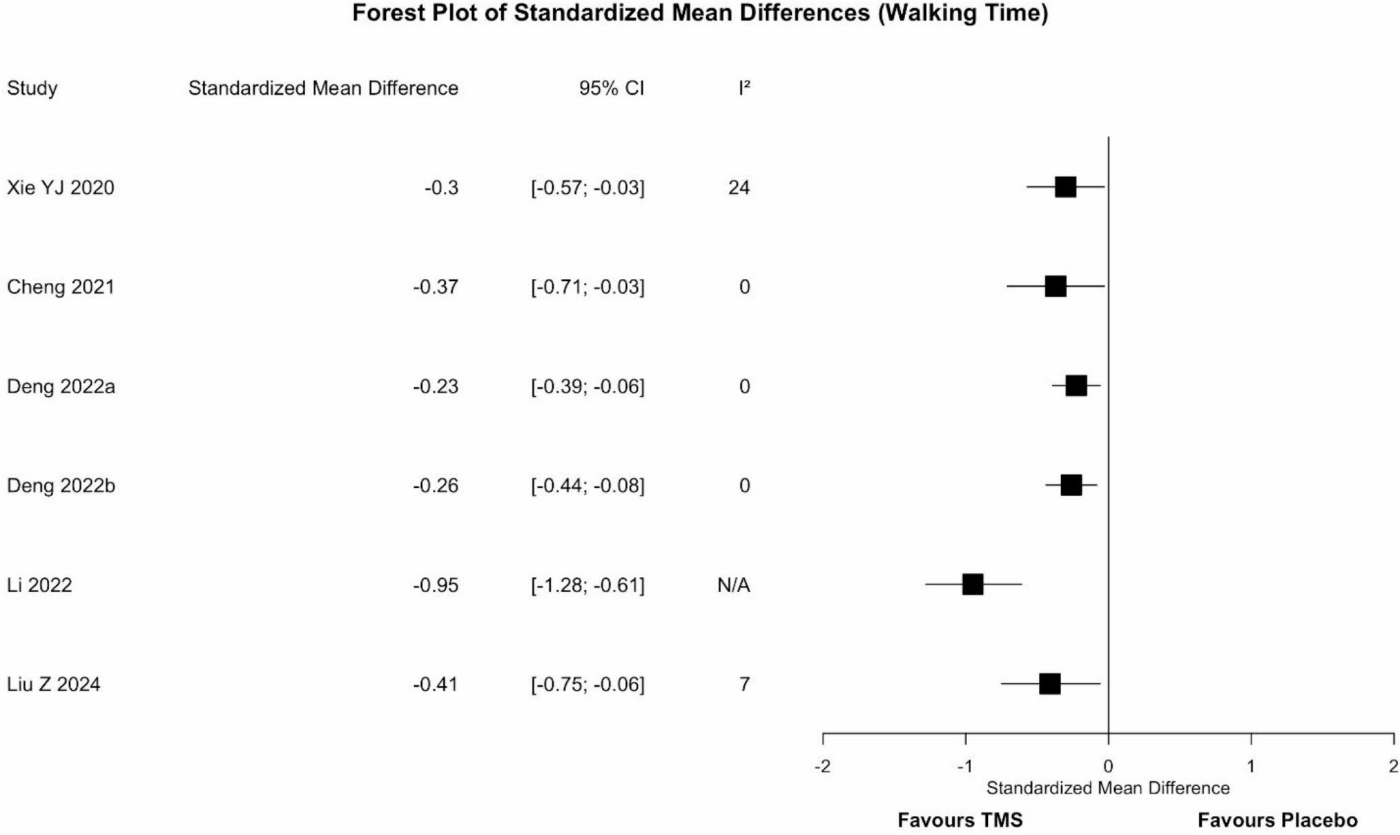
Forest plot of the standardized mean difference for the walking time outcome. Values to the left of the non-difference line are favourable to TMS, those to the right are favourable to placebo. CI = confidence interval, I^2^ = heterogeneity

Six meta-analyses [32, 42, 43, 45, 46] assessed the efficacy of rTMS (Fig. 6). Agreement among results emerged: all six meta-analyses (100%) reported the superiority of rTMS over placebo. Sensitivity analysis confirmed the direction of the effect.

### Certainty of evidence

Of the RS, 8 were rated with a moderate CoE, and 13 with low CoE. Reasons for downgrading were mainly due to serious (*n* = 4) and very serious (*n* = 17) limitations of methodological quality of RS’s, inconsistency (I^2^ > 75%) (*n* = 9), and high RoB at the RTC level (*n* = 8). Most meta-analyses (*n* = 19) involved more than 200 participants. (Appendix A.10).

### Adverse effects

Few reviews (*n* = 10, 47%) reported adverse effects (AEs) of treatment. Two reviews did not observe or report any AEs, while eight reported mild events such as transient headache, neck pain, local burning sensation, increased salivation, tinnitus, vertigo, and nausea (Appendix A.11).

## Discussion

This overview summarises the evidence from 21 SRMAs, including 107 unique primary studies, on the use of TMS for the improvement of motor symptoms in PD.

All the primary studies included in the SRs used repetitive TMS protocols, as this is the modality employed to achieve therapeutic outcomes, whereas the single-pulse modality is primarily used in studies investigating brain functions.

Regarding the primary outcome, the general motor impairment, which was measured through the UPDRS scale part III, it can be observed that the results of MAs that do not differentiate between various stimulation modalities and sites, despite a very high quantified overlap (16.1%), do not fully align.

In particular, 67% of the SRs agree about the superiority of rTMS compared to the control group, while three SRs report non-statistically significant results.

To attempt to explain this heterogeneity in the results, it is important to first highlight that the results of the three discordant MAs are only narrowly non-statistically significant, while the point estimate and the upper bound of the confidence interval fall well within the range favouring the superiority of rTMS.

Upon a detailed analysis of the studies included in these MAs, two SRs [27, 30] included in the analysis only primary studies that administered rTMS to the DLPFC region. As we will further discuss in the subgroup analysis, the DLPFC area does not appear to be the optimal stimulation site for achieving significant improvements in motor symptoms, but it may be more suitable for treating non-motor symptoms such as depression [13].

All three discordant SRs have a very low rating in the AMSTAR 2 assessment and a low CoE according to the GRADE criteria, whereas four of the six systematic reviews supporting the superiority of rTMS demonstrate moderate CoE, thereby lending greater weight to their findings.

The sensitivity analysis, which considers only the most recent and highest-quality SRs, further confirms the superiority of rTMS, with two reviews [31, 34] having a moderate overlap of primary studies (6.1%), both showing moderate CoE, that agree on the direction of the effect.

To assess the clinical relevance of the effect size observed in these findings, a minimal clinically important difference (MCID) of 5 points on the UPDRS-III scale can be used [47], along with the framework proposed by Man-Son-Hing et al. for its interpretation [48].

The study by Dong et al. [31] appears to suggest a possible clinically relevant result, while the study by Zhang et al. [34] seems to indicate a result that is not clinically relevant. It is important to consider that these results combine studies with different parameters and stimulation sites, which, as we will see in the subgroup analysis, are relevant factors that can significantly influence the outcome of the stimulation. Variability in the included population may also account for some of the observed differences, as the physiological and clinical effects of TMS depend not only on the stimulation parameters but also on how the brain receives and processes the stimulation. These effects are influenced by the excitability state of the targeted cortical region and the structural and functional connectivity within the targeted network [49].

Specifically, when looking at the stimulation modalities, the two parameters that appear to have the greatest influence on the treatment effect are frequency (high or low) and stimulation site. After resolving the discrepancies and conducting the sensitivity analysis, it seems clear that high frequency (> 1 Hz) and the M1 site are the parameters that ensure the greatest treatment efficacy, with nearly all reviews concordant.

Considering now the clinical relevance of high-frequency rTMS administered at the M1 site, four [26, 32, 36, 39] out of the five meta-analyses supporting rTMS show a possible clinically relevant result.

We can therefore state that we are fairly certain that rTMS administered with these parameters can induce improvements in patients’ motor symptoms. However, the clinical relevance of these improvements is not yet entirely clear.

In assessing the clinical relevance, it is important to consider the very rare and mild adverse effects associated with rTMS. This perspective allows us to assert that, even if the magnitude of the effect is moderate rather than substantial, the treatment can undoubtedly represent an excellent alternative or, at the very least, a valuable adjunct to pharmacological therapy, which, on the other hand, is often associated with various potentially significant side effects.

Considering rTMS as part of a comprehensive patient management approach rather than a standalone therapy, it can be hypothesized that the improvements achieved through its administration might predispose patients to engage more actively and participatively in rehabilitation sessions, enhancing the overall outcomes of patient care.

Examining the reasons why high-frequency stimulation appears to be superior to low-frequency stimulation in improving motor symptoms in PD patients, it is important to note the widely accepted understanding that high-frequency stimulation seems to exert excitatory effects, whereas low-frequency stimulation is generally associated with inhibitory effects [50].

With this in mind, considering that the motor manifestations of PD are primarily caused by an inhibitory effect on the movement pathways resulting from the depletion of the SNpc, it seems reasonable to suggest that a therapy that stimulates these circuits (HF-rTMS) could lead to better outcomes compared to a therapy that further inhibits them (LF-rTMS).

The reason why stimulation of the motor areas (both primary and supplementary) appears to produce more effective results compared to other brain regions can be attributed to the fact that targeting regions directly involved in movement is likely to have a greater impact on improving motor-related outcomes. This underscores the importance of ensuring that stimulation is precisely focused on specific regions, depending on the intended therapeutic goal (for example, the DLPFC for depression, as previously mentioned).

To achieve this level of precision, an important technical parameter is the coil used for stimulation. Despite considerable variability, coils can generally be categorized into two main types: circular (C) coils and figure-8 (F8) coils, with the latter being more focal, offering greater penetration and efficacy [51].

Among the included SRs, only two of them [38, 41], with CoE ranging from low to moderate, conducted a specific subgroup analysis to evaluate the differences in efficacy between F8 and C coils. Both results showed that the pooled effect of studies using F8 coils was superior to those using C coils.

It seems therefore plausible that the use of F8 coils may yield better results compared to C coils; however, additional studies specifically designed to assess this comparison are required to draw definitive conclusions.

Another parameter that has been examined in only a few reviews, but could significantly impact results and partially explain the discordance between them, is the difference between unilateral and bilateral stimulation of M1.

In the three SRs [31, 33, 36] that assessed the difference between these two modalities, all reported that bilateral stimulation produces better results than unilateral stimulation. The strength of the evidence presented in these reviews was consistently rated as moderate. However, further studies focusing on this specific parameter are required to enable definitive recommendations for clinical practice.

Another important parameter assessed in the subgroup analysis is the number of sessions provided. With low to moderate CoE, the majority of systematic reviews agree that multiple sessions are superior to single sessions. Given that the proposed primary mechanism of action of TMS is the long-term potentiation (LTP), which relies on synaptic plasticity induced by repetitive and persistent stimulation, it is reasonable to expect that multiple sessions of rTMS would enhance this mechanism compared to a single session [52]. A similar mechanism could also be the explanation of why three SRs [26, 36, 41] reported that an elevated number of pulses per session is associated with better results compared to a lower number of pulses.

Although there is general agreement on the positive relationship between a greater volume of stimulation (in terms of the number of sessions and pulses) and improvements in motor symptoms, it is important to note that the CoE supporting this relationship is predominantly low. Additionally, further investigations are needed to determine whether a higher volume of stimulation increases the risk of AEs.

It is important to highlight that, while the available evidence confirms the short-term effects of rTMS, there is considerable uncertainty about the persistence of these effects in the long term. Given that the persistence of treatment effects over time is a key factor, particularly in reducing reliance on pharmacological therapy, a greater number of longitudinal clinical studies should include long-term follow-ups. This will allow a more consistent evaluation of treatment effect duration in future reviews.

Uncertainty also remains regarding the influence of medication state (ON vs. OFF) on the observed effects.

Regarding patient characteristics and their impact on treatment efficacy, unfortunately, it is not possible to draw substantial conclusions from the currently available literature, as only one systematic review has attempted to evaluate this aspect. Specifically, the study by Goodwill et al. [29] conducted a subgroup analysis based on disease severity, categorizing it as mild, moderate, or severe according to the Hoehn & Yahr (H&Y) scale, and found no significant differences in treatment effect. Furthermore, many reviews did not report data on specific patient characteristics. Even when such data were available, this information was missing in some primary studies, preventing the calculation of a mean value across all included studies in those SRs Future research should therefore focus on determining the impact of various patient characteristics (such as age, disease duration, etc.) on the effectiveness of TMS treatment. This would facilitate the identification of patient subgroups most likely to benefit from this therapy, enabling clinicians to tailor TMS prescriptions to the most suitable patients.

Regarding the secondary outcomes, despite minor discrepancies, the higher-quality and more recent SRs identified through the sensitivity analysis consistently report positive effects of rTMS, with 100% of the SRs agreeing on its benefits for gait improvement. Moreover, these SRs, which confirm the direction of the effect, contain a high proportion of unique primary studies, minimizing the risk of result duplication that could have arisen from the high overlap estimated when considering all the reviews. Unfortunately, no subgroup analyses were conducted to assess the effects of different protocols, which could potentially explain the small variability observed across the results of the SRs. Nevertheless, it can be hypothesized that the parameters analysed for the primary outcome may also influence these secondary outcomes. However, based on the available evidence, this cannot be conclusively established.

Despite our attempt to draw conclusions based on the highest-quality SRs through sensitivity analysis, it is important to highlight that, overall, the included SRs were rated as having low or very low quality. This is reflected in the CoE for each outcome, which never exceeds a “moderate” level. Therefore, it is crucial for future reviews to give closer attention to specific methodological aspects, in particular: providing a list of excluded studies with justifications, disclosing funding sources, considering the impact of RoB assessment in the synthesis of results, and offering a detailed explanation of the inclusion criteria for study designs. Addressing these aspects will enhance the quality of future reviews and improve the certainty of their findings.

## Conclusions

In conclusion, based on the most up-to-date, comprehensive, and high-quality SRMAs currently available, HF-rTMS targeting motor areas (both primary and supplementary) has been shown to produce, with a low incidence of mild side effects, positive improvements in general motor impairment, gait, functional mobility, and balance in patients with PD. Various stimulation parameters, such as the number of sessions, number of pulses, and coil type influence the magnitude of these effects. Further research is needed to investigate these factors more thoroughly and to establish optimal protocols that maximize positive outcomes and their long-term maintenance while minimizing adverse effects.

Furthermore, it is important to emphasize that lifestyle factors, such as diet, physical activity, and sleep quality, significantly influence neurophysiological responses to TMS. Thus, a comprehensive, multidisciplinary and personalized approach is always essential to optimize the clinical care of those patients.

## Electronic supplementary material

Below is the link to the electronic supplementary material.

Supplementary Material 1
