# Supplementary material for: The effects of transcranial magnetic stimulation in motor symptoms of Parkinson’s disease: an overview of systematic reviews with meta-analysis
Source: Neurol Sci. 2025 Apr 16;46(8):3405–18. doi: 10.1007/s10072-025-08189-5 (PMC12267310; doi:10.1007/s10072-025-08189-5)
Supplement: Supplementary file 1 — Supplementary Material 1 [file 10072_2025_8189_MOESM1_ESM.docx]

**APPENDIX A: SUPPLEMENTARY MATERIAL**

[**APPENDIX A.1. Methods** 3](#_Toc188093381)

[Table A.1.1 List of key terms and their synonyms 3](#_Toc188093382)

[Search strategies 3](#_Toc188093383)

[**APPENDIX A.2. Eligibility** 6](#_Toc188093384)

[Table A.2.1 References of included and excluded SRs 6](#_Toc188093385)

[**APPENDIX A.3. Characteristics of the included SRs** 10](#_Toc188093386)

[Table A.3.1 General characteristics for each SRs 10](#_Toc188093387)

[**APPENDIX A.4. Overlapping of primary studies included in SRs** 12](#_Toc188093388)

[Figure A.4.1 Overlapping SRs of primary studies for all studies 12](#_Toc188093389)

[Table A.4.1 Overlapping SRs of primary studies for all studies 12](#_Toc188093390)

[Figure A.4.2 Overlapping SRs of primary studies for Unified Parkinson’s Disease Rating Scale – Part III outcome 13](#_Toc188093391)

[Table A.4.2 Overlapping SRs of primary studies for Unified Parkinson’s Disease Rating Scale – Part III outcome 13](#_Toc188093392)

[Figure A.4.3 Overlapping SRs of primary studies for Freezing of Gait outcome 14](#_Toc188093393)

[Table A.4.3 Overlapping SRs of primary studies for Freezing of Gait outcome 14](#_Toc188093394)

[Figure A.4.4 Overlapping SRs of primary studies for Time Up and Go outcome 14](#_Toc188093395)

[Table A.4.4 Overlapping SRs of primary studies for Time Up and Go outcome 15](#_Toc188093396)

[Figure A.4.5 Overlapping SRs of primary studies for Walking Time outcome 15](#_Toc188093397)

[Table A.4.5 Overlapping SRs of primary studies for Walking Time outcome 15](#_Toc188093398)

[**APPENDIX A.5. Review quality assessment AMSTAR 2** 16](#_Toc188093399)

[Table A.5.1 AMSTAR 2 assessment for each review 16](#_Toc188093400)

[**APPENDIX A.6. Risk of bias assessment in primary studies included in SRs** 17](#_Toc188093401)

[Table A.6.1 Risk of bias in primary studies 17](#_Toc188093402)

[**APPENDIX A.7. Strategy for data synthesis and statistical analysis** 18](#_Toc188093403)

[Table A.7.1 Mean Difference and Standardized Mean Difference conversion of main outcomes 18](#_Toc188093404)

[Table A.7.2 Mean Difference and Standardized Mean Difference conversion of UPDRS – III Subgroups 18](#_Toc188093405)

[**APPENDIX A.8. Strategies to resolve discordances** 20](#_Toc188093406)

[Table A.8.1 Main outcomes 20](#_Toc188093407)

[Table A.8.2 UPDRS – III Subgroups 21](#_Toc188093408)

[**APPENDIX A.9. Summary of evidence of included SRs on UPDRS – III subgroups** 26](#_Toc188093409)

[Figure A.9.1 Summary of evidence of included SRs on UPDRS – III: Short term 26](#_Toc188093410)

[Figure A.9.2 Summary of evidence of included SRs on UPDRS – III: Long term 26](#_Toc188093411)

[Figure A.9.3 Summary of evidence of included SRs on UPDRS – III: Low frequency 27](#_Toc188093412)

[Figure A.9.4 Summary of evidence of included SRs on UPDRS – III: High frequency 27](#_Toc188093413)

[Figure A.9.5 Summary of evidence of included SRs on UPDRS – III: M1 28](#_Toc188093414)

[Figure A.9.6 Summary of evidence of included SRs on UPDRS – III: DLPFC 28](#_Toc188093415)

[Figure A.9.7 Summary of evidence of included SRs on UPDRS – III: SMA 29](#_Toc188093416)

[Figure A.9.8 Summary of evidence of included SRs on UPDRS – III: M1+DLPFC 29](#_Toc188093417)

[Figure A.9.9 Summary of evidence of included SRs on UPDRS – III: LF-M1 30](#_Toc188093418)

[Figure A.9.10 Summary of evidence of included SRs on UPDRS – III: LF-DLPFC 30](#_Toc188093419)

[Figure A.9.11 Summary of evidence of included SRs on UPDRS – III: LF-SMA 31](#_Toc188093420)

[Figure A.9.12 Summary of evidence of included SRs on UPDRS – III: LF-OFR 31](#_Toc188093421)

[Figure A.9.13 Summary of evidence of included SRs on UPDRS – III: HF-M1 32](#_Toc188093422)

[Figure A.9.14 Summary of evidence of included SRs on UPDRS – III: HF-DLPFC 32](#_Toc188093423)

[Figure A.9.15 Summary of evidence of included SRs on UPDRS – III: HF-SMA 33](#_Toc188093424)

[Figure A.9.16 Summary of evidence of included SRs on UPDRS – III: HF-M1+DLPFC 33](#_Toc188093425)

[Figure A.9.17 Summary of evidence of included SRs on UPDRS – III: HF-OFR 34](#_Toc188093426)

[Figure A.9.18 Summary of evidence of included SRs on UPDRS – III: HF-PMD 34](#_Toc188093427)

[Figure A.9.19 Summary of evidence of included SRs on UPDRS – III: On-state 35](#_Toc188093428)

[Figure A.9.20 Summary of evidence of included SRs on UPDRS – III: Off-state 35](#_Toc188093429)

[Figure A.9.21 Summary of evidence of included SRs on UPDRS – III: Single session 36](#_Toc188093430)

[Figure A.9.22 Summary of evidence of included SRs on UPDRS – III: Multiple sessions 36](#_Toc188093431)

[**APPENDIX A.10. Certainty of evidence in meta-analysis** 37](#_Toc188093432)

[Table A.10.1 GRADE 37](#_Toc188093433)

[**APPENDIX A.11. Adverse events** 38](#_Toc188093434)

[Table A.11.1 Summary of reported adverse events 38](#_Toc188093435)

# **APPENDIX A.1. Methods**

## Table A.1.1 List of key terms and their synonyms

| PICOS | Key term | MeSH | Synonyms |
| --- | --- | --- | --- |
| P | Parkinson Disease | Parkinson Disease | Parkinson  Parkinson Disease  Parkinson’s Disease  Idiopathic Parkinson's Disease  Lewy Body Parkinson's Disease  Parkinson’s Disease, Idiopathic  Parkinson’s Disease, Lewy Body  Parkinson Disease, Idiopathic  Idiopathic Parkinson Disease  Lewy Body Parkinson Disease  Primary Parkinsonism  Parkinsonism, Primary  Paralysis Agitans |
| I | Transcranial Magnetic Stimulation | Transcranial Magnetic Stimulation | Magnetic Stimulation, Transcranial  Stimulation, Transcranial Magnetic  Transcranial Magnetic Stimulations  Transcranial Magnetic Stimulation, Single Pulse  Transcranial Magnetic Stimulation, Paired Pulse  Transcranial Magnetic Stimulation, Repetitive |
| C | - | - | - |
| O | Motor Symptoms | Motor disorders | - |
| S | Systematic review with meta-analysis | Review Literature as Topic  Systematic Review as Topic  Meta-Analysis as Topic | State-of-the-Art Review  State of the Art Review  State-of-the-Art Reviews  Review, State-of-the-Art  Reviews, State-of-the-Art  State of the Art Reviews  Systematic Review as Topic  Meta Analysis as Topic  Data Pooling  Data Poolings  Clinical Trial Overviews |

## Search strategies

MEDLINE SEARCH STRATEGY (via PubMed)

| #1 | ((((((((((((("Parkinson Disease"[MeSH Terms]) OR ("Parkinson Disease"[Title/Abstract])) OR ("Parkinson's Disease"[Title/Abstract])) OR (Parkinson[Title/Abstract])) OR ("Idiopathic Parkinson's Disease"[Title/Abstract])) OR ("Lewy Body Parkinson's Disease"[Title/Abstract])) OR ("Parkinson's Disease, Idiopathic"[Title/Abstract])) OR ("Parkinson's Disease, Lewy Body"[Title/Abstract])) OR ("Parkinson Disease, Idiopathic"[Title/Abstract])) OR ("Idiopathic Parkinson Disease"[Title/Abstract])) OR ("Lewy Body Parkinson Disease"[Title/Abstract])) OR ("Primary Parkinsonism"[Title/Abstract])) OR ("Parkinsonism, Primary"[Title/Abstract])) OR ("Paralysis Agitans"[Title/Abstract]) |
| --- | --- |
| #2 | ((((((("Transcranial Magnetic Stimulation"[MeSH Terms]) OR ("Transcranial Magnetic Stimulation"[Title/Abstract])) OR ("Magnetic Stimulation, Transcranial"[Title/Abstract])) OR ("Stimulation, Transcranial Magnetic"[Title/Abstract])) OR ("Transcranial Magnetic Stimulations"[Title/Abstract])) OR ("Transcranial Magnetic Stimulation, Single Pulse"[Title/Abstract])) OR ("Transcranial Magnetic Stimulation, Paired Pulse"[Title/Abstract])) OR ("Transcranial Magnetic Stimulation, Repetitive"[Title/Abstract]) |
| #3 | ((((((((((((((((((("Review Literature as Topic"[MeSH Terms]) OR ("Systematic Review as Topic"[MeSH Terms])) OR ("Meta-Analysis as Topic"[MeSH Terms])) OR ("Review Literature as Topic"[Title/Abstract])) OR ("Systematic Review as Topic"[Title/Abstract])) OR ("Meta-Analysis as Topic"[Title/Abstract])) OR ("State-of-the-Art Review"[Title/Abstract])) OR ("State of the Art Review"[Title/Abstract])) OR ("State-of-the-Art Reviews"[Title/Abstract])) OR ("Review, State-of-the-Art"[Title/Abstract])) OR ("Reviews, State-of-the-Art"[Title/Abstract])) OR ("State of the Art Reviews"[Title/Abstract])) OR ("Data pooling"[Title/Abstract])) OR ("Data Poolings"[Title/Abstract])) OR ("Clinical Trial Overviews"[Title/Abstract])) OR ("Overview, Clinical Trial"[Title/Abstract])) OR ("Literature Review"[Title/Abstract])) OR ("Systematic Review"[Title/Abstract])) OR ("Review"[Title/Abstract])) OR ("Meta-Analysis"[Title/Abstract]) |
| #4 | #1 AND #2 AND #3 |

EPISTEMONIKOS SEARCH STRATEGY

| 1 | (title:("Parkinson Disease") OR abstract:("Parkinson Disease")) OR (title:("Parkinson's Disease") OR abstract:("Parkinson's Disease")) OR (title:(Parkinson) OR abstract:(Parkinson)) OR (title:("Idiopathic Parkinson's Disease") OR abstract:("Idiopathic Parkinson's Disease")) OR (title:("Parkinson's DIsease Idiopathic") OR abstract:("Parkinson's DIsease Idiopathic")) OR (title:("Parkinson Disease, Idiopathic") OR abstract:("Parkinson Disease, Idiopathic")) OR (title:("Idiopathic Parkinson Disease") OR abstract:("Idiopathic Parkinson Disease")) OR (title:("Lewy Body Parkinson's Disease") OR abstract:("Lewy Body Parkinson's Disease")) OR (title:("Parkinson's Disease, Lewy Body") OR abstract:("Parkinson's Disease, Lewy Body")) OR (title:("Lewy Body Parkinson Disease") OR abstract:("Lewy Body Parkinson Disease")) OR (title:("Primary Parkinsonism") OR abstract:("Primary Parkinsonism")) OR (title:("Parkinsonism, Primary") OR abstract:("Parkinsonism, Primary")) OR (title:("Paralysis Agitans") OR abstract:("Paralysis Agitans")) |
| --- | --- |
| 2 | (title:("Transcranial Magnetic Stimulation") OR abstract:("Transcranial Magnetic Stimulation")) OR (title:("Magnetic Stimulation, Transcranial") OR abstract:("Magnetic Stimulation, Transcranial")) OR (title:("Stimulation, Transcranial Magnetic") OR abstract:("Stimulation, Transcranial Magnetic")) OR (title:("Transcranial Magnetic Stimulations") OR abstract:("Transcranial Magnetic Stimulations")) OR (title:("Transcranial Magnetic Stimulation, Single Pulse") OR abstract:("Transcranial Magnetic Stimulation, Single Pulse")) OR (title:("Transcranial Magnetic Stimulation, Paired Pulse") OR abstract:("Transcranial Magnetic Stimulation, Paired Pulse")) OR (title:("Transcranial Magnetic Stimulation, Repetitive") OR abstract:("Transcranial Magnetic Stimulation, Repetitive")) |
| 3 | (title:("Review Literature as Topic") OR abstract:("Review Literature as Topic")) OR (title:("Systematic Review as Topic") OR abstract:("Systematic Review as Topic")) OR (title:("Meta-Analysis as Topic") OR abstract:("Meta-Analysis as Topic")) OR (title:("State-of-the-Art Review") OR abstract:("State-of-the-Art Review")) OR (title:("State of the Art Review") OR abstract:("State of the Art Review")) OR (title:("State-of-the-Art Reviews") OR abstract:("State-of-the-Art Reviews")) OR (title:("Review, State-of-the-Art") OR abstract:("Review, State-of-the-Art")) OR (title:("Reviews, State-of-the-Art") OR abstract:("Reviews, State-of-the-Art")) OR (title:("State of the Art Reviews") OR abstract:("State of the Art Reviews")) OR (title:("Data Pooling") OR abstract:("Data Pooling")) OR (title:("Data Poolings") OR abstract:("Data Poolings")) OR (title:("Clinical Trial Overviews") OR abstract:("Clinical Trial Overviews")) OR (title:("Overview, Clinical Trial") OR abstract:("Overview, Clinical Trial")) OR (title:("Literature Review") OR abstract:("Literature Review")) OR (title:("Systematic Review") OR abstract:("Systematic Review")) OR (title:("Review") OR abstract:("Review")) OR (title:("Meta-analysis") OR abstract:("Meta-analysis")) |

COCHRANE DATABASE OF SYSTEMATIC REVIEWS SEARCH STRATEGY

| #1 | MeSH descriptor: [Parkinson Disease] explode all trees |
| --- | --- |
| #2 | ("Parkinson Disease"):ti,ab,kw OR ("Parkinson's Disease"):ti,ab,kw OR ("Parkinson"):ti,ab,kw |
| #3 | "Idiopathic Parkinson's Disease"):ti,ab,kw OR ("Idiopathic Parkinson Disease"):ti,ab,kw OR ("Parkinson's Disease, Idiopathic"):ti,ab,kw OR ("Parkinson Disease, Idiopathic"):ti,ab,kw |
| #4 | ("Lewy Body Parkinson's Disease"):ti,ab,kw OR ("Lewy Body Parkinson Disease"):ti,ab,kw |
| #5 | ("Primary Parkinsonism"):ti,ab,kw OR ("Parkinsonsm, Primary"):ti,ab,kw |
| #6 | ("Paralysis Agitans"):ti,ab,kw |
| #7 | #1 OR #2 OR #3 OR #4 OR #5 OR #6 |
| #8 | MeSH descriptor: [Transcranial Magnetic Stimulation] explode all trees |
| #9 | ("Transcranial Magnetic Stimulation"):ti,ab,kw OR ("Magnetic Stimulation, Transcranial"):ti,ab,kw OR ("Stimulation, Transcranial Magnetic"):ti,ab,kw OR ("Transcranial Magnetic Stimulations"):ti,ab,kw |
| #10 | ("Transcranial Magnetic Stimulation, Single Pulse"):ti,ab,kw OR ("Transcranial Magnetic Stimulation, Paired Pulse"):ti,ab,kw OR ("Transcranial Magnetic Stimulation, Repetitive"):ti,ab,kw |
| #11 | #8 OR #9 OR #10 |
| #12 | #7 AND #11 |

SCOPUS SEARCH STRATEGY

| #1 | TITLE-ABS-KEY("Parkinson Disease" OR "Parkinson's Disease" OR "Parkinson" OR "Idiopathic Parkinson's Disease" OR "Idiopathic Parkinson Disease" OR "Parkinson's Disease, Idiopathic" OR "Parkinson Disease, Idiopathic" OR "Lewy Body Parkinson's Disease" OR "Lewy Body Parkinson Disease" OR "Primary Parkinsonism" OR "Parkinsonism, Primary" OR "Paralysis Agitans") |
| --- | --- |
| #2 | TITLE-ABS-KEY("Transcranial Magnetic Stimulation" OR "Magnetic Stimulation, Transcranial" OR "Stimulation, Transcranial Magnetic" OR "Transcranial Magnetic Stimulations" OR "Transcranial Magnetic Stimulation, Single Pulse" OR "Transcranial Magnetic Stimulation, Paired Pulse" OR "Transcranial Magnetic Stimulation, Repetitive") |
| #3 | TITLE-ABS-KEY("Review Literature as Topic" OR "Systematic Review as Topic" OR "Meta-Analysis as Topic" OR "State-of-the-Art Review" OR "State of the Art Review" OR "State-of-the-Art Reviews" OR "State of the Art Reviews" OR "Review, State-of-the-Art" OR "Reviews, State-of-the-Art" OR "Data Pooling" OR "Data Poolings" OR "Clinical Trial Overviews" OR "Overview, Clinical Trial" OR "Literature Review" OR "Systematic Review" OR "Review" OR "Meta-analysis") |
| #4 | #1 AND #2 AND #3 |

WEB OF SCIENCE SEARCH STRATEGY

| #1 | AB("Parkinson Disease" OR "Parkinson's Disease" OR "Parkinson" OR "Idiopathic Parkinson's Disease" OR "Idiopathic Parkinson Disease" OR "Parkinson's Disease, Idiopathic" OR "Parkinson Disease, Idiopathic" OR "Lewy Body Parkinson's Disease" OR "Lewy Body Parkinson Disease" OR "Primary Parkinsonism" OR "Parkinsonism, Primary" OR "Paralysis Agitans") |
| --- | --- |
| #2 | AB("Transcranial Magnetic Stimulation" OR "Magnetic Stimulation, Transcranial" OR "Stimulation, Transcranial Magnetic" OR "Transcranial Magnetic Stimulations" OR "Transcranial Magnetic Stimulation, Single Pulse" OR "Transcranial Magnetic Stimulation, Paired Pulse" OR "Transcranial Magnetic Stimulation, Repetitive") |
| #3 | AB("Review Literature as Topic" OR "Systematic Review as Topic" OR "Meta-Analysis as Topic" OR "State-of-the-Art Review" OR "State of the Art Review" OR "State-of-the-Art Reviews" OR "State of the Art Reviews" OR "Review, State-of-the-Art" OR "Reviews, State-of-the-Art" OR "Data Pooling" OR "Data Poolings" OR "Clinical Trial Overviews" OR "Overview, Clinical Trial" OR "Literature Review" OR "Systematic Review" OR "Review" OR "Meta-analysis") |
| #4 | #1 AND #2 AND #3 |

# **APPENDIX A.2. Eligibility**

## Table A.2.1 References of included and excluded SRs

| Citation | Included | Excluded | Reason |
| --- | --- | --- | --- |
| Akamatsu N, Tsuji S. Present status of non-drug therapy and transcranial magnetic stimulation therapy for patients with Parkinson’s disease. Nihon Naika Gakkai zasshi The Journal of the Japanese Society of Internal Medicine. 2003;92(8):1456–60. |  | X | Language |
| Benninger DH, Hallett M. Non-invasive brain stimulation for Parkinson’s disease: Current concepts and outlook 2015. NeuroRehabilitation. 2015;37(1):11–24. |  | X | Wrong study design |
| Berardelli A. Transcranial magnetic stimulation in movement disorders. Electroencephalography and clinical neurophysiology Supplement. 1999;51:276–80. |  | X | Wrong study design |
| Cantello R. Applications of transcranial magnetic stimulation in movement disorders. Journal of Clinical Neurophysiology. 2002;19(4):272–93. |  | X | Wrong study design |
| Cantello R, Tarletti R, Civardi C. Transcranial magnetic stimulation and Parkinson’s disease. Brain Research Reviews. 2002;38(3):309–27. |  | X | Wrong study design |
| Chen J, He P, Zhang Y, Gao Y, Qiu Y, Li Y, et al. Non-pharmacological Treatment for Parkinson’s Disease Patients with Depression: A Meta-analysis of Repetitive Transcranial Magnetic Stimulation and Cognitive-Behavioral Treatment. MOVEMENT DISORDERS. 2020;35. | X |  |  |
| Cheng B, Zhu T, Zhao W, Sun L, Shen Y, Xiao W, et al. Effect of Theta Burst Stimulation-Patterned rTMS on Motor and Nonmotor Dysfunction of Parkinson’s Disease: A Systematic Review and Metaanalysis. Frontiers in neurology. 2021;12. | X |  |  |
| Chou Y hui, Hickey PT, Sundman M, Song AW, Chen N kuei. Effects of repetitive transcranial magnetic stimulation on motor symptoms in Parkinson disease: a systematic review and meta-analysis. JAMA neurology. 2015;72(4). | X |  |  |
| Chung CL, Mak MKY. Effect of Repetitive Transcranial Magnetic Stimulation on Physical Function and Motor Signs in Parkinson’s Disease: A Systematic Review and Meta-Analysis. Brain stimulation. 2016;9(4). | X |  |  |
| Deng S, Dong Z, Pan L, Liu Y, Ye Z, Qin L, et al. Effects of repetitive transcranial magnetic stimulation on gait disorders and cognitive dysfunction in Parkinson’s disease: A systematic review with meta-analysis. Brain and behavior. 2022;12(8). | X |  |  |
| Derejko M, Niewiadomska M, Rakowicz M. The diagnostic and therapeutic application of transcranial magnetic stimulation in Parkinson’s disease. Neurologia i Neurochirurgia Polska. 2005;39(5):389–96. |  | X | Language |
| Dong K, Zhu X, Xiao W, Gan C, Luo Y, Jiang M, et al. Comparative efficacy of transcranial magnetic stimulation on different targets in Parkinson’s disease: A Bayesian network meta-analysis. Frontiers in aging neuroscience. 2022;14. | X |  |  |
| Elahi B, Elahi B, Chen R. Effect of transcranial magnetic stimulation on Parkinson motor function--systematic review of controlled clinical trials. Mov Disord. 15 febbraio 2009;24(3):357–63. |  | X | No RoB |
| Fregni F, Simon DK, Wu A, Pascual-Leone A. Non-invasive brain stimulation for Parkinson’s disease: a systematic review and meta-analysis of the literature. Journal of neurology, neurosurgery, and psychiatry. 2005;76(12). |  | X | No RoB |
| Fregni F, Pascual-Leone A. Technology insight: noninvasive brain stimulation in neurology-perspectives on the therapeutic potential of rTMS and tDCS. Nature clinical practice Neurology. 2007;3(7). |  | X | No meta-analysis |
| Fujikawa J, Morigaki R, Yamamoto N, Oda T, Nakanishi H, Izumi Y, et al. Therapeutic Devices for Motor Symptoms in Parkinson’s Disease: Current Progress and a Systematic Review of Recent Randomized Controlled Trials. Frontiers in Aging Neuroscience [Internet]. 2022;14. |  | X | Wrong study design |
| Gao C, Liu J, Tan Y, Chen S. Freezing of gait in Parkinson’s disease: pathophysiology, risk factors and treatments. Transl Neurodegener. 2020;9:12. |  | X | Wrong study design |
| Godeiro C, França C, Carra RB, Saba F, Saba R, Maia D, et al. Use of non-invasive stimulation in movement disorders: a critical review. Arq Neuropsiquiatr. luglio 2021;79(7):630–46. |  | X | No meta-analysis |
| Goodwill AM, Lum JAG, Hendy AM, Muthalib M, Johnson L, Albein-Urios N, et al. Using non-invasive transcranial stimulation to improve motor and cognitive function in Parkinson’s disease: a systematic review and meta-analysis. Scientific reports. 2017;7(1). | X |  |  |
| Hai-Jiao W, Ge T, Li-Na Z, Deng C, Da X, Shan-Shan C, et al. The efficacy of repetitive transcranial magnetic stimulation for Parkinson disease patients with depression. The International journal of neuroscience. 2020;130(1). | X |  |  |
| Hanlon CA, Lench DH, Pell G, Roth Y, Zangen A, Tendler A. Bilateral deep transcranial magnetic stimulation of motor and prefrontal cortices in Parkinson’s disease: a comprehensive review. Front Hum Neurosci. 2023;17:1336027. |  | X | Wrong study design |
| Helmich RC, Siebner HR, Bakker M, Münchau A, Bloem BR. Repetitive transcranial magnetic stimulation to improve mood and motor function in Parkinson’s disease. J Neurol Sci. 25 ottobre 2006;248(1–2):84–96. |  | X | No meta-analysis |
| Hemond CC, Fregni F. Transcranial magnetic stimulation in neurology: what we have learned from randomized controlled studies. Neuromodulation. ottobre 2007;10(4):333–44. |  | X | Wrong study design |
| Hvingelby VS, Glud AN, Sørensen JCH, Tai Y, Andersen ASM, Johnsen E, et al. Interventions to improve gait in Parkinson’s disease: a systematic review of randomized controlled trials and network meta-analysis. J Neurol. agosto 2022;269(8):4068–79. |  | X | Data not available |
| Jemna N, Zdrenghea AC, Frunza G, Demea AD, Hapca GE, Grad DA, et al. Theta-burst stimulation as a therapeutic tool in neurological pathology: a systematic review. Neurol Sci. marzo 2024;45(3):911–40. |  | X | Wrong study design |
| Kim YW, Shin IS, Moon HI, Lee SC, Yoon SY. Effects of non-invasive brain stimulation on freezing of gait in parkinsonism: A systematic review with meta-analysis. Parkinsonism Relat Disord. luglio 2019;64:82–9. |  | X | Wrong intervention |
| Krogh S, Jønsson AB, Aagaard P, Kasch H. Efficacy of repetitive transcranial magnetic stimulation for improving lower limb function in individuals with neurological disorders: A systematic review and meta-analysis of randomized sham-controlled trials. Journal of rehabilitation medicine. 2022;54. | X |  |  |
| Li R, He Y, Qin W, Zhang Z, Su J, Guan Q, et al. Effects of Repetitive Transcranial Magnetic Stimulation on Motor Symptoms in Parkinson’s Disease: A Meta-Analysis. Neurorehabilitation and neural repair. 2022;36(7). | X |  |  |
| Li S, Jiao R, Zhou X, Chen S. Motor recovery and antidepressant effects of repetitive transcranial magnetic stimulation on Parkinson disease: A PRISMA-compliant meta-analysis. Medicine (Baltimore). maggio 2020;99(18):e19642. |  | X | No RoB |
| Lin X, Zhang Y, Chen X, Wen L, Duan L, Yang L. Effects of noninvasive brain stimulation on dual-task performance in different populations: A systematic review. Front Neurosci. 2023;17:1157920. |  | X | Wrong study design |
| Liu X, Li L, Liu Y. Comparative motor effectiveness of non-invasive brain stimulation techniques in patients with Parkinson’s disease: A network meta-analysis. Medicine. 2023;102(39). | X |  |  |
| Liu Z, Wen X, Xie X, Liu Y, Tan C, Kuang S, et al. The effects of transcranial magnetic stimulation for freezing of gait in Parkinson’s disease: a systematic review and meta-analysis of randomized controlled trials. Frontiers in aging neuroscience. 2024;16. | X |  |  |
| Madrid J, Benninger DH. Non-invasive brain stimulation for Parkinson’s disease: Clinical evidence, latest concepts and future goals: A systematic review. Journal of Neuroscience Methods [Internet]. 2021;347. |  | X | Wrong study design |
| Matsumoto H, Ugawa Y. Repetitive transcranial magnetic stimulation for Parkinson’s disease: A review. Brain and Nerve. 2017;69(3):219–25. |  | X | Language |
| Nardone R, Versace V, Brigo F, Golaszewski S, Carnicelli L, Saltuari L, et al. Transcranial magnetic stimulation and gait disturbances in Parkinson’s disease: A systematic review. Neurophysiologie Clinique. 2020;50(3):213–25. |  | X | Wrong study design |
| Nehra A, Sharma PS, Narain A, Kumar A, Bajpai S, Rajan R, et al. The Role of Repetitive Transcranial Magnetic Stimulation for Enhancing the Quality of Life in Parkinson’s Disease: A Systematic Review. Ann Indian Acad Neurol. dicembre 2020;23(6):755–9. |  | X | No meta-analysis |
| Pateraki G, Anargyros K, Aloizou AM, Siokas V, Bakirtzis C, Liampas I, et al. Therapeutic application of rTMS in neurodegenerative and movement disorders: A review. J Electromyogr Kinesiol. febbraio 2022;62:102622. |  | X | Wrong study design |
| Qin B, Chen H, Gao W, Zhao LB, Zhao MJ, Qin HX, et al. Effectiveness of high-frequency repetitive transcranial magnetic stimulation in patients with depression and Parkinson’s disease: a meta-analysis of randomized, controlled clinical trials. Neuropsychiatric disease and treatment. 2018;14. | X |  |  |
| Qiu Y, Yin Z, Wang M, Duan A, Xie M, Wu J, et al. Motor function improvement and acceptability of non-invasive brain stimulation in patients with Parkinson’s disease: a Bayesian network analysis. Frontiers in neuroscience. 2023;17. | X |  |  |
| Siebner H. Treatment of movement disorders: Importance of repetitive transcranial magnetic stimulation. Neurophysiologie-Labor. 2011;33(3–4):118–22. |  | X | Language |
| Tayupova GN, Saitgareeva AR, Baitimerov AR, Levin OS. [Transcranial magnetic stimulation in Parkinson’s disease]. Zh Nevrol Psikhiatr Im S S Korsakova. 2016;116(6):82–7. |  | X | Language |
| Vadalà M, Vallelunga A, Palmieri L, Palmieri B, Morales-Medina JC, Iannitti T. Mechanisms and therapeutic applications of electromagnetic therapy in Parkinson’s disease. Behav Brain Funct. 7 settembre 2015;11:26. |  | X | Wrong study design |
| Wagle Shukla A, Shuster JJ, Chung JW, Vaillancourt DE, Patten C, Ostrem J, et al. Repetitive Transcranial Magnetic Stimulation (rTMS) Therapy in Parkinson Disease: A Meta-Analysis. PM & R : the journal of injury, function, and rehabilitation. 2016;8(4). | X |  |  |
| Wang P, Gou Y, Liao WJ, Li HG. Effectiveness of high- and low-frequency repetitive transcranial magnetic stimulation for treating dysfunction in patients with Parkinson’s disease: A meta-analysis. Chinese Journal of Evidence-Based Medicine. 2010;10(11):1308–15. |  | X | Language |
| Xia Y, Wang M, Zhu Y. The Effect of Cerebellar rTMS on Modulating Motor Dysfunction in Neurological Disorders: a Systematic Review. Cerebellum. ottobre 2023;22(5):954–72. |  | X | No meta-analysis |
| Xie CL, Chen J, Wang XD, Pan JL, Zhou Y, Lin SY, et al. Repetitive transcranial magnetic stimulation (rTMS) for the treatment of depression in Parkinson disease: a meta-analysis of randomized controlled clinical trials. Neurological sciences : official journal of the Italian Neurological Society and of the Italian Society of Clinical Neurophysiology. 2015;36(10). | X |  |  |
| Xie YJ, Gao Q, He CQ, Bian R. Effect of Repetitive Transcranial Magnetic Stimulation on Gait and Freezing of Gait in Parkinson Disease: A Systematic Review and Meta-analysis. Archives of physical medicine and rehabilitation. 2020;101(1). | X |  |  |
| Yang C, Guo Z, Peng H, Xing G, Chen H, McClure MA, et al. Repetitive transcranial magnetic stimulation therapy for motor recovery in Parkinson’s disease: A Meta-analysis. Brain and behaviour. 2018;8(11). | X |  |  |
| Zanjani A, Zakzanis KK, Daskalakis ZJ, Chen R. Repetitive transcranial magnetic stimulation of the primary motor cortex in the treatment of motor signs in Parkinson’s disease: A quantitative review of the literature. Mov Disord. maggio 2015;30(6):750–8. |  | X | No RoB |
| Zhang W, Deng B, Xie F, Zhou H, Guo JF, Jiang H, et al. Efficacy of repetitive transcranial magnetic stimulation in Parkinson’s disease: A systematic review and meta-analysis of randomised controlled trials. EClinicalMedicine. 2022;52. | X |  |  |
| Zhang X, Jing F, Liu Y, Tang J, Hua X, Zhu J, et al. Effects of non-invasive brain stimulation on walking and balance ability in Parkinson’s patients: A systematic review and meta-analysis. Frontiers in aging neuroscience. 2022;14. | X |  |  |
| Zhao XF, Lei J, Zhang XN, Xie C, Dong CL, Wang XB. Clinical effects of repetitive transcranial magnetic stimulation therapy on Parkinson’s disease: a Meta-analysis. Chinese Journal of Contemporary Neurology and Neurosurgery. 2015;15(4):302–10. |  | X | Language |
| Zhu H, Lu Z, Jin Y, Duan X, Teng J, Duan D. Low-frequency repetitive transcranial magnetic stimulation on Parkinson motor function: a meta-analysis of randomised controlled trials. Acta neuropsychiatrica. 2015;27(2). | X |  |  |

# **APPENDIX A.3. Characteristics of the included SRs**

## Table A.3.1 General characteristics for each SRs

| Author | Year | Search strategy | N. of included trials | Population | Mean age (yr)  [mean±SD] | Disease severity  [H&Y] | Disease duration (yr) | Intervention  (type of rTMS) | Conflict of interest | Sources of funding | OUTCOME |
| --- | --- | --- | --- | --- | --- | --- | --- | --- | --- | --- | --- |
| Chou | 2015 | From inception until June 30, 2014 | 20 | 470 | 63.59 ± 8.24 | NA* | NA* | HF-rTMS > 1Hz  (range 1-50 Hz)  LF-rTMS < 1Hz  (range 1-0,2 Hz) | No | Yes | UPDRS – III |
| Xie, C.L. | 2015 | From January 1990 to May 2014 | 8 | 312 | 63.79 ± 7.34 | NA | NA | HF-rTMS > 1Hz  (range 1-15 Hz)  LF-rTMS < 1Hz  (range 1-0,5 Hz) | No | No | UPDRS – III |
| Zhu | 2015 | From inception until June 2014 | 8 | 319 | NA* | NA* | NA* | LF-rTMS < 1Hz  (range 1-0,2 Hz) | No | No | UPDRS – III |
| Chung | 2016 | From January 1966 to July 16, 2015 | 22 | 555 | NA | NA* | NA | HF-rTMS > 1Hz  (range 1-50 Hz)  LF-rTMS < 1 Hz  (range 1-0,2 Hz) | No | Yes | UPDRS – III |
| Wagle Shukla | 2016 | From 1980 to 2013 | 21 | 560 | NA* | NA* | NA* | HF-rTMS ≥ 5 Hz  (range 5-50 Hz)  ITBS 50Hz  LF-rTMS ≤1 Hz  (range 1-0,5 Hz) | No | Yes | UPDRS – III |
| Goodwill | 2017 | From inception until February 26, 2016 | 24 | 651 | 64.00 ± 3.80 | 1 - 5 | 8 ± 3.7 | HF-rTMS > 1Hz (range 1-50 Hz)  LF-rTMS ≤1 Hz (range 1-0,2 Hz) | No | Yes | UPDRS – III |
| Qin | 2018 | From inception until July 2017 | 9 | 332 | 63.85 ± 11.76 | NA | NA | HF-rTMS > 5 Hz  (range 5-15 Hz) | No | No | UPDRS – III |
| Yang | 2018 | From inception until April 11, 2018 | 23 | 646 | NA* | NA* | NA* | HF-rTMS > 1 Hz (range 1-50 Hz)  LF-rTMS ≤1 Hz (range 1-0,5 Hz) | No | No | UPDRS – III |
| Chen | 2020 | From inception until December 31, 2018 | 12 | 511 | NA | NA | NA | HF-rTMS > 1 Hz  (range 5-15 Hz)  LF-rTMS ≤1 Hz (range 1-0,5 Hz) | No | Yes | UPDRS – III |
| Hai-Jiao | 2020 | From inception until October 19, 2017 | 7 | 199 | NA* | NA | NA* | HF-rTMS > 1Hz  (range 5-15 Hz) | No | No | UPDRS – III |
| Xie,Y. J. | 2020 | From inception until March 28, 2019 | 14 | 298 | 63.24 ± 9.71 | NA* | NA* | HF-rTMS > 1 Hz (range 1-50 Hz) | No | Not reported | Walking time; TUG; FOG |
| Cheng | 2021 | From January 1, 2005, to September 30, 2021 | 8 | 189 | NA* | NA* | NA* | ITBS  (Range 30-50Hz) | No | Yes | UPDRS – III; Walking time |
| Deng | 2022 | From inception until December 31, 2021 | 16 | 419 | From 59.94 ± 9.16 to 74.57 ± 7.09 | 2 - 3 | From 2.5 ± 1.1 to 12.0 ± 6.3 | HF-rTMS > 1Hz (range 1-50 Hz)  ITBS 50 Hz | No | Yes | Walking time; TUG; FOG |
| Dong | 2022 | From inception until December, 2021 | 36 | 1122 | NA | NA | NA | HF-rTMS > 1Hz  (range 1-50 Hz)  LF-rTMS < 1Hz  (range 1-0,5 Hz) | No | Yes | UPDRS – III |
| Krogh | 2022 | From inception until March 31, 2021 | 11 | 359 | NA* | NA* | NA* | HF-rTMS ≥ 5 Hz  (range 5-50 Hz)  ITBS 50 Hz  LF-rTMS = 1Hz | No | Yes | TUG; FOG |
| Li | 2022 | From inception until December 31, 2021 | 30 | 1035 | NA* | NA* | NA* | HF-rTMS ≥ 5  (range 5-50 Hz)  ITBS 50 Hz  LF-rTMS ≤1  (range 1-0,2Hz) | No | Yes | UPDRS – III  TUG; FOG; Walking time; |
| Zhang, W. | 2022 | From January 1, 1988 to January 1, 2022 | 14 | 469 | 64.38 ± 8.76 | NA* | NA* | HF-rTMS ≥ 5  (range 5-50 Hz)  LF-rTMS = 1Hz | No | Yes | UPDRS – III |
| Zhang, X. | 2022 | From inception until June 2022 | 18 | 906 | NA* | 1 - 5 | From 1.64 to 10.8 | HF-rTMS ≥ 5  (range 5-50 Hz)  LF-rTMS = 1Hz | No | Yes | UPDRS – III; TUG |
| Liu, X. | 2023 | From inception until August 2022 | 25 | 999 | NA* | NA* | NA* | HF-rTMS ≥ 5  (range 5-50 Hz)  LF-rTMS ≤ 1 Hz | No | No | UPDRS – III |
| Qiu | 2023 | From January 1, 2013, to January 1, 2023 | 16 | 531 | NA* | NA* | NA | HF-rTMS ≥ 5  (range 5-50 Hz)  LF-rTMS = 1Hz | No | Yes | UPDRS – III  TUG; FOG |
| Liu, Z. | 2024 | From inception until July 2023 | 16 | 408 | NA* | NA* | From 3.5 to 13.8 | HF-rTMS ≥ 5 Hz  (range 5-50 Hz)  ITBS 50 Hz  LF-rTMS = 1Hz | No | No | Walking time, TUG |

yr = years; SD = Standard Deviation; H&Y = Hoehn & Yahr; NA = Not Available

* The mean value cannot be calculated due to missing data from some primary studies

# **APPENDIX A.4. Overlapping of primary studies included in SRs**

## Figure A.4.1 Overlapping SRs of primary studies for all studies


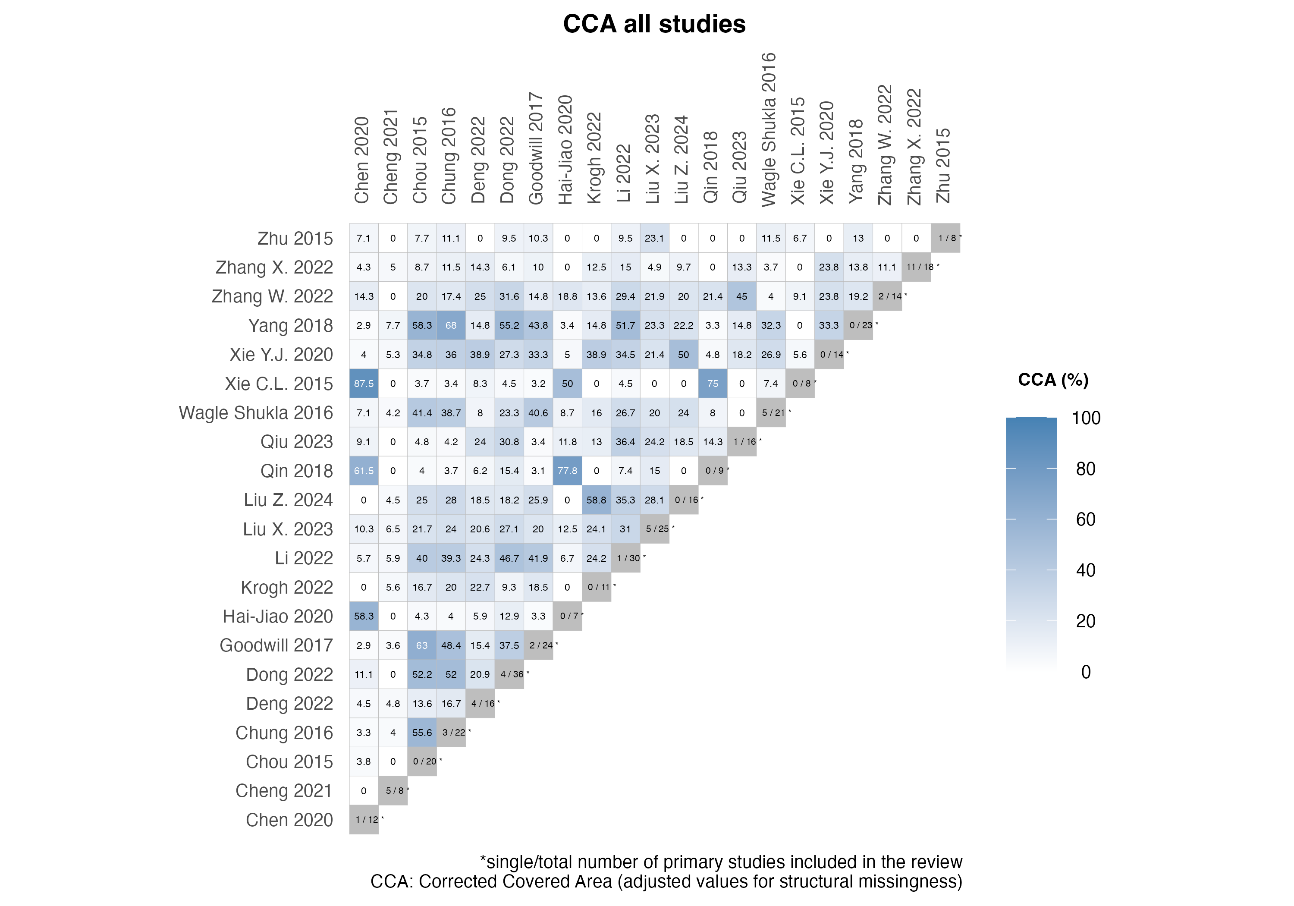


Heatmap for the overall Corrected Covered Area, values adjusted for structural missingness.

## Table A.4.1 Overlapping SRs of primary studies for all studies

| Reviews | ***N*** | **r** | **c** | **Structural**  **Missingness** | **CCA_Proportion** | **CCA_Percentage** |
| --- | --- | --- | --- | --- | --- | --- |
| 21 | 358 | 107 | 21 | 459 | 0,149316 | 14,9 |

Abbreviations: N = number of primary studies including overlaps; r = number of index publications; c = number of reviews; CCA_Proportion = proportion of corrected covered area for pairs of reviews and overall area; CCA_Percentage = proportion of corrected covered area for pairs of reviews and overall rounded to the first decimal place.

## Figure A.4.2 Overlapping SRs of primary studies for Unified Parkinson’s Disease Rating Scale – Part III outcome


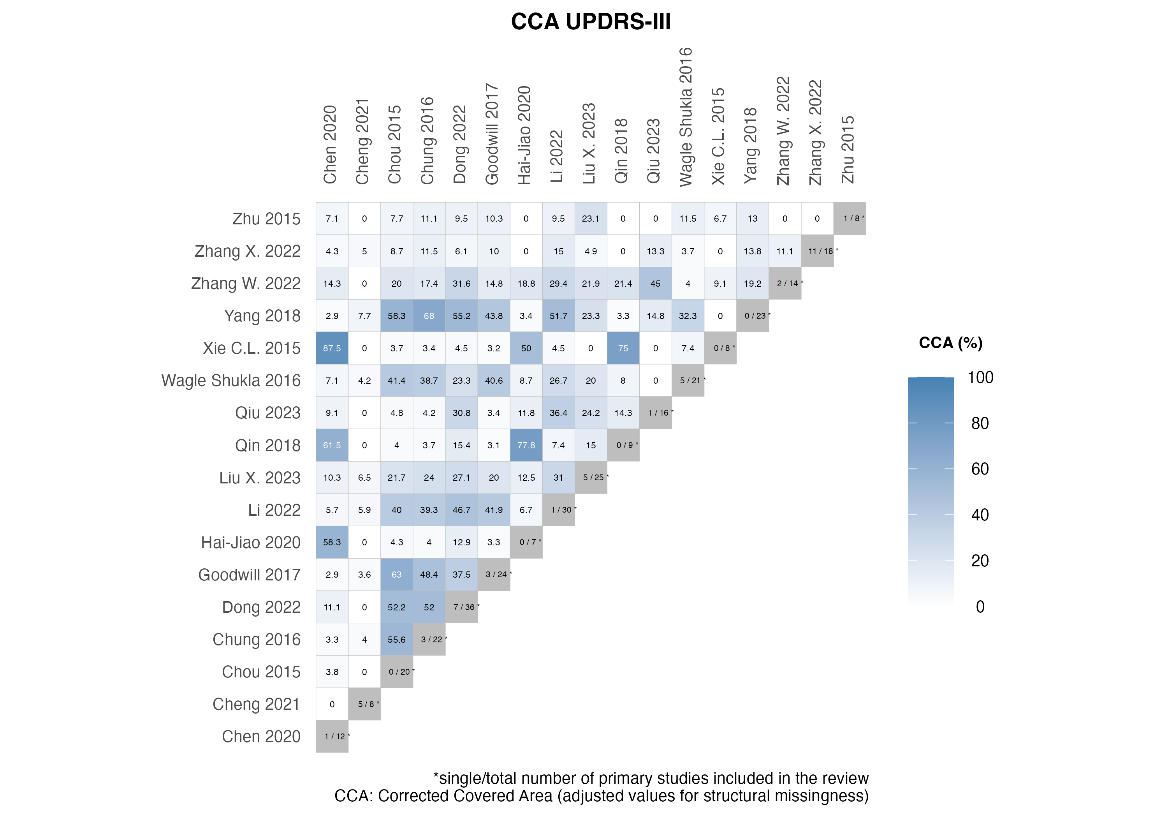


Heatmap for the Corrected Covered Area for the UPDRS-III outcome, values adjusted for structural missingness.

## Table A.4.2 Overlapping SRs of primary studies for Unified Parkinson’s Disease Rating Scale – Part III outcome

| Reviews | ***N*** | **r** | **c** | **Structural**  **Missingness** | **CCA_Proportion** | **CCA_Percentage** |
| --- | --- | --- | --- | --- | --- | --- |
| 17 | 301 | 102 | 17 | 399 | 0,161395 | 16,1 |

Abbreviations: N = number of primary studies including overlaps; r = number of index publications; c = number of reviews; CCA_Proportion = proportion of corrected covered area for pairs of reviews and overall area; CCA_Percentage = proportion of corrected covered area for pairs of reviews and overall rounded to the first decimal place.

## Figure A.4.3 Overlapping SRs of primary studies for Freezing of Gait outcome


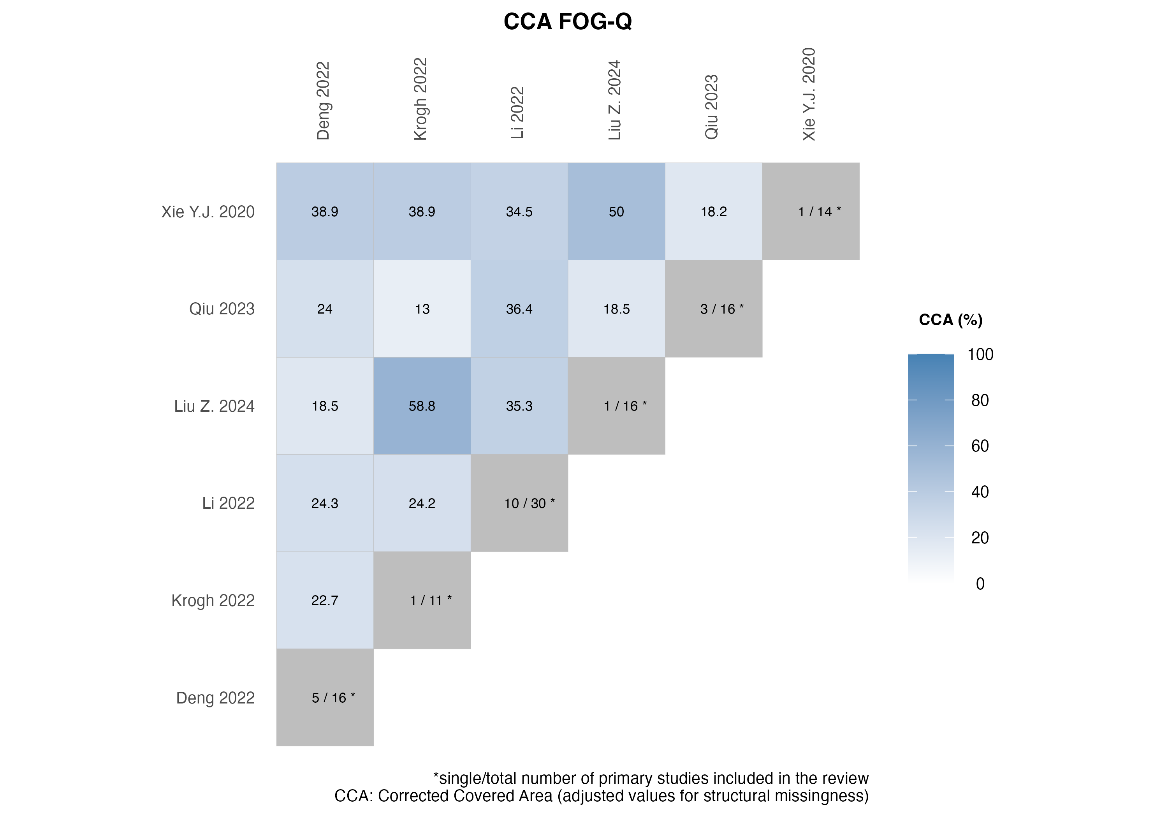


Heatmap for the Corrected Covered Area for the Freezing og Gait Questionnaire outcome, values adjusted for structural missingness.

## Table A.4.3 Overlapping SRs of primary studies for Freezing of Gait outcome

| Reviews | ***N*** | **r** | **c** | **Structural**  **Missingness** | **CCA_Proportion** | **CCA_Percentage** |
| --- | --- | --- | --- | --- | --- | --- |
| 6 | 103 | 46 | 6 | 14 | 0,263889 | 26,4 |

Abbreviations: N = number of primary studies including overlaps; r = number of index publications; c = number of reviews; CCA_Proportion = proportion of corrected covered area for pairs of reviews and overall area; CCA_Percentage = proportion of corrected covered area for pairs of reviews and overall rounded to the first decimal place.

## Figure A.4.4 Overlapping SRs of primary studies for Time Up and Go outcome


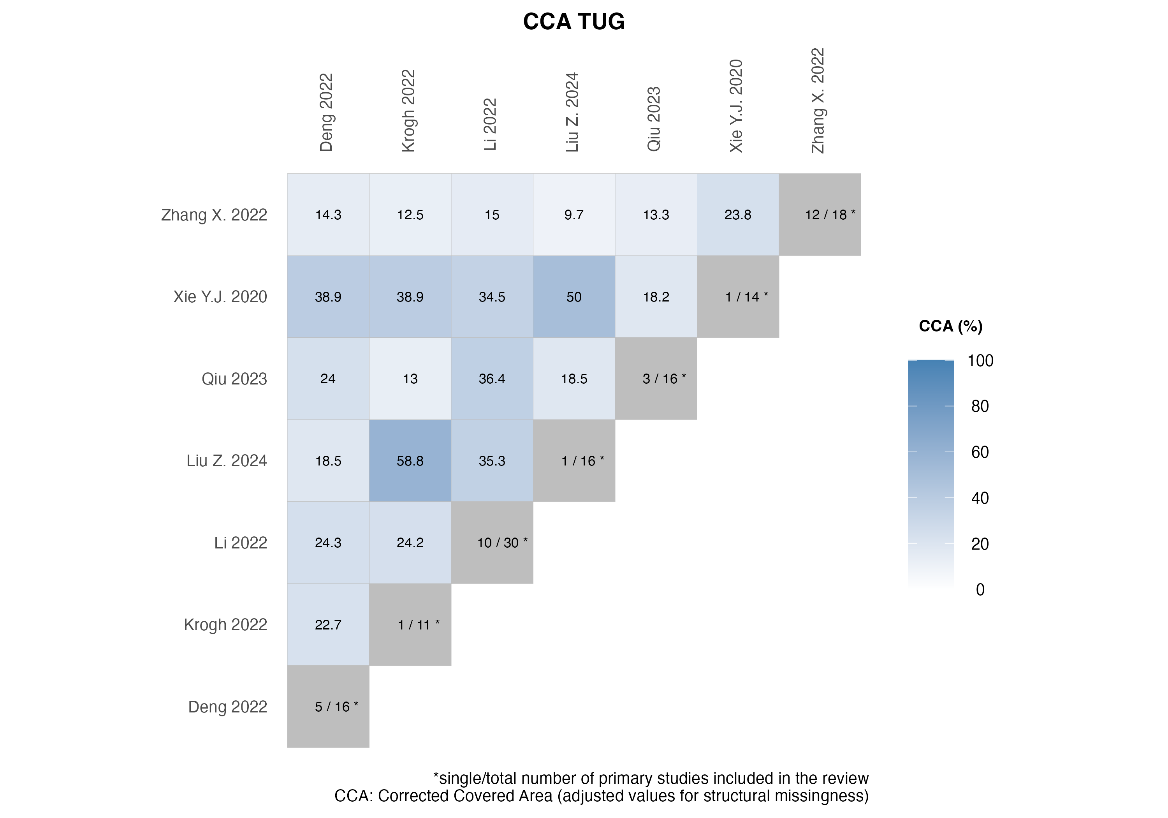


Heatmap for the Corrected Covered Area for the Time Up and Go outcome, values adjusted for structural missingness.

## Table A.4.4 Overlapping SRs of primary studies for Time Up and Go outcome

| Reviews | ***N*** | **r** | **c** | **Structural**  **Missingness** | **CCA_Proportion** | **CCA_Percentage** |
| --- | --- | --- | --- | --- | --- | --- |
| 7 | 121 | 58 | 7 | 26 | 0,195652 | 19,6 |

Abbreviations: N = number of primary studies including overlaps; r = number of index publications; c = number of reviews; CCA_Proportion = proportion of corrected covered area for pairs of reviews and overall area; CCA_Percentage = proportion of corrected covered area for pairs of reviews and overall rounded to the first decimal place.

## Figure A.4.5 Overlapping SRs of primary studies for Walking Time outcome


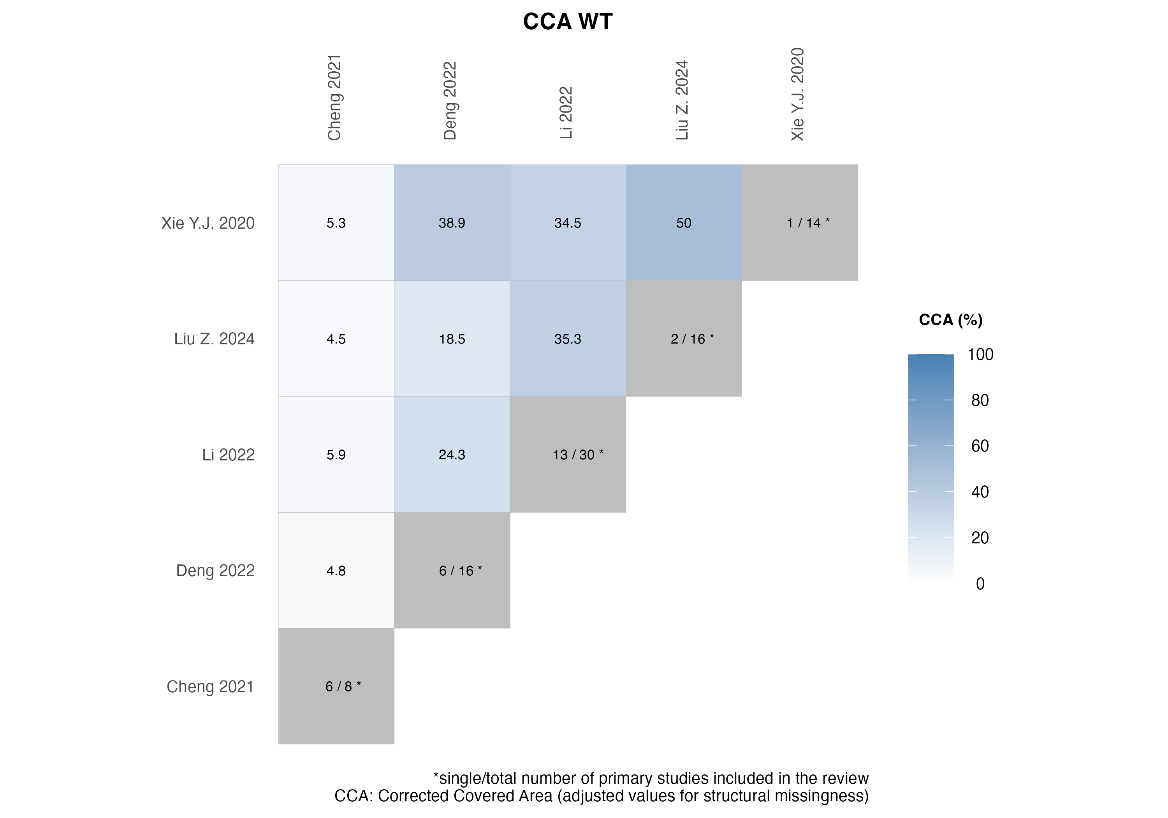


Heatmap for the Corrected Covered Area for the Walking Time outcome, values adjusted for structural missingness.

## Table A.4.5 Overlapping SRs of primary studies for Walking Time outcome

| Reviews | ***N*** | **r** | **c** | **Structural**  **Missingness** | **CCA_Proportion** | **CCA_Percentage** |
| --- | --- | --- | --- | --- | --- | --- |
| 5 | 84 | 48 | 5 | 17 | 0,205714 | 20,6 |

Abbreviations: N = number of primary studies including overlaps; r = number of index publications; c = number of reviews; CCA_Proportion = proportion of corrected covered area for pairs of reviews and overall area; CCA_Percentage = proportion of corrected covered area for pairs of reviews and overall rounded to the first decimal place.

# **APPENDIX A.5. Review quality assessment AMSTAR 2**

## Table A.5.1 AMSTAR 2 assessment for each review

| Author | Year | 1 | 2 | 3 | 4 | 5 | 6 | 7 | 8 | 9 | 10 | 11 | 12 | 13 | 14 | 15 | 16 | Overall |
| --- | --- | --- | --- | --- | --- | --- | --- | --- | --- | --- | --- | --- | --- | --- | --- | --- | --- | --- |
| Chou | 2015 | Yes | Yes | Yes | Yes | No | Yes | No | Yes | Yes | No | Yes | No | Yes | Yes | Yes | Yes | Low |
| Xie, C.L. | 2015 | Yes | No | No | Yes | No | Yes | No | Yes | Yes | No | Yes | No | Yes | Yes | Yes | Yes | Critically Low |
| Zhu | 2015 | No | No | Yes | Yes | Yes | Yes | No | Yes | Yes | No | Yes | Yes | Yes | Yes | Yes | Yes | Critically Low |
| Chung | 2016 | Yes | No | No | Yes | Yes | Yes | No | Yes | Yes | No | Yes | Yes | Yes | Yes | Yes | Yes | Critically Low |
| Wagle Shukla | 2016 | No | No | Yes | Yes | No | Yes | No | Yes | Yes | No | Yes | No | No | No | No | Yes | Critically Low |
| Goodwill | 2017 | No | Yes | No | Yes | Yes | Yes | No | Yes | Yes | No | Yes | No | No | Yes | Yes | Yes | Critically Low |
| Qin | 2018 | Yes | Yes | No | Yes | Yes | Yes | No | Yes | Yes | No | Yes | No | Yes | Yes | No | Yes | Critically Low |
| Yang | 2018 | No | No | No | Yes | No | Yes | No | Yes | P.Y. | No | Yes | No | No | Yes | Yes | Yes | Critically Low |
| Chen | 2020 | Yes | No | No | P.Y. | No | No | No | Yes | Yes | No | Yes | No | Yes | Yes | Yes | Yes | Critically Low |
| Hai-Jiao | 2020 | Yes | No | No | P.Y. | Yes | Yes | No | Yes | Yes | No | Yes | No | Yes | Yes | No | Yes | Critically Low |
| Xie,Y. J. | 2020 | Yes | No | No | Yes | Yes | Yes | No | Yes | Yes | No | Yes | Yes | Yes | Yes | Yes | No | Critically Low |
| Cheng | 2021 | Yes | No | No | Yes | Yes | Yes | No | Yes | Yes | No | Yes | No | No | Yes | Yes | Yes | Critically Low |
| Deng | 2022 | Yes | Yes | Yes | Yes | Yes | Yes | No | Yes | Yes | No | Yes | No | Yes | Yes | Yes | Yes | Low |
| Dong | 2022 | Yes | Yes |  | P.Y. | Yes | No | No | Yes | Yes | No | Yes | No | Yes | Yes | Yes | Yes | Low |
| Krogh | 2022 | Yes | Yes | Yes | P.Y. | Yes | Yes | No | Yes | Yes | No | Yes | No | Yes | Yes | No | Yes | Critically Low |
| Li | 2022 | Yes | No | No | P.Y. | Yes | Yes | No | P.Y. | Yes | No | Yes | No | No | Yes | Yes | Yes | Critically Low |
| Zhang, W. | 2022 | No | Yes | No | Yes | No | Yes | No | P.Y. | Yes | No | Yes | No | No | Yes | Yes | Yes | Critically Low |
| Zhang, X. | 2022 | No | Yes | No | Yes | Yes | No | No | Yes | Yes | No | Yes | No | Yes | Yes | Yes | Yes | Low |
| Liu, X. | 2023 | Yes | Yes | No | Yes | Yes | Yes | No | Yes | Yes | No | Yes | No | No | Yes | Yes | Yes | Critically Low |
| Qiu | 2023 | Yes | Yes | No | Yes | Yes | Yes | No | P.Y. | Yes | No | Yes | No | No | Yes | Yes | Yes | Critically Low |
| Liu, Z. | 2024 | Yes | Yes | No | Yes | Yes | Yes | No | Yes | Yes | No | Yes | No | Yes | Yes | No | Yes | Critically Low |

1-Question and inclusion; 2-Protocol; 3-Study design; 4-Comprehensive search; 5-Study selection; 6-Data extraction; 7-Excluded studied justification; 8-Included studied details; 9-Risk of Bias; 10-Source of funding of included studies; 11-Appropriate statistical methods for analysis; 12-Rob on meta-analyses; 13-Rob on individual studies; 14-Explanation for heterogeneity; 15-Publication bias; 16-Conflict of interest. Items 2-4-7-9-11-13-15 were critical. Legend: P.Y. = partially yes.

# **APPENDIX A.6. Risk of bias assessment in primary studies included in SRs**

## Table A.6.1 Risk of bias in primary studies

| Author | Year | Number of RCT | Assessment scale | RESULTS | | | | | |
| --- | --- | --- | --- | --- | --- | --- | --- | --- | --- |
|  |  |  | *Modified CONSORT checklist* | **6 Points** | **5 Points** | | **4 Points** | | **≤ 3 Points** |
| Yang | 2018 | 23 |  | 18 | 2 | | 3 | | - |
|  |  |  | *Jadad scale* | **High [4/5]** | | | **Poor [0-3]** | | |
| Hai-Jiao* | 2020 | 7 |  | 7 | | | - | | |
|  | | | *PEDro scale* | **Excellent [9-10]** | **Good [6-8]** | | **Fair [4-5]** | | **Poor [<4]** |
| Chou° | 2015 | 20 |  | 13 | 7 | | - | | - |
| Wagle Shukla | 2016 | 21 |  | 3 | 6 | | 7 | | 5 |
| Goodwill | 2017 | 24 |  | 6 | 15 | | 3 | | - |
| Xie,Y. J. | 2020 | 14 |  | 2 | 11 | | 1 | | - |
| Cheng | 2021 | 8 |  | 2 | 5 | | - | | - |
| Li | 2022 | 30 |  | 10 | 20 | | - | | - |
|  | | | *Cochrane*  *Risk of Bias Tool* | **Low** | | **Unclear** | | **High** | |
| Chou° | 2015 | 20 |  | 11 | | 3 | | 6 | |
| Xie, C.L. | 2015 | 8 |  | 2 | | - | | 6 | |
| Zhu | 2015 | 8 |  | 4 | | 4 | | - | |
| Chung | 2016 | 22 |  | 6 | | 11 | | 5 | |
| Goodwill | 2017 | 24 |  | 9 | | 15 | | - | |
| Qin | 2018 | 9 |  | - | | 7 | | 2 | |
| Chen | 2020 | 12 |  | - | | 10 | | 2 | |
| Hai-Jiao* | 2020 | 7 |  | 1 | | 2 | | 4 | |
| Deng | 2022 | 16 |  | - | | - | | 16 | |
| Dong | 2022 | 36 |  | 10 | | 21 | | 5 | |
| Krogh | 2022 | 11 |  | 4 | | 5 | | 2 | |
| Zhang, W. | 2022 | 14 |  | 14 | | - | | - | |
| Zhang, X. | 2022 | 18 |  | - | | 9 | | 9 | |
| Liu, X. | 2023 | 25 |  | 11 | | 12 | | 2 | |
| Qiu | 2023 | 16 |  | 8 | | 2 | | 6 | |
| Liu, Z. | 2024 | 16 |  | 13 | | - | | 3 | |

* Hai-Jiao (2020) used more than one Risk of Bias assessment tool: Jadad scale e Risk of Bias Tool;° Chou (2015) used more than one Risk of Bias assessment tool: PEDro scale e Risk of Bias Tool

# **APPENDIX A.7. Strategy for data synthesis and statistical analysis**

## Table A.7.1 Mean Difference and Standardized Mean Difference conversion of main outcomes

| **Author, Year** | **SD** | **MD** | **SMD** | **Author, Year** | **SD** | **MD** | **SMD** |
| --- | --- | --- | --- | --- | --- | --- | --- |
| **UPDRS-III** | | | | **FOG-Q** | | | |
| Chou 2015 | 6.2 | **-2.85** | *-0.46* | Xie YJ 2020 | 5.1 | **-4.13** | *-0.81* |
| Xie CL 2015 | 5.37 | **-1.83** | *-0.34* | Deng (a) 2022 | 5.1 | *-0.92* | **-0.18** |
| Wagle Shukla 2016 | 6.2 | *-4.1* | **-0.66** | Deng (b) 2022 | 5.1 | *-2.12* | **-0.42** |
| Goodwill 2017 | ***6.2*** | ***-2.3*** | *-0.37* | Krogh 2022 | 0.86 | **-1.14** | *-1.33* |
| Hai-Jiao 2020 | 14.8 | *-3.01* | **-0.2** | Li 2022 | 5.1 | **-1.99** | *-0.39* |
| Dong 2022 | 6.2 | *-3.72* | **-0.6** | Qiu (a) 2023 | 5.1 | *-1.27* | **-0.25** |
| Li 2022 | 6.2 | **-3.96** | *-0.64* | Qiu (b) | 5.1 | *-1.68* | **-0.33** |
| Zhang W 2022 | 21.24 | **-11.68** | *-0.55* | Liu Z 2024 | 0.86 | **-0.47** | *-0.55* |
| Zhang X 2022 | 4.19 | *-2.54* | **-0.61** |  |  |  |  |
| **TUG** | | | | **WT** | | | |
| Xie YJ 2020 | 0.7 | **-0.31** | *-0.45* | Xie YJ 2020 | 10.4 | **-3.12** | *-0.3* |
| Deng (a) 2022 | 0.7 | *-1.064* | **-1.52** | Cheng 2021 | 6.6 | **-2.44** | *-0.37* |
| Deng (b) 2022 | 0.7 | *-1.097* | **-1.56** | Deng (a) 2022 | 2.02 | *-0.456* | **-0.22** |
| Krogh 2022 | 0.92 | **-2.06** | *-2.24* | Deng (b) 2022 | 2.02 | *-0.526* | **-0.26** |
| Li 2022 | 0.7 | **-0.63** | *-0.9* | Li 2022 | 10.4 | **-9.88** | *-0.95* |
| Zhang X 2022 | 1.93 | *-4.11* | **-2.13** | Liu Z 2024 | 2.02 | **-0.83** | *-0.41* |
| Qiu (a) 2023 | 0.92 | *-2.04* | **-2.21** |  |  |  |  |
| Qiu (b) 2023 | 0.92 | *-2.66* | **-2.89** |  |  |  |  |
| Liu Z 2024 | 0.82 | **-0.46** | *-0.56* |  |  |  |  |

SD= Values of Standard Deviations most representative control group of each meta-analysis; MD= Mean Difference; SMD= Standardized Mean Difference. The values in italics are the original effect sizes extracted from the reviews; the values in bold are the converted effect sizes. (a)=short term; (b)= long term.

## Table A.7.2 Mean Difference and Standardized Mean Difference conversion of UPDRS – III Subgroups

| Author, Year | **SD** | **MD** | **SMD** | | **Author, Year** | **SD** | **MD** | | **SMD** |
| --- | --- | --- | --- | --- | --- | --- | --- | --- | --- |
| Timing of outcome measurements | | | | | | | | | |
| Short term | | | | | Long term | | | | |
| Chou 2015 | 6.2 | **-3.1** | | *-0.5* | Chou 2015 | 6.2 | | **-3.658** | *-0.59* |
| Chung 2016 | 6.2 | **-1.92** | | *-0.31* | Chung 2016 | 6.2 | | **-3.348** | *-0.54* |
| Wagle Shukla 2016 | 6.2 | *-3.5* | | **-0.56** | Wagle Shukla 2016 | 6.2 | | *-4.1* | **-0.66** |
| Yang 2018 | 6.2 | **-2.29** | | *-0.37* | Yang 2018 | 6.2 | | **-2.356** | *-0.38* |
| Chen 2020 | 5.37 | *-2.617* | | **-0.48** | Chen 2020 | 5.37 | | *-1.625* | **-0,3** |
| Frequency | | | | | | | | | |
| Low frequency | | | | | High frequency | | | | |
| Zhu 2015 | 6.2 | **-1.86** | | *-0.32* | Chung 2016 | 6.2 | | **-0.41** | *-0.067* |
| Chung 2016 | 6.2 | **-2.04** | | *-0.168* | Wagle Shukla 2016 | 6.2 | | *-3.9* | **-0.62** |
| Wagle Shukla 2016 | 6.2 | *-3.3* | | **-0.53** | Qin 2018 | 11.05 | | *-2.8* | **-2.25** |
| Yang 2018 | 6.2 | **-1.178** | | *-0.19* | Yang 2018 | 6.2 | | **-2.97** | *-0.48* |
| Dong 2022 | 6.2 | *-1.21* | | **-0.195** | Dong 2022 | 6.2 | | *-4.63* | **-0.74** |
| Li 2022 | 6.2 | **-1.55** | | *-0.25* | Li 2022 | 6.2 | | **-4.52** | *-0.73* |
| Zhang W 2022 | 21.24 | **-2.12** | | *-0.1* | Zhang W 2022 | 21.24 | | **-11.89** | *-0.56* |
| Qiu 2023a | 7.3 | *-3.23* | | **-2.26** | Qiu 2023a | 7.3 | | *-5.21* | **-1.4** |
| Qiu 2023b | 7.3 | *-4.83* | | **-1.51** | Qiu 2023b | 7.3 | | *-4.74* | **-1.54** |
| Application site | | | | | | | | | |
| M1 | | | | | SMA | | | | |
| Chung 2016 | 6.2 | **-2.88** | | *-0.465* | Chung 2016 | 6.2 | | **-1.829** | *-0.295* |
| Yang 2018 | 6.2 | **-3.22** | | *-0.52* | Yang 2018 | 6.2 | | **-1.86** | *-0.3* |
| Li 2022 | 6.2 | **-3.96** | | *-0.64* | Li 2022 | 6.2 | | **-2.6** | *-0.42* |
| DLPFC | | | | | M1+DLPRC | | | | |
| Chung 2016 | 6.2 | **0.5** | | *0.081* | Yang 2018 | 6.2 | | **-0.62** | *-0.1* |
| Yang 2018 | 6.2 | **-1.798** | | *-0.29* |  |  | |  |  |
| Li 2022 | 6.2 | **-5.02** | | *-0.81* |  |  | |  |  |
| Frequency - Application site | | | | | | | | | |
| LF-M1 | | | | | HF-M1 | | | | |
| Chou 2015 | 6.2 | **-1.73** | | *-0.28* | Chou 2015 | 6.2 | | **-4.7** | *-0.77* |
| Dong 2022 | 6.2 | *-1.7* | | **-0.27** | Yang 2018 | 6.2 | | **-4.09** | *-0.66* |
| Li 2022 | 6.2 | **-2.1** | | *-0.34* | Dong 2022 | 6.2 | | *-2.44* | **-3.94** |
| Qiu 2023a | 7.3 | *-3.1* | | **-0.42** | Li 2022 | 6.2 | | **-4.898** | *-0.79* |
| Qiu 2023b | 7.3 | *-2.8* | | **-0.38** | Qiu 2023a | 7.3 | | *-1.32* | **-0.18** |
|  |  |  | |  | Qiu 2023b | 7.3 | | *-4.68* | **-0.64** |
| LF-SMA | | | | | HF-SMA | | | | |
| Dong 2022 | 6.2 | *-1.59* | | **-0.25** | Yang 2018 | 6.2 | | **-2.6** | *-0.42* |
| Li 2022 | 6.2 | **-0.31** | | *-0.05* | Dong 2022 | 6.2 | | *-1.67* | **-0.269** |
|  |  |  | |  | Li 2022 | 6.2 | | **-3.78** | *-0.61* |
|  |  |  | |  | Qiu 2023a | 7.3 | | *-0.99* | **-0.136** |
|  |  |  | |  | Qiu 2023b | 7.3 | | *-5.55* | **-0.76** |
| LF-DLPFC | | | | | HF-DLPFC | | | | |
| Qiu 2023a | 7.3 | **-5.53** | | *-0.75* | Yang 2018 | 6.2 | | **-2.29** | *-0.37* |
| Qiu 2023b | 7.3 | **-5.22** | | *-0.71* | Dong 2022 | 6.2 | | *-1.87* | **-0.3** |
|  |  |  | |  | Li 2022 | 6.2 | | **-3.9** | *-0.63* |
|  |  |  | |  | Qiu 2023a | 7.3 | | *1.6* | **0.219** |
|  |  |  | |  | Qiu 2023b | 7.3 | | *-3.17* | **-0.43** |
| LF-OFR | | | | | HF-OFR | | | | |
| Chou 2015 | 6.2 | **-3.1** | | *-0.5* | Chou 2015 | 6.2 | | **-1.42** | *-0.23* |
| HF-M1+DLPFC | | | | | HF-PMD | | | | |
| Yang 2018 | 6.2 | **-0.8** | | *-0.13* | Yang 2018 | 6.2 | | **-0.86** | *-0.14* |
| Dong 2022 | 6.2 | *-7.39* | | **-1.19** |  |  | |  |  |
| Qiu 2023a | 7.3 | *-14.48* | | **-1.98** |  |  | |  |  |
| Qiu 2023b | 7.3 | *-1.37* | | **-0.18** |  |  | |  |  |
| Pharmacological state | | | | | | | | | |
| On state | | | | | Off state | | | | |
| Chou 2015 | 6.2 | **-2.41** | | *-0.39* | Chou 2015 | 6.2 | | **-3.28** | *-0.53* |
| Chung 2016 | 6.2 | **-1.13** | | *-0.183* | Chung 2016 | 6.2 | | **-2.65** | *-0.427* |
| Yang 2018 | 6.2 | **-1.98** | | *-0.32* | Yang 2018 | 6.2 | | **-2.1** | *-0.34* |
| Cheng 2021 | 9.46 | **-0.56** | | *-0.06* | Cheng 2021 | 9.46 | | **-3.49** | *-0.37* |
| Li 2022 | 6.2 | **-4.58** | | *-0.74* | Li 2022 | 6.2 | | **-0.55** | *-0.09* |
| Zhang W 2022 | 21.24 | **-12.32** | | *-0.58* | Zhang W 2022 | 21.24 | | **-7.85** | *-0.37* |
| Liu X 2023a* | 6.2 | **-5.51** | | *-0.89* | Liu X 2023a* | 6.2 | | **-1.73** | *-0.28* |
| Liu X 2023b* | 6.2 | **-0.43** | | *-0.07* | Liu X 2023b* | 6.2 | | **-2.6** | *-0.42* |
| Liu X 2023c* | 6.2 | **-5.022** | | *-0.81* | Liu X 2023c* | 6.2 | | **-0.8** | *-0.13* |
| Liu X 2023d* | 6.2 | **-2.54** | | *-0.41* | Liu X 2023d* | 6.2 | | **-1.92** | *-0.31* |
| Number of sessions | | | | | | | | | |
| Single session | | | | | Multiple sessions | | | | |
| Chung 2016 | 6.2 | **-0.7** | | *-0.114* | Chung 2016 | 6.2 | | **-2.42** | *-0.391* |
| Yang 2018 | 6.2 | **-2.23** | | *-0.36* | Yang 2018 | 6.2 | | **-4.77** | *-0.77* |
| Li 2022 | 6.2 | **-1.36** | | *-0.22* | Li 2022 | 6.2 | | **-4.15** | *-0.67* |

SD= Values of Standard Deviations most representative control group of each meta-analysis; MD= Mean Difference values; SMD= Standardized Mean Difference values. The values in italics are the original effect sizes extracted from the reviews, the values in bold are the converted effect sizes. (a)=short term; (b)= long term. a*= Short-Term/High Frequency; b*= Short-Term/Low Frequency; c*= Long-Term/High Frequency; d*= Long-Term/High Frequency.

**APPENDIX A.8. Strategies to resolve discordances**

## Table A.8.1 Main outcomes

| Outcome | n MA | Author, Year | MD | 95% CI | I^2^ | Concordance > 80% | n latest and better-quality reviews | | Concordance > 80% | n MA with population >200 | | Concordance > 80% | Concordance | Result |
| --- | --- | --- | --- | --- | --- | --- | --- | --- | --- | --- | --- | --- | --- | --- |
| UPDRS-III | 9 | Chou 2015 | -2.85 | [-3.97; -1.8]] | 0 | No [67%] | 2 | - | Yes [100%] | 8 | ✔ | No [75%] | No, latest and better-quality reviews are to be considered | TMS superiority |
|  |  | Xie CL 2015 | -1.83 | [-3.65; 0] | 0 |  |  | - |  |  | ✔ |  |  |  |
|  |  | Wagle Shukla 2016 | -4.1 | [-8.5; 0.1] | N/A |  |  | - |  |  | ✔ |  |  |  |
|  |  | Goodwill 2017 | -2.3 | [-4.09; -0.52] | N/A |  |  | - |  |  | ✔ |  |  |  |
|  |  | Hai-Jiao 2020 | -3.01 | [-7.54; 1.52] | 0 |  |  | - |  |  | - |  |  |  |
|  |  | Dong 2022 | -3.72 | [-5.01; -2.43] | N/A |  |  | ✔ |  |  | ✔ |  |  |  |
|  |  | Li 2022 | -13.59 | [-16.99; -9.98] | 64 |  |  | - |  |  | ✔ |  |  |  |
|  |  | Zhang W 2022 | -2.3 | [-3.48; -1.13] | 42 |  |  | - |  |  | ✔ |  |  |  |
|  |  | Zhang X 2022 | -2.54 | [-3.16; -1.92] | 0 |  |  | ✔ |  |  | ✔ |  |  |  |
| FOG-Q | 8 | Xie YJ 2020 | -4.13 | [-8.57; 0.31] | 71 | No [63%] | 2 | - | Yes [100%] | 8 | ✔ | No [63%] | No, latest and better-quality reviews are to be considered | TMS superiority |
|  |  | Deng 2022a | -0.92 | [-1.64; -0.21] | 0 |  |  | ✔ |  |  | ✔ |  |  |  |
|  |  | Deng 2022b | -2.12 | [-2.75; -1.49] | 15.4 |  |  | ✔ |  |  | ✔ |  |  |  |
|  |  | Krogh 2022 | -1.14 | [-1.67; -0.63] | 10 |  |  | - |  |  | ✔ |  |  |  |
|  |  | Li 2022 | -1.99 | [-3.72; -0.2] | N/A |  |  | - |  |  | ✔ |  |  |  |
|  |  | Qui 2023a | -1.27 | [-3.36; 0.69] | N/A |  |  | - |  |  | ✔ |  |  |  |
|  |  | Qui 2023b | -1.68 | [-4.72; 1.62] | N/A |  |  | - |  |  | ✔ |  |  |  |
|  |  | Liu Z 2024 | -0.47 | [-0.77; -0.18] | 29 |  |  | - |  |  | ✔ |  |  |  |
| TUG | 9 | Xie YJ 2020 | -0.32 | [-0.92; 0.29] | 80 | Yes [89%] | 3 | - | Yes [100%] | 9 | ✔ | Yes [89%] | Yes, sensitivity analysis confirms the result | TMS superiority |
|  |  | Deng 2022a | -1.06 | [-1.55; -0.57] | 0 |  |  | ✔ |  |  | ✔ |  |  |  |
|  |  | Deng 2022b | -1.1 | [-1.42; -0.77] | 0 |  |  | ✔ |  |  | ✔ |  |  |  |
|  |  | Krogh 2022 | -2.06 | [-2.96; -1.17] | N/A |  |  | - |  |  | ✔ |  |  |  |
|  |  | Li 2022 | -0.63 | [-1.01; -0.24] | N/A |  |  | - |  |  | ✔ |  |  |  |
|  |  | Zhang X 2022 | -4.11 | [-4.74; -3.47] | 0 |  |  | ✔ |  |  | ✔ |  |  |  |
|  |  | Qiu 2023a | -2.04 | [-3.26; -0.8] | N/A |  |  | - |  |  | ✔ |  |  |  |
|  |  | Qiu 2023b | -2.66 | [-3.35; -1.77] | N/A |  |  | - |  |  | ✔ |  |  |  |
|  |  | Liu Z 2024 | -0.46 | [-0.72; -0.19] | 25 |  |  | - |  |  | ✔ |  |  |  |
| WT | 6 | Xie YJ 2020 | -0.3* | [-0.57; -0.03] | 24 | Yes [100%] | 2 | - | Yes [100%] | 5 | ✔ | Yes [100%] | Yes, sensitivity analysis confirms the result | TMS superiority |
|  |  | Cheng 2021 | -0.37* | [-0.71; -0.03] | 0 |  |  | - |  |  | - |  |  |  |
|  |  | Deng 2022a | -0.23* | [-0.39; -0.06] | 0 |  |  | ✔ |  |  | ✔ |  |  |  |
|  |  | Deng 2022b | -0.26* | [-0.44; -0.08] | 0 |  |  | ✔ |  |  | ✔ |  |  |  |
|  |  | Li 2022 | -0.95* | [-0.95; -0.61] | N/A |  |  | - |  |  | ✔ |  |  |  |
|  |  | Liu Z 2024 | -0.41* | [-0.41; -0.06] | 7 |  |  | - |  |  | ✔ |  |  |  |

Green coloured boxes indicate a meta-analysis with a positive TMS intervention, orange boxes represent intervention uncertainty and red boxes indicate a favourable intervention for the control group. Symbol legend: * = values given in Standardized Mean Difference; ✔ = the meta-analysis fulfils the criterion; - the meta-analysis does not fulfil the criterion.

## Table A.8.2 UPDRS – III Subgroups

| Subgroup UPDRS-III | n MA | Author, Year | MD | 95% CI | I^2^ | Concordance > 80% | n latest and better-quality reviews | | Concordance > 80% | n MA with population > 200 | | Concordance > 80% | Concordance | Result |
| --- | --- | --- | --- | --- | --- | --- | --- | --- | --- | --- | --- | --- | --- | --- |
| **Timing of outcome measurements** | | | | | | | | | | | | | | |
| Short term | 5 | Chou 2015 | -3.1 | [-4.4; -1.8] | N/A | Yes [100%] | 0 | - | / | 5 | ✔ | Yes [100%] | Yes, sensitivity analysis confirms the result | TMS superiority |
|  |  | Chung 2015 | -1.92 | [-3.16; -0.68] | 32.2 |  |  | - |  |  | ✔ |  |  |  |
|  |  | Wagle Shukla 2016 | -3.5 | [-6.6; -0.3] | N/A |  |  | - |  |  | ✔ |  |  |  |
|  |  | Yang 2018 | -2.29 | [-3.1; -1.49] | 29 |  |  | - |  |  | ✔ |  |  |  |
|  |  | Chen 202 | -2.62 | [-4.18; -1.05] | 0 |  |  | - |  |  | ✔ |  |  |  |
| Long term | 5 | Chou 2015 | -3.66 | [-6.2; -1.18] | N/A | No [60%] | 0 | - | / | 5 | ✔ | No [60%] | No, there are no latest and better-quality reviews | Uncertainty |
|  |  | Chung 2015 | -3.35 | [-5.52; -1.12] | 59.3 |  |  | - |  |  | ✔ |  |  |  |
|  |  | Wagle Shukla 2016 | -4.1 | [-8.4; 0.2] | N/A |  |  | - |  |  | ✔ |  |  |  |
|  |  | Yang 2018 | -2.36 | [-4.03; -0.68] | 55 |  |  | - |  |  | ✔ |  |  |  |
|  |  | Chen 2020 | -1.62 | [-5.04; 1.78] | 0 |  |  | - |  |  | ✔ |  |  |  |
| **Frequency** | | | | | | | | | | | | | | |
| Low frequency (<1 Hz) | 9 | Zhu 2015 | -1.98 | [-3.97; 0] | 47 | No [67%] | 1 | - | / | 9 | ✔ | No [67%] | No, latest and better-quality reviews are to be considered | No difference |
|  |  | Chung 2016 | -1.04 | [-3.33; 1.25] | 0 |  |  | - |  |  | ✔ |  |  |  |
|  |  | Wagle Shukla 2016 | -3.3 | [-5; -1.15] | N/A |  |  | - |  |  | ✔ |  |  |  |
|  |  | Yang 2018 | -1.18 | [-2.6; 0.25] | 32 |  |  | - |  |  | ✔ |  |  |  |
|  |  | Dong 2022 | -1.21 | [-2.45; 0.03] | N/A |  |  | ✔ |  |  | ✔ |  |  |  |
|  |  | Li 2022 | -1.55 | [-2.73; -0.31] | N/A |  |  | - |  |  | ✔ |  |  |  |
|  |  | Zhang W 2022 | -2.12 | [-13.59; 9.35] | 0 |  |  | - |  |  | ✔ |  |  |  |
|  |  | Qiu 2023a | -3.23 | [-11.39; 5.03] | N/A |  |  | - |  |  | ✔ |  |  |  |
|  |  | Qiu 2023b | -4.83 | [-6.42; -3.26] | N/A |  |  | - |  |  | ✔ |  |  |  |
| High frequency (>1 Hz) | 9 | Chung 2015 | -0.42 | [-2.7; 1.87] | 0 | No [78%] | 1 | - | / | 9 | ✔ | No [78%] | No, latest and better-quality reviews are to be considered | TMS superiority |
|  |  | Wagle Shukla 2016 | -3.9 | [-8.4; 0.7] | N/A |  |  | - |  |  | ✔ |  |  |  |
|  |  | Qin 2018 | -2.8 | [-5.45; -0.15] | 0 |  |  | - |  |  | ✔ |  |  |  |
|  |  | Yang 2018 | -2.98 | [-3.97; -1.98] | 45 |  |  | - |  |  | ✔ |  |  |  |
|  |  | Dong 2022 | -4.63 | [-6.25; -3.01] | N/A |  |  | ✔ |  |  | ✔ |  |  |  |
|  |  | Li 2022 | -4.53 | [-5.7; -3.29] | N/A |  |  | - |  |  | ✔ |  |  |  |
|  |  | Zhang W 2022 | -11.89 | [-16.35; -7.22] | 29 |  |  | - |  |  | ✔ |  |  |  |
|  |  | Qiu 2023a | -5.21 | [-9.26; -1.23] | N/A |  |  | - |  |  | ✔ |  |  |  |
|  |  | Qiu 2023b | -4.74 | [-6.45; -3.05] | N/A |  |  | - |  |  | ✔ |  |  |  |
| **Application site** | | | | | | | | | | | | | | |
| M1 | 3 | Chung 2016 | -3.5 | [-6.21; -0.79] | 55.6 | Yes [100%] | 0 | - | / | 3 | ✔ | Yes [100%] | Yes, sensitivity analysis confirms the result | TMS superiority |
|  |  | Yang 2018 | -3.22 | [-4.46; -1.98] | 42 |  |  | - |  |  | ✔ |  |  |  |
|  |  | Li 2022 | -3.97 | [-5.39; -2.6] | N/A |  |  | - |  |  | ✔ |  |  |  |
| DLPFC | 3 | Chung 2016 | 0.5 | [-2.44; 3.43] | 0 | No [67%] | 0 | - | / | 3 | ✔ | No [67%] | No, there are no latest and better-quality reviews | Uncertainty |
|  |  | Yang 2018 | -1.8 | [-4.22; 0.68] | 25 |  |  | - |  |  | ✔ |  |  |  |
|  |  | Li 2022 | -5.02 | [-8.25; -1.74] | N/A |  |  | - |  |  | ✔ |  |  |  |
| SMA | 3 | Chung 2016 | -1.83 | [-3.53; -0.13] | 0 | Yes [100%] | 0 | - | / | 3 | ✔ | Yes [100%] | Yes, sensitivity analysis confirms the result | TMS superiority |
|  |  | Yang 2018 | -1.86 | [-3.22; -0.5] | 35 |  |  | - |  |  | ✔ |  |  |  |
|  |  | Li 2022 | -2.6 | [-4.22; -1.05] | N/A |  |  | - |  |  | ✔ |  |  |  |
| M1 + DLPFC | 1 | Yang 2018 | -0.62 | [-3.41; 2.17] | 0 | / | 0 |  | / | 1 | ✔ | / | Only one meta-analysis | No difference |
| **Frequency – Application site** | | | | | | | | | | | | | | |
| LF - M1 | 5 | Chou 2015 | -1.74 | [-4.84; 1.43] | N/A | Yes [80%] | 1 | - | / | 5 | ✔ | Yes [80%] | Yes, sensitivity analysis confirms the result | No difference |
|  |  | Dong 2022 | -1.7 | [-4.28; 0.89] | N/A |  |  | ✔ |  |  | ✔ |  |  |  |
|  |  | Li 2022 | -2.11 | [-4.03; -0.19] | N/A |  |  | - |  |  | ✔ |  |  |  |
|  |  | Qiu 2023a | -3.1 | [-9.49; 3.29] | N/A |  |  | - |  |  | ✔ |  |  |  |
|  |  | Qiu 2023b | -2.8 | [-9.34; 3.47] | N/A |  |  | - |  |  | ✔ |  |  |  |
| LF - DLPFC | 2 | Qiu 2023a | -5.53 | [-13.29; 2.23] | N/A | No [50%] | 0 | - | / | 2 | ✔ | No [50%] | No, there are no latest and better-quality reviews | Uncertainty |
|  |  | Qiu 2023b | -5.22 | [-6.89; -3.55] | N/A |  |  | - |  |  | ✔ |  |  |  |
| LF - SMA | 2 | Dong 2022 | -1.59 | [-3.74; 0.55] | N/A | Si [100%] | 1 | ✔ | / | 2 | ✔ | Si [100%] | Yes, sensitivity analysis confirms the result | No difference |
|  |  | Li 2022 | -0.31 | [-5.08; 4.4] | N/A |  |  | - |  |  | ✔ |  |  |  |
| LF - OFR | 1 | Chou 2015 | -3.1 | [-5.39; -0.81] | N/A | / | 0 | - | / | 1 |  | / | Only one meta-analysis | TMS superiority |
| HF - M1 | 6 | Chou 2015 | -4.77 | [-6.7; -2.85] | N/A | Yes [83%] | 1 | - | / | 6 | ✔ | Yes [83%] | Yes, sensitivity analysis confirms the result | TMS superiority |
|  |  | Yang 2018 | -4.09 | [-6.32; -1.8] | 56 |  |  | - |  |  | ✔ |  |  |  |
|  |  | Dong 2022 | -2.44 | [-4.14; -0.74] | N/A |  |  | ✔ |  |  | ✔ |  |  |  |
|  |  | Li 2022 | -4.9 | [-6.63; -3.22] | N/A |  |  | - |  |  | ✔ |  |  |  |
|  |  | Qiu 2023a | -1.32 | [-5.05; 2.41] | 93 |  |  | - |  |  | ✔ |  |  |  |
|  |  | Qiu 2023b | -4.68 | [-7.01; -2.34] | 0 |  |  | - |  |  | ✔ |  |  |  |
| HF - DLPFC | 5 | Yang 2018 | -2.29 | [-4.84; 0.25] | 0 | No [60%] | 1 | - | / | 5 | ✔ | No [60%] | No, latest and better-quality reviews are to be considered | No difference |
|  |  | Dong 2022 | -1.87 | [-4.1; 0.35] | N/A |  |  | ✔ |  |  | ✔ |  |  |  |
|  |  | Li 2022 | -3.91 | [-6.63; -1.24] | N/A |  |  | - |  |  | ✔ |  |  |  |
|  |  | Qiu 2023a | 1.6 | [0.38; 2.82] | N/A |  |  | - |  |  | ✔ |  |  |  |
|  |  | Qiu 2023b | -3.17 | [-8.04; 1.7] | N/A |  |  | - |  |  | ✔ |  |  |  |
| HF - SMA | 5 | Yang 2018 | -2.6 | [-5.77; 0.56] | 65 | No [60%] | 1 | - | / | 5 | ✔ | No [60%] | No, latest and better-quality reviews are to be considered | TMS superiority |
|  |  | Dong | -1.67 | [-2.95; -0.39] | N/A |  |  | ✔ |  |  | ✔ |  |  |  |
|  |  | Li 2022 | -3.78 | [-5.95; -1.67] | N/A |  |  | - |  |  | ✔ |  |  |  |
|  |  | Qiu 2023a | -0.99 | [-7.38; 5.41] | 93 |  |  | - |  |  | ✔ |  |  |  |
|  |  | Qiu 2023b | -5.55 | [-8.59; -2.51] | N/A |  |  | - |  |  | ✔ |  |  |  |
| HF - M1 + DLPFC | 4 | Yang 2018 | -0.81 | [-4.28; 2.67] | 0 | No [50%] | 1 | - | / | 4 | ✔ | No [50%] | No, latest and better-quality reviews are to be considered | TMS superiority |
|  |  | Dong 2022 | -7.39 | [-13.25; -1.53] | N/A |  |  | ✔ |  |  | ✔ |  |  |  |
|  |  | Qiu 2023a | -14.48 | [-16.04; -12.93] | 0 |  |  | - |  |  | ✔ |  |  |  |
|  |  | Qiu 2023b | -1.37 | [-5.38; 2.64] | N/A |  |  | - |  |  | ✔ |  |  |  |
| HF - OFR | 1 | Chou 2015 | -1.43 | [-2.98; 0.12] | N/A | / | 0 | - | / | 1 | ✔ | / | Only one meta-analysis | No difference |
| HF - PMD | 1 | Yang 2018 | -0.87 | [-6.45; 4.71] | N/A | / | 1 | ✔ | / | 1 | ✔ | / | Only one meta-analysis | No difference |
| **Pharmacological state** | | | | | | | | | | | | | | |
| On | 10 | Chou 2015 | -2.42 | [-3.72; -1.05] | N/A | No [70%] | 0 | - | / | 9 | ✔ | No [78%] | No, there are not latest or better- quality reviews and  MAs with populations >200 are discordant | Uncertainty |
|  |  | Chung 2016 | -1.13 | [-2.26; -0.01] | 0 |  |  | - |  |  | ✔ |  |  |  |
|  |  | Yang 2018 | -1.98 | [-2.98; -1.05] | 37 |  |  | - |  |  | ✔ |  |  |  |
|  |  | Cheng 2021 | -0.57 | [-3.5; 2.37] | 0 |  |  | - |  |  | - |  |  |  |
|  |  | Li 2022 | -4.59 | [-5.95; -3.22] | N/A |  |  | - |  |  | ✔ |  |  |  |
|  |  | Zhang W 2022 | -12.32 | [-18.05; -6.58] | 43 |  |  | - |  |  | ✔ |  |  |  |
|  |  | Liu X 2023a | -5.52 | [-8.8; -2.17] | 77.4 |  |  | - |  |  | ✔ |  |  |  |
|  |  | Liu X 2023b | -0.43 | [-3.72; 2.79] | N/A |  |  | - |  |  | ✔ |  |  |  |
|  |  | Liu X 2023c | -5.02 | [-7.56; -2.48] | 67.5 |  |  | - |  |  | ✔ |  |  |  |
|  |  | Liu X 2023d | -2.54 | [-5.52; 0.43] | N/A |  |  | - |  |  | ✔ |  |  |  |
| Off | 10 | Chou 2015 | -3.29 | [-4.9; -1.67] | N/A | No [60%] | 0 | - | / | 9 | ✔ | No [50%] | No, there are not latest or better- quality reviews and  MAs with populations >200 are discordant | Uncertainty |
|  |  | Chung 2016 | -2.65 | [-4.62; -0.67] | 40.5 |  |  | - |  |  | ✔ |  |  |  |
|  |  | Yang 2018 | -2.11 | [-3.35; -0.93] | 6 |  |  | - |  |  | ✔ |  |  |  |
|  |  | Cheng 2021 | -3.5 | [-6.15; -0.85] | 19 |  |  | - |  |  | - |  |  |  |
|  |  | Li 2022 | -0.56 | [-2.17; 1.05] | N/A |  |  | - |  |  | ✔ |  |  |  |
|  |  | Zhang W 2022 | -7.86 | [-14.66; -1.06] | 0 |  |  | - |  |  | ✔ |  |  |  |
|  |  | Liu X 2023a | -1.74 | [-7.69; 4.28] | 76.0 |  |  | - |  |  | ✔ |  |  |  |
|  |  | Liu X 2023b | -2.6 | [-5.08; -0.12] | 0 |  |  | - |  |  | ✔ |  |  |  |
|  |  | Liu X 2023c | -0.81 | [-5.08; 3.53] | 64.4 |  |  | - |  |  | ✔ |  |  |  |
|  |  | Liu X 2023d | -1.92 | [-4.84; 0.93] | 0 |  |  | - |  |  | ✔ |  |  |  |
| **Number of sessions** | | | | | | | | | | | | | | |
| Single session | 3 | Chung 2016 | -0.71 | [-2.75; 1.33] | 0 | Yes [100%] | 0 | - | / | 3 | ✔ | Yes [100%] | Yes, sensitivity analysis confirms the result | No difference |
|  |  | Yang 2018 | -2.23 | [-5.27; 0.74] | 0 |  |  | - |  |  | ✔ |  |  |  |
|  |  | Li 2022 | -1.36 | [-4.34; 1.67] | N/A |  |  | - |  |  | ✔ |  |  |  |
| Multiple sessions | 3 | Chung 2016 | -2.42 | [-4; -0.86] | 47.3 | Yes [100%] | 0 | - | / | 3 | ✔ | Yes [100%] | Yes, sensitivity analysis confirms the result | TMS superiority |
|  |  | Yang 2018 | -4.77 | [-7.75; -1.74] | 67 |  |  | - |  |  | ✔ |  |  |  |
|  |  | Li 2022 | -4.15 | [-5.27; -3.1] | N/A |  |  | - |  |  | ✔ |  |  |  |

Green coloured boxes indicate a meta-analysis with a positive TMS intervention, orange boxes represent intervention uncertainty and red boxes indicate a favourable intervention for the control group. Symbol legend: * = values given in Standardized Mean Difference; ✔ = the meta-analysis fulfils the criterion; - the meta-analysis does not fulfil the criterion; / = non-calculable value. Acronyms legend: M1= primary motor cortex; DLPFC= dorsolateral prefrontal cortex; SMA= supplementary motor area; OFR=orbitofrontal cortex; PMD= dorsal premotor area; LF= low frequency; HF= high frequency.

# **APPENDIX A.9. Summary of evidence of included SRs on UPDRS – III subgroups**

## Figure A.9.1 Summary of evidence of included SRs on UPDRS – III: Short term


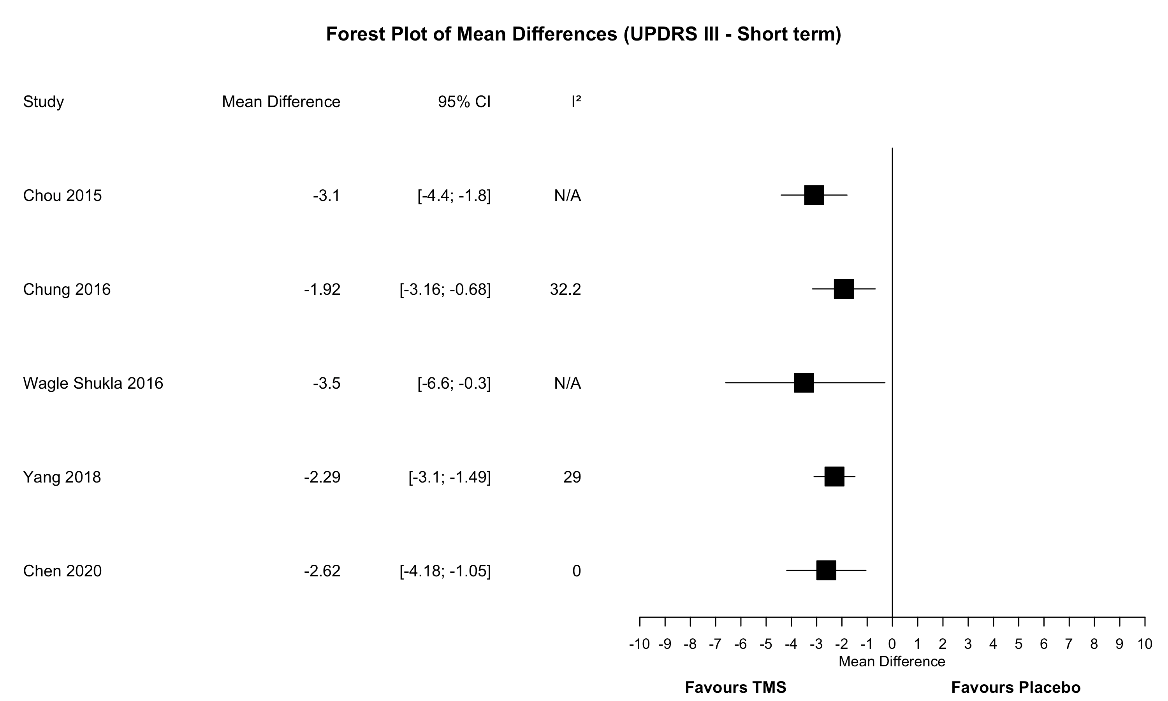


Forest plot of the Mean Difference for the UPDRS-III (Short Term) outcome. Values to the left of the non-difference line are favourable to TMS, those to the right are favourable to placebo. CI = confidence interval, I^2^= heterogeneity.

## Figure A.9.2 Summary of evidence of included SRs on UPDRS – III: Long term


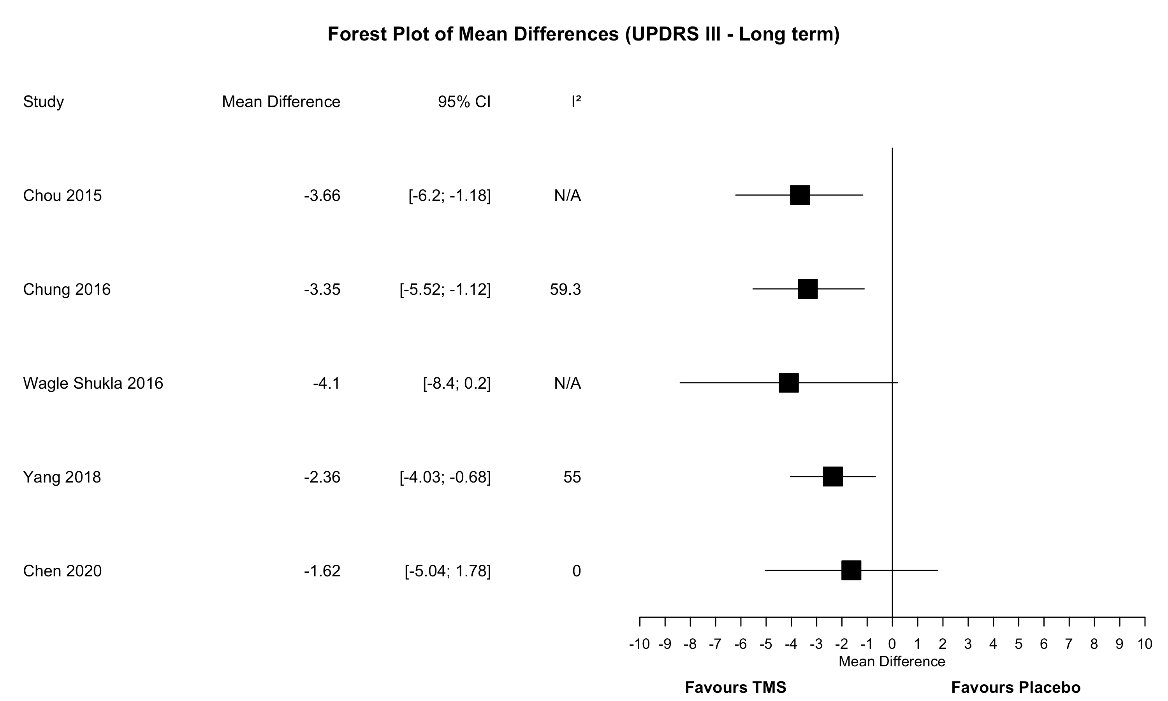


Forest plot of the Mean Difference for the UPDRS-III (Long Term) outcome. Values to the left of the non-difference line are favourable to TMS, those to the right are favourable to placebo. CI = confidence interval, I^2^= heterogeneity.

## Figure A.9.3 Summary of evidence of included SRs on UPDRS – III: Low frequency


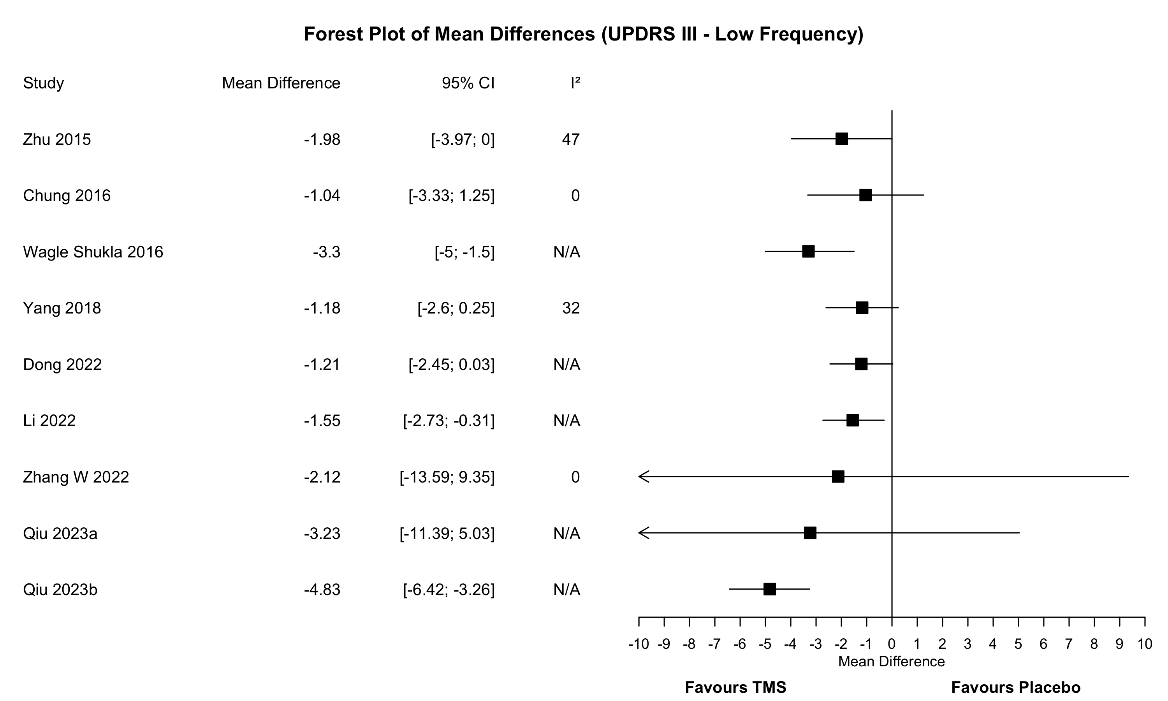


Forest plot of the Mean Difference for the UPDRS-III (Low frequency) outcome. Values to the left of the non-difference line are favourable to TMS, those to the right are favourable to placebo. CI = confidence interval, I^2^= heterogeneity.

## Figure A.9.4 Summary of evidence of included SRs on UPDRS – III: High frequency


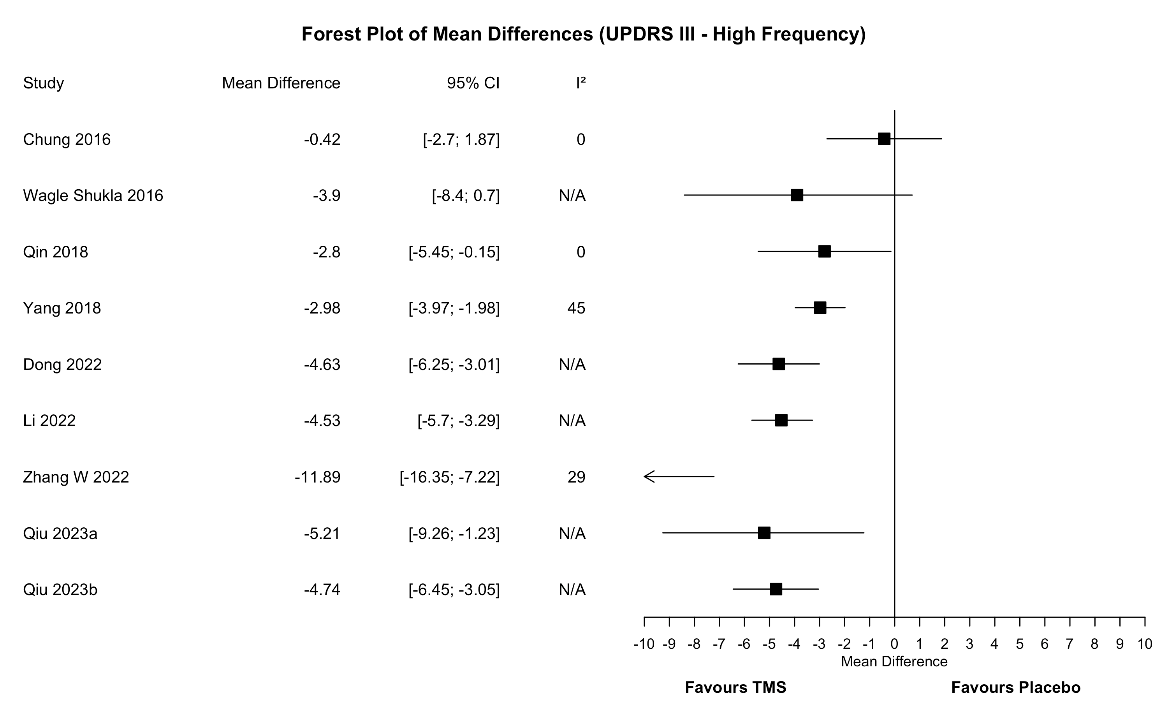


Forest plot of the Mean Difference for the UPDRS-III (High frequency) outcome. Values to the left of the non-difference line are favourable to TMS, those to the right are favourable to placebo. CI = interval of confidence, I^2^= heterogeneity.

## Figure A.9.5 Summary of evidence of included SRs on UPDRS – III: M1


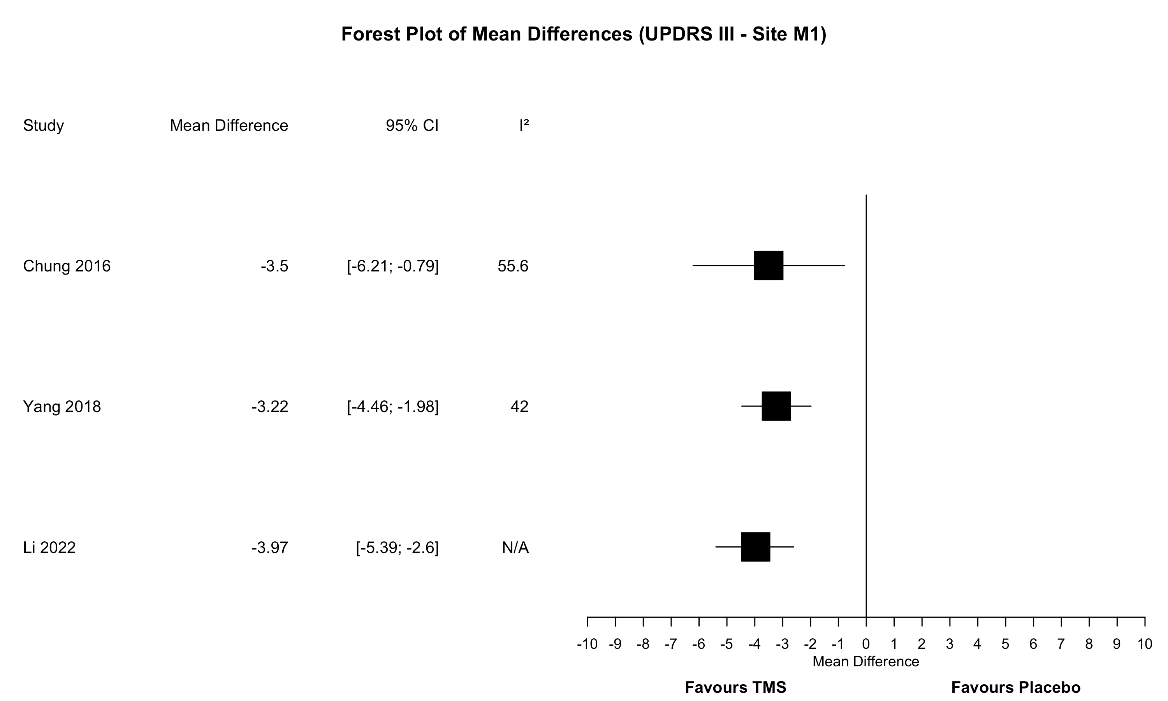


Forest plot of the Mean Difference for the UPDRS-III (M1) outcome. Values to the left of the non-difference line are favourable to TMS, those to the right are favourable to placebo. CI = confidence interval, I^2^= heterogeneity.

## Figure A.9.6 Summary of evidence of included SRs on UPDRS – III: DLPFC


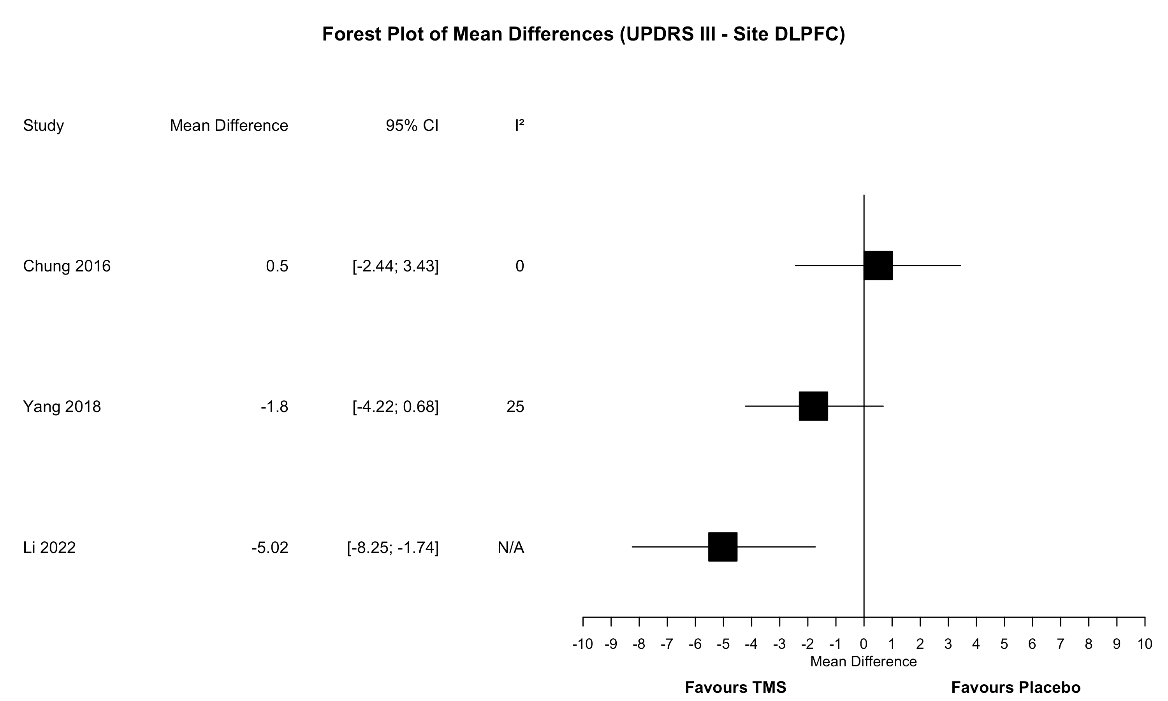


Forest plot of the Mean Difference for the UPDRS-III (DLPFC) outcome. Values to the left of the non-difference line are favourable to TMS, those to the right are favourable to placebo. CI = confidence interval, I^2^= heterogeneity.

## Figure A.9.7 Summary of evidence of included SRs on UPDRS – III: SMA


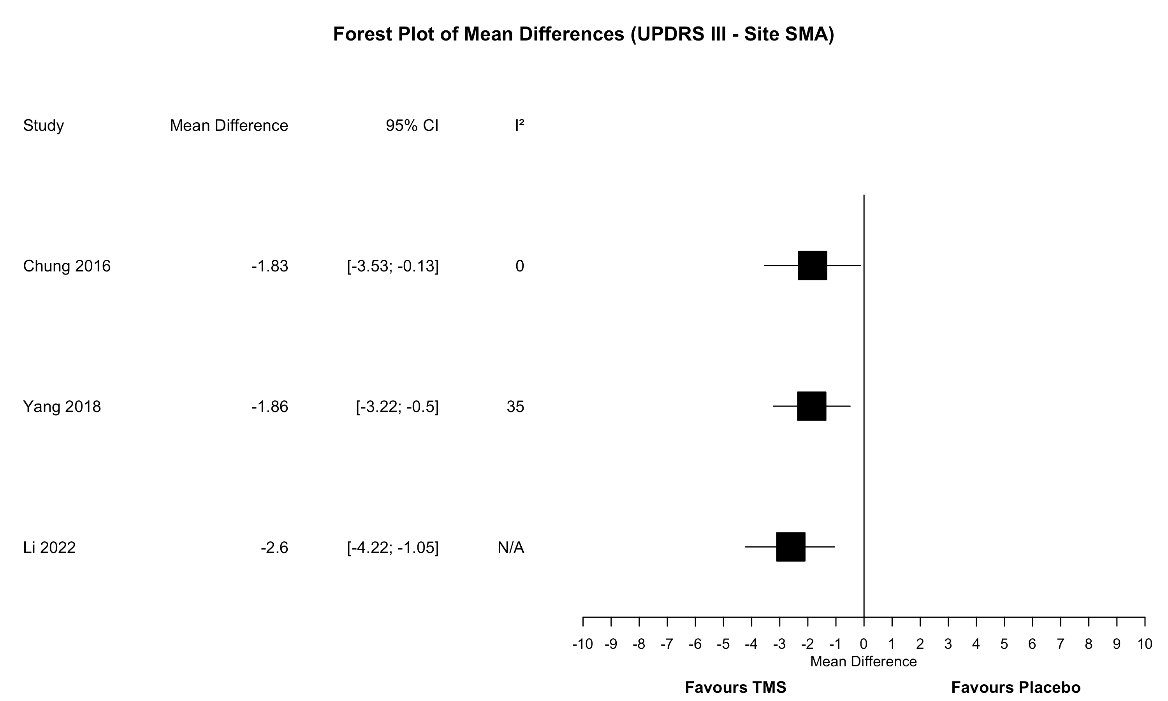


Forest plot of the Mean Difference for the UPDRS-III (SMA) outcome. Values to the left of the non-difference line are favourable to TMS, those to the right are favourable to placebo. CI = confidence interval, I^2^= heterogeneity.

## Figure A.9.8 Summary of evidence of included SRs on UPDRS – III: M1+DLPFC


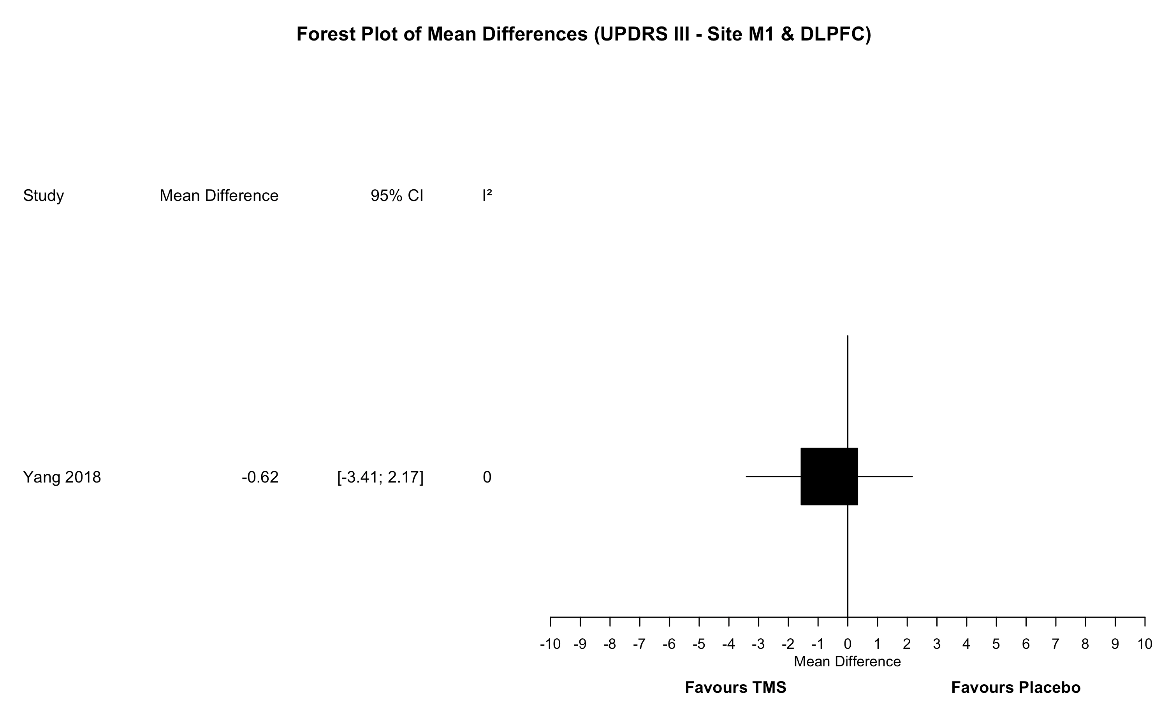


Forest plot of the Mean Difference for the UPDRS-III (M1+DLPFC) outcome. Values to the left of the non-difference line are favourable to TMS, those to the right are favourable to placebo. CI = confidence interval, I^2^= heterogeneity.

## Figure A.9.9 Summary of evidence of included SRs on UPDRS – III: LF-M1


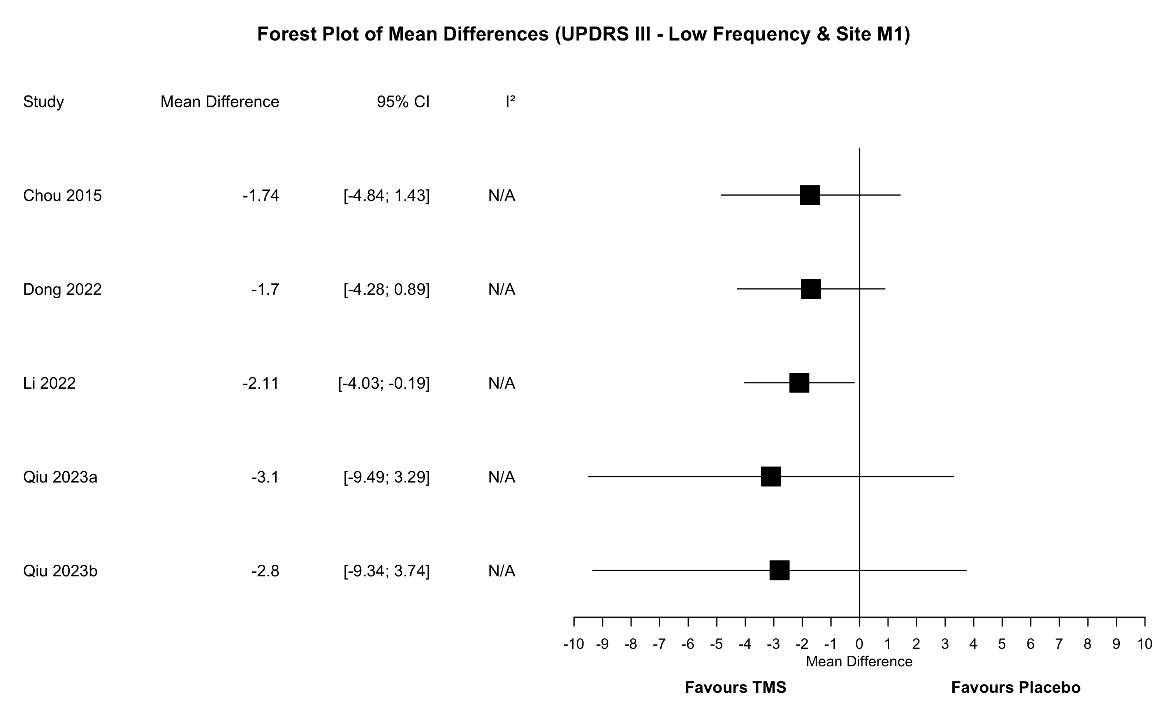


Forest plot of the Mean Difference for the UPDRS-III (LF-M1) outcome. Values to the left of the non-difference line are favourable to TMS, those to the right are favourable to placebo. CI = confidence interval, I^2^= heterogeneity.

## Figure A.9.10 Summary of evidence of included SRs on UPDRS – III: LF-DLPFC


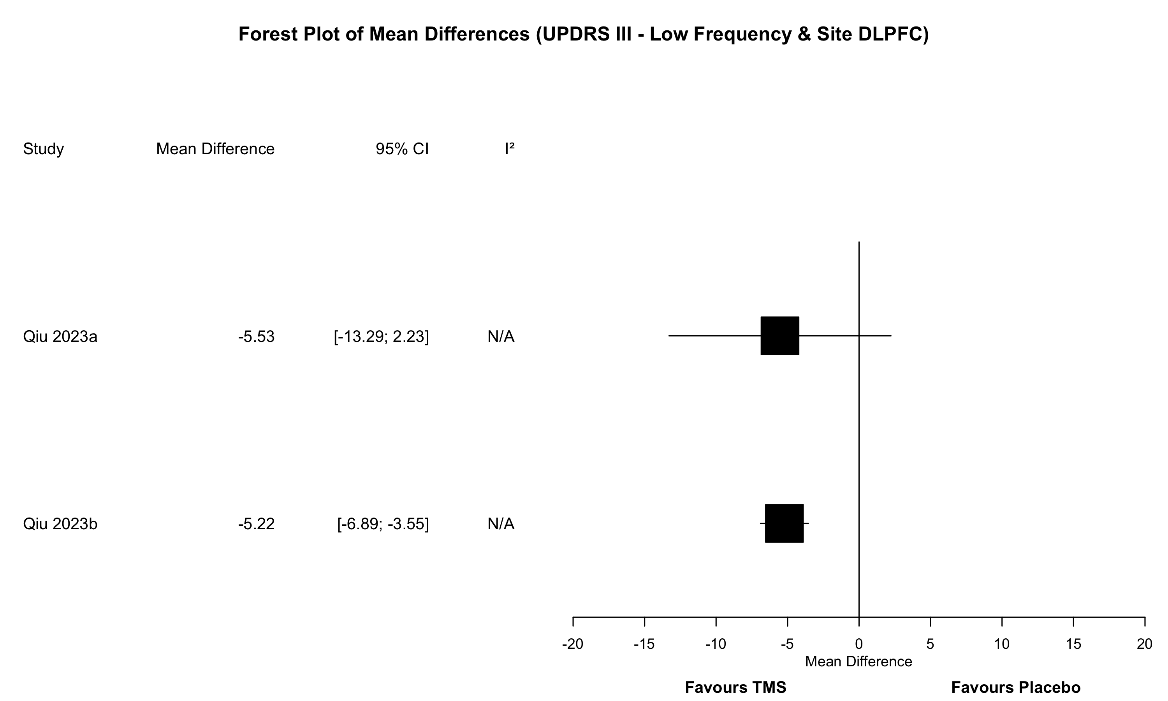


Forest plot of the Mean Difference for the UPDRS-III (LF-DLPFC) outcome. Values to the left of the non-difference line are favourable to TMS, those to the right are favourable to placebo. CI = confidence interval, I^2^= heterogeneity.

## Figure A.9.11 Summary of evidence of included SRs on UPDRS – III: LF-SMA


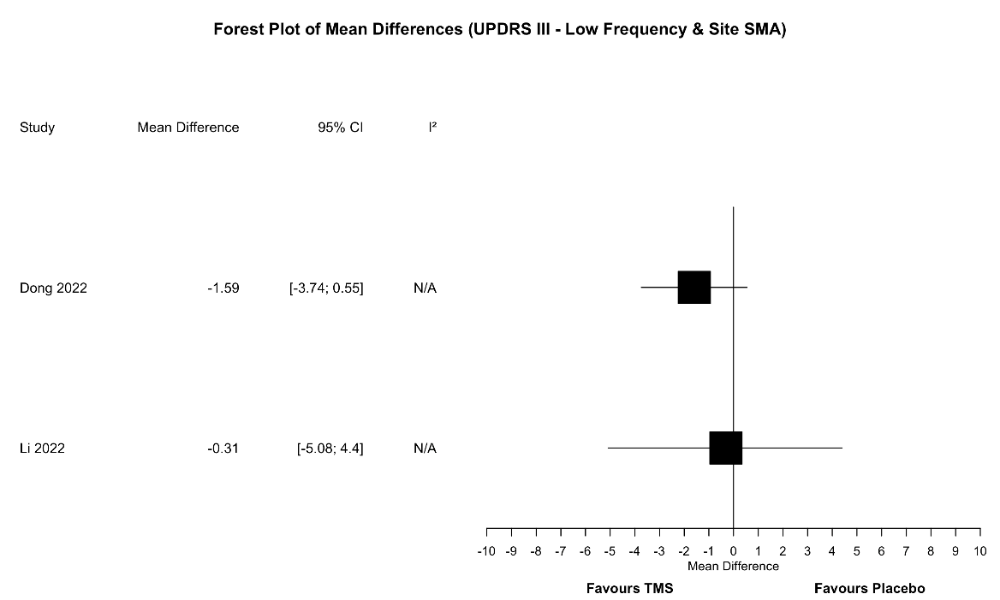


Forest plot of the Mean Difference for the UPDRS-III (LF-SMA) outcome. Values to the left of the non-difference line are favourable to TMS, those to the right are favourable to placebo. CI = confidence interval, I^2^= heterogeneity.

## Figure A.9.12 Summary of evidence of included SRs on UPDRS – III: LF-OFR


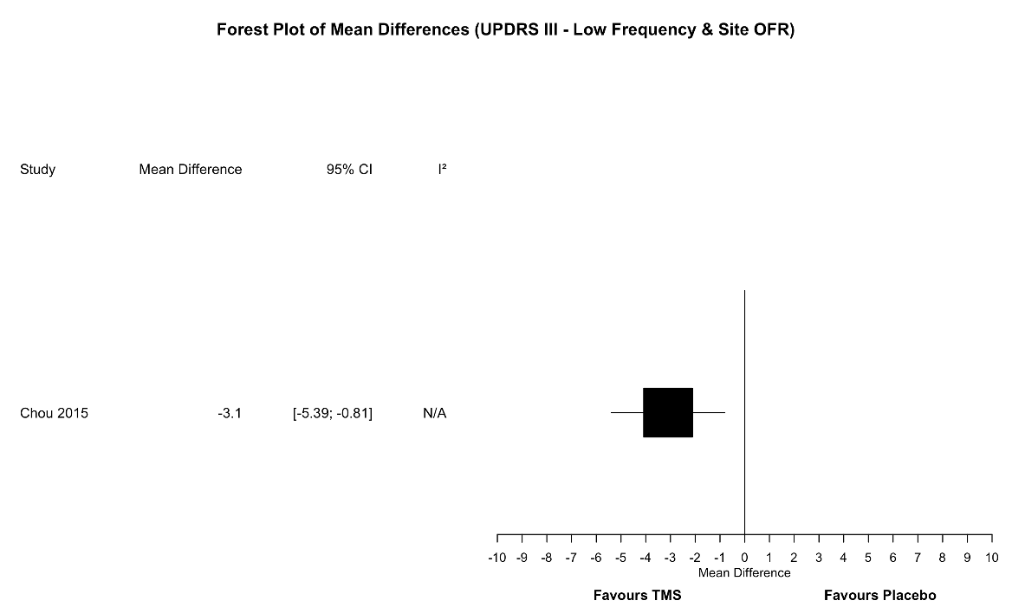


Forest plot of the Mean Difference for the UPDRS-III (LF-OFR) outcome. Values to the left of the non-difference line are favourable to TMS, those to the right are favourable to placebo. CI = confidence interval, I^2^= heterogeneity.

## Figure A.9.13 Summary of evidence of included SRs on UPDRS – III: HF-M1


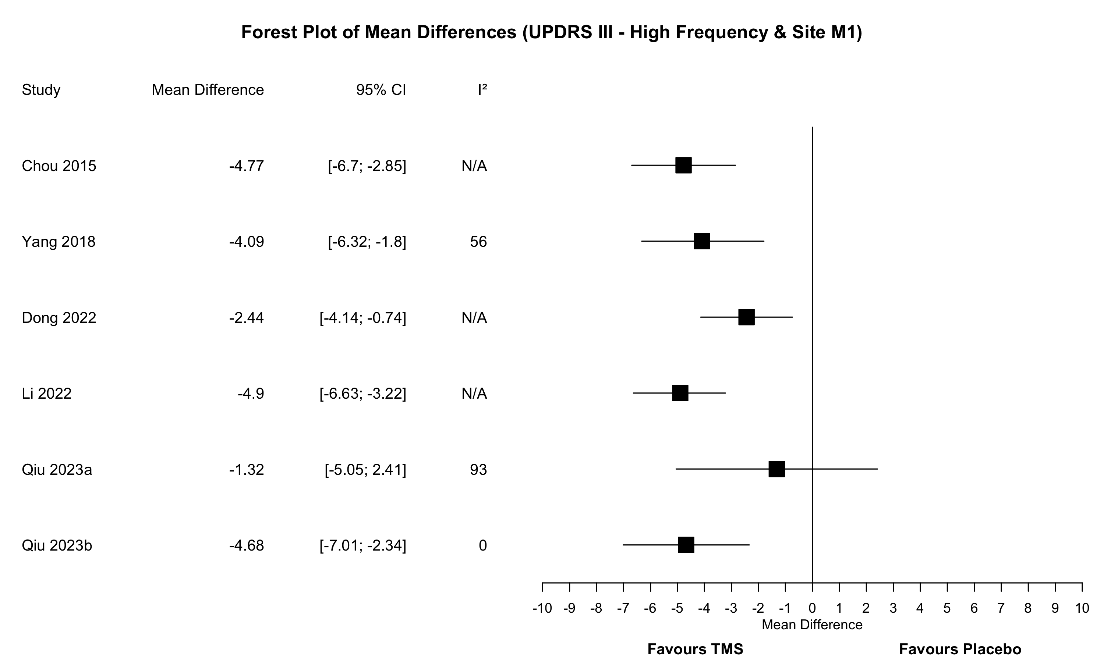


Forest plot of the Mean Difference for the UPDRS-III (HF-M1) outcome. Values to the left of the non-difference line are favourable to TMS, those to the right are favourable to placebo. CI = confidence interval, I^2^= heterogeneity.

## Figure A.9.14 Summary of evidence of included SRs on UPDRS – III: HF-DLPFC


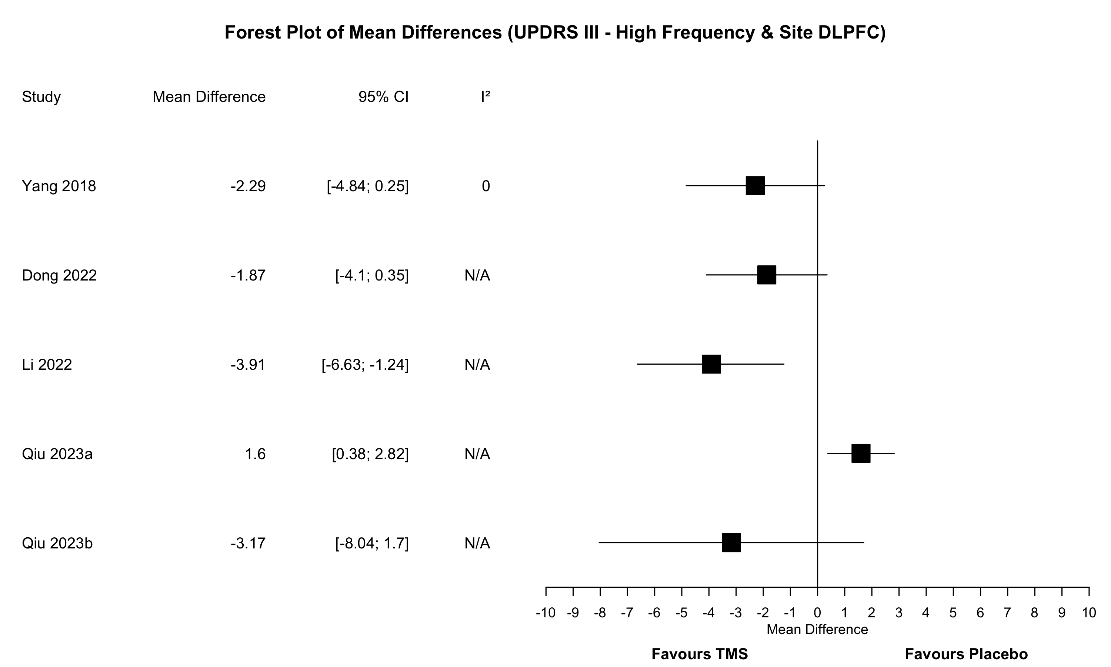


Forest plot of the Mean Difference for the UPDRS-III (HF-DLPFC) outcome. Values to the left of the non-difference line are favourable to TMS, those to the right are favourable to placebo. CI = confidence interval, I^2^= heterogeneity.

## Figure A.9.15 Summary of evidence of included SRs on UPDRS – III: HF-SMA


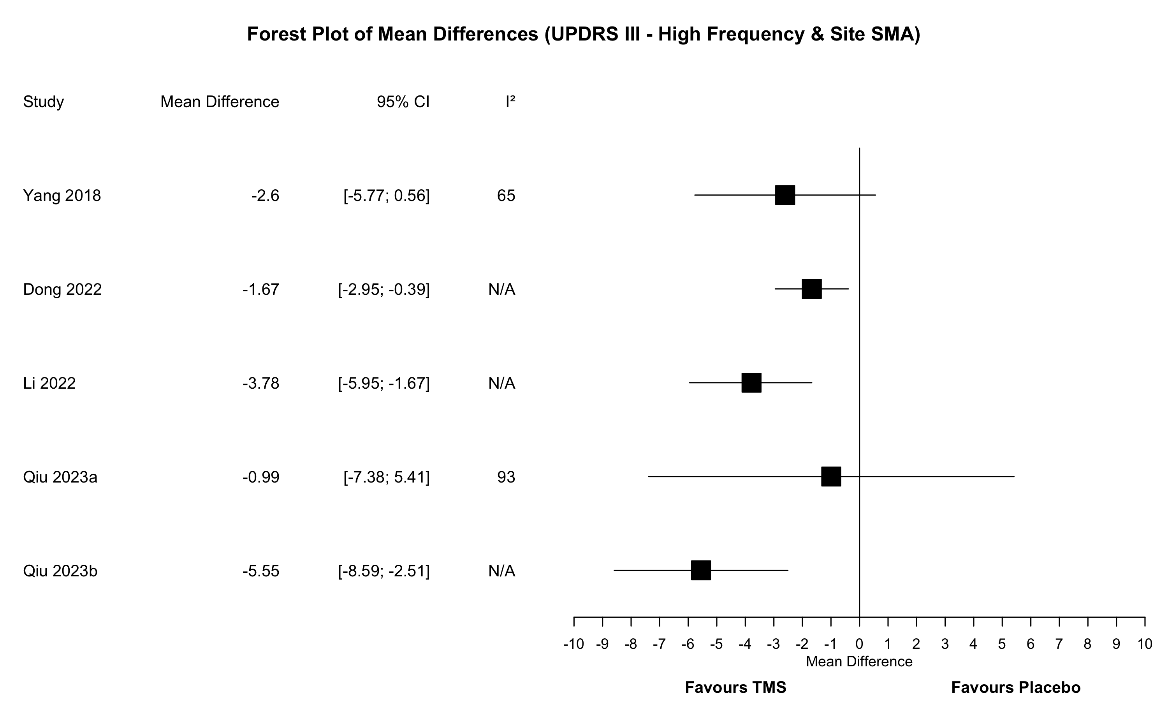


Forest plot of the Mean Difference for the UPDRS-III (HF-SMA) outcome. Values to the left of the non-difference line are favourable to TMS, those to the right are favourable to placebo. CI = confidence interval, I^2^= heterogeneity.

## Figure A.9.16 Summary of evidence of included SRs on UPDRS – III: HF-M1+DLPFC


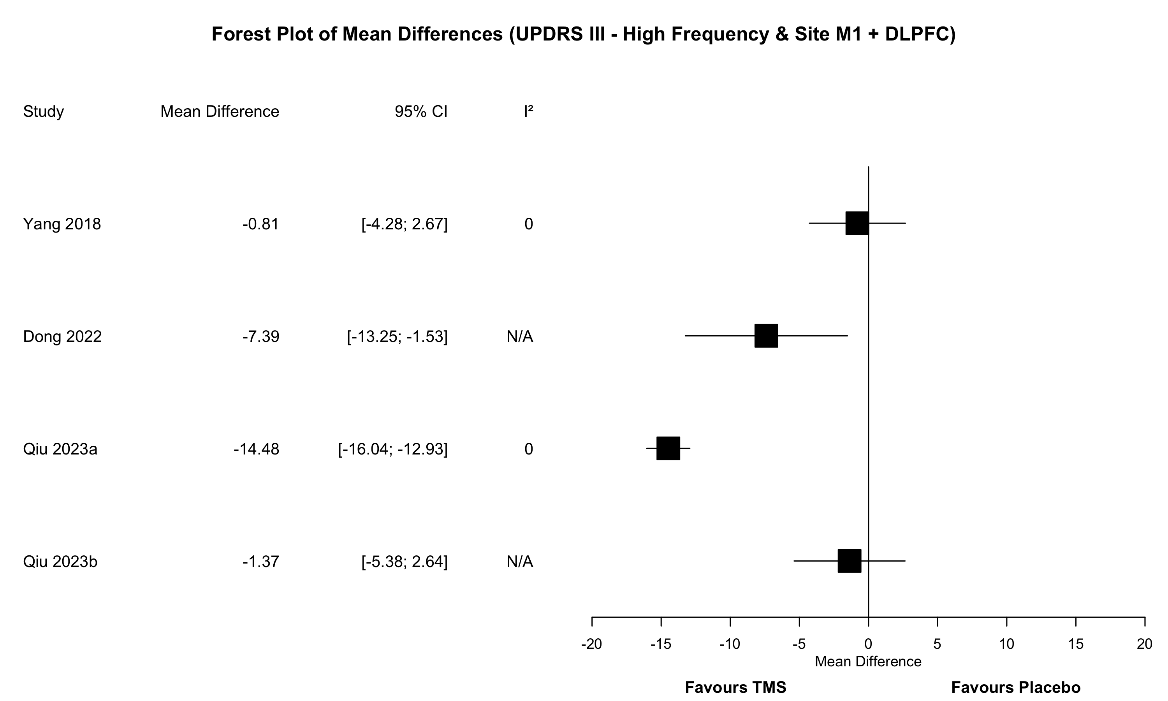


Forest plot of the Mean Difference for the UPDRS-III (HF-M1+DLPFC) outcome. Values to the left of the non-difference line are favourable to TMS, those to the right are favourable to placebo. CI = confidence interval, I^2^= heterogeneity.

## Figure A.9.17 Summary of evidence of included SRs on UPDRS – III: HF-OFR


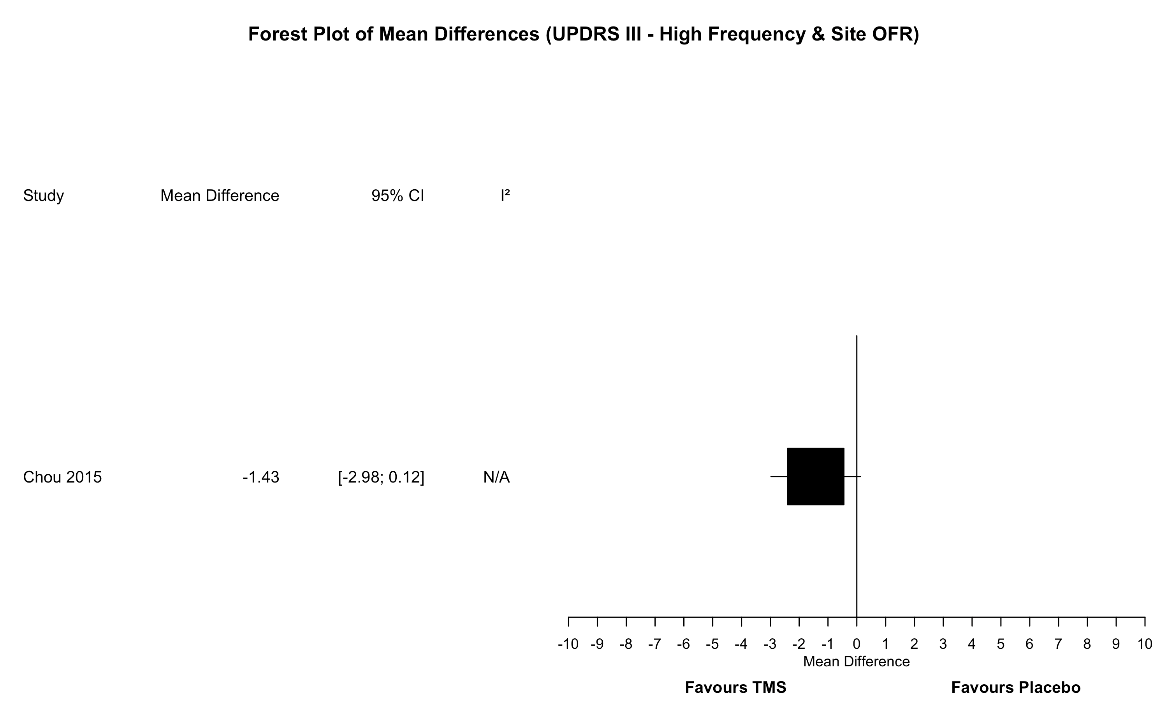


Forest plot of the Mean Difference for the UPDRS-III (HF-OFR) outcome. Values to the left of the non-difference line are favourable to TMS, those to the right are favourable to placebo. CI = confidence interval, I^2^= heterogeneity.

## Figure A.9.18 Summary of evidence of included SRs on UPDRS – III: HF-PMD


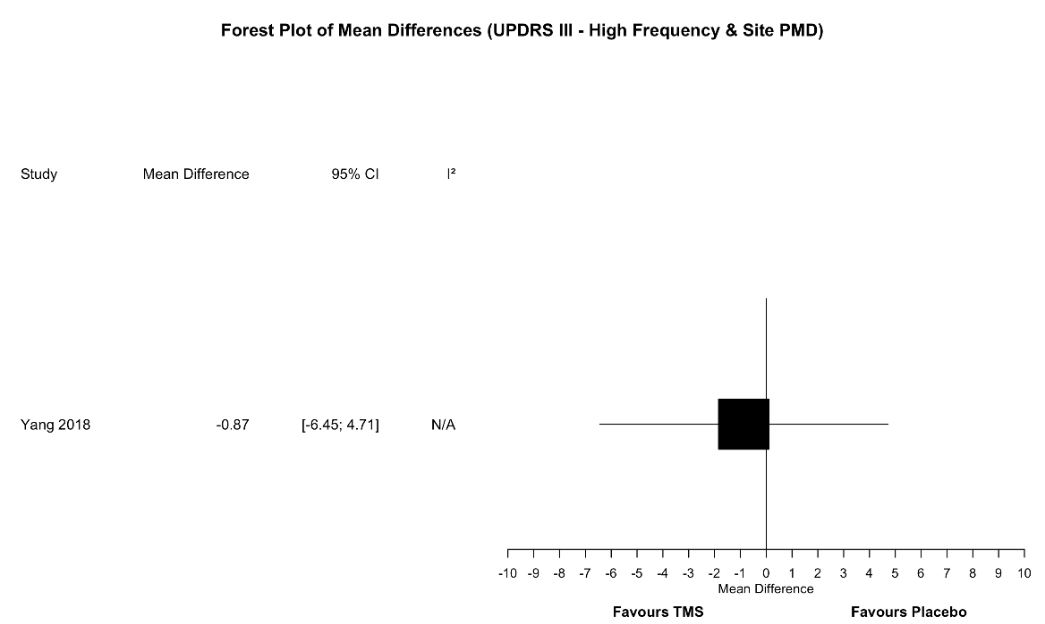


Forest plot of the Mean Difference for the UPDRS-III (HF-PMD) outcome. Values to the left of the non-difference line are favourable to TMS, those to the right are favourable to placebo. CI = confidence interval, I^2^= heterogeneity.

## Figure A.9.19 Summary of evidence of included SRs on UPDRS – III: On-state


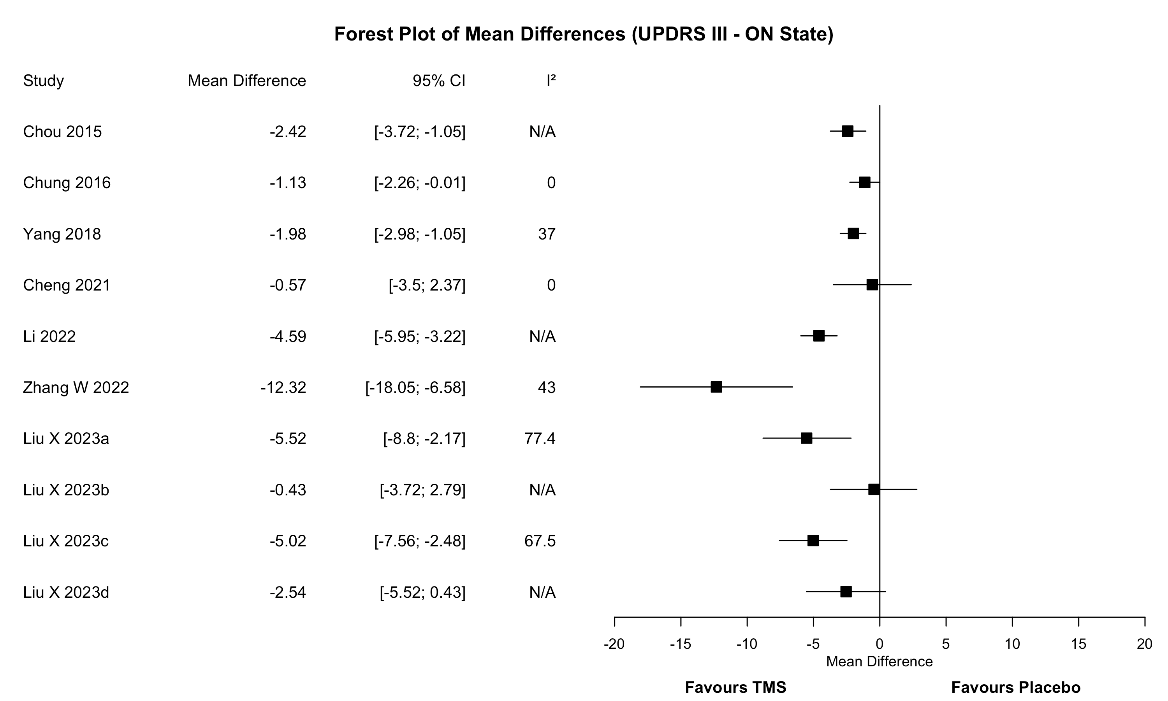


Forest plot of the Mean Difference for the UPDRS-III (On-state) outcome. Values to the left of the non-difference line are favourable to TMS, those to the right are favourable to placebo. CI = confidence interval, I^2^= heterogeneity.

## Figure A.9.20 Summary of evidence of included SRs on UPDRS – III: Off-state


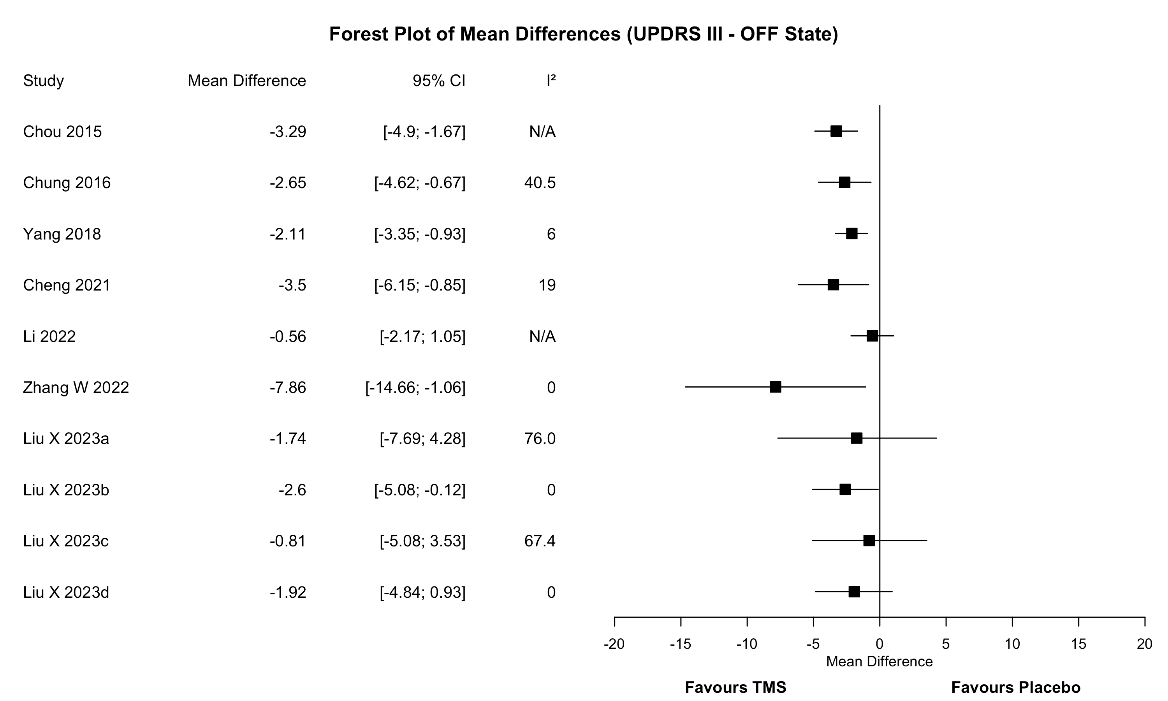


Forest plot of the Mean Difference for the UPDRS-III (Off-state) outcome. Values to the left of the non-difference line are favourable to TMS, those to the right are favourable to placebo. CI = confidence interval, I^2^= heterogeneity.

## Figure A.9.21 Summary of evidence of included SRs on UPDRS – III: Single session


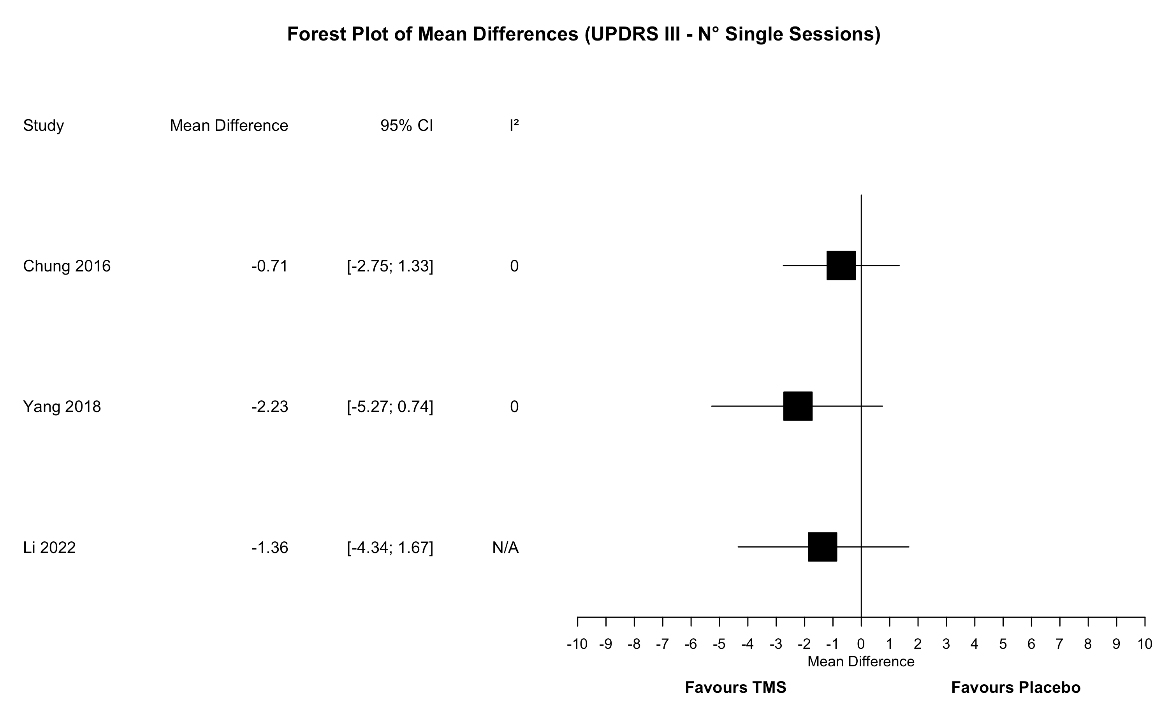


Forest plot of the Mean Difference for the UPDRS-III (Single session) outcome. Values to the left of the non-difference line are favourable to TMS, those to the right are favourable to placebo. CI = confidence interval, I^2^= heterogeneity.

## Figure A.9.22 Summary of evidence of included SRs on UPDRS – III: Multiple sessions


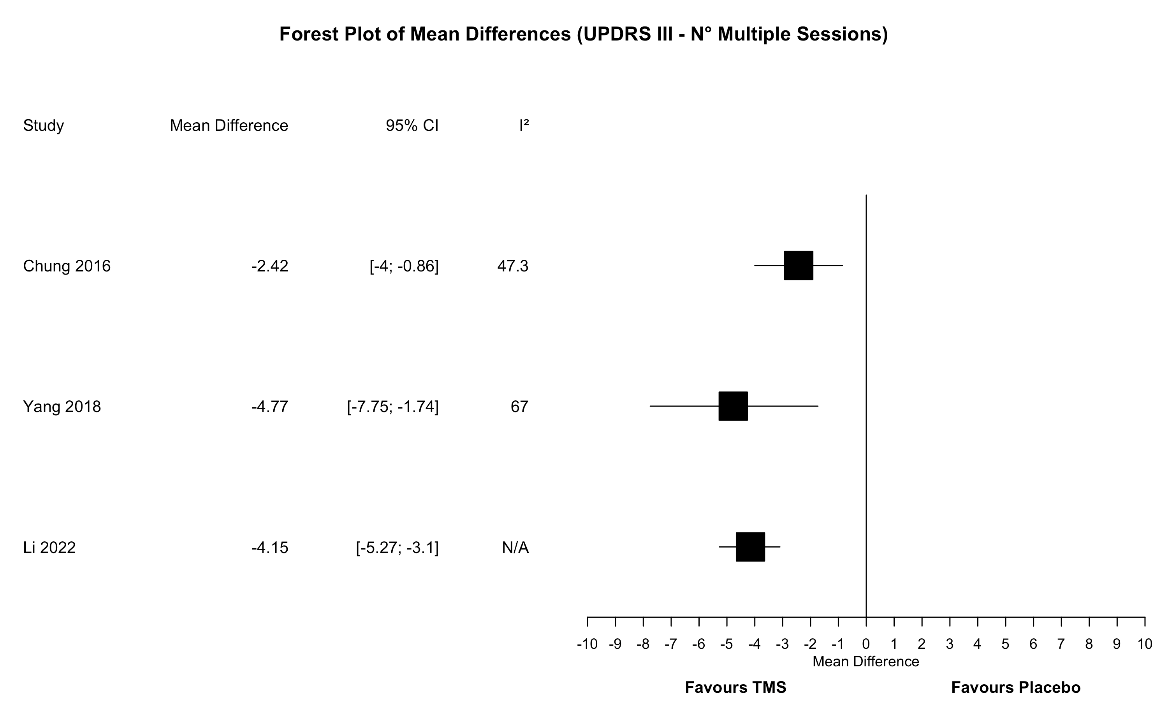


Forest plot of the Mean Difference for the UPDRS-III (Multiple sessions) outcome. Values to the left of the non-difference line are favourable to TMS, those to the right are favourable to placebo. CI = confidence interval, I^2^= heterogeneity.

**APPENDIX A.10. Certainty of evidence in meta-analysis**

## Table A.10.1 GRADE

| **Review** | **Number of partecipants** | **I^2^** | **AMSTAR** | **RoB** | GRADE |
| --- | --- | --- | --- | --- | --- |
| Chou 2015 | No serious | Serious | Serious | No serious | MODERATE |
| Xie, C.L. 2015 | No serious | No serious | Very serious | Serious | LOW |
| Zhu 2015 | No serious | No serious | Very serious | No serious | MODERATE |
| Chung 2016 | No serious | No serious | Very serious | Serious | LOW |
| Wagle Shukla 2016 | No serious | Serious | Very serious | Serious | LOW |
| Goodwill 2017 | No serious | Serious | Very serious | No serious | LOW |
| Qin 2018 | No serious | No serious | Very serious | Serious | LOW |
| Yang 2018 | No serious | No serious | Very serious | No serious | MODERATE |
| Chen 2020 | No serious | No serious | Very serious | Serious | LOW |
| Hai-Jiao 2020 | Serious | No serious | Very serious | No serious | LOW |
| Xie,Y. J. 2020 | No serious | Serious | Very serious | No serious | LOW |
| Cheng 2021 | Serious | No serious | Very serious | No serious | LOW |
| Deng 2022 | No serious | No serious | Serious | Serious | MODERATE |
| Dong 2022 | No serious | Serious | Serious | No serious | MODERATE |
| Krogh 2022 | No serious | Serious | Very serious | No serious | LOW |
| Li 2022 | No serious | Serious | Very serious | No serious | LOW |
| Zhang, W. 2022 | No serious | No serious | Very serious | No serious | MODERATE |
| Zhang, X. 2022 | No serious | No serious | Serious | Serious | MODERATE |
| Liu, X. 2023 | No serious | Serious | Very serious | No serious | LOW |
| Qiu 2023 | No serious | Serious | Very serious | Serious | LOW |
| Liu, Z. 2024 | No serious | No serious | Very serious | No serious | MODERATE |

# **APPENDIX A.11. Adverse events**

## Table A.11.1 Summary of reported adverse events

| ADVERSE EVENT | Chou 2015 | Wagle Shukla 2016 | Yang 2018 | Chen 2020 | Cheng 2021 | Liu X 2023 | Qiu 2023 | Liu Z 2024 |
| --- | --- | --- | --- | --- | --- | --- | --- | --- |
| *Headache* | x | x | x | x | - | x | x | x |
| *Coil exposure pain* | x | - | x | - | - | - | - | x |
| *Neck pain* | - | x | x | x | - | x | x | - |
| *Burning sensation* | - | x | - | - | - | - | x | - |
| *Increased salivation* | - | x | - | - | - | - | - | - |
| *Tinnitus* | - | - | x | - | - | - | - | - |
| *Local discomfort* | - | - | x | - | x | - | - | x |
| *Vertigo* | - | - | - | x | - | x | - | x |
| *Constipation* | - | - | - | x | - | - | - | - |
| *Nausea* | - | - | - | x | - | x | - | x |
| *General weakness* | - | - | - | - | - | x | - | - |
| *Aggravation of walking disorders* | - | - | - | - | - | x | - | - |
